# Tetrahedral DNA Nanostructure‐Based Biomimetic Nanovesicles Attenuate Sepsis‐Associated ARDS by Suppressing Glycolysis via the BMAL1/PFKFB3 Axis

**DOI:** 10.1002/advs.202523782

**Published:** 2026-04-20

**Authors:** Yunlong Zhang, Bin Li, Zhijin Fan, Yan Yan, Fei Ma, Changting He, Shiping Liu, Mingliang Pan, Zhou Pan, Huijuan Wang, Xinting Fu, Jiamei Wang, Yue Jia, Qin Gu, Duo Jiang, Xueting Liu, Bohua Ren, Qinqin Wang, Yuehua Hei, Han Duan, Yanqiu Wu, Zihui Wei, Liying Zhan, Yuhui Liao

**Affiliations:** ^1^ Department of Critical Care Medicine & Department of Emergency Renmin Hospital of Wuhan University Wuhan Hubei China; ^2^ School of Inspection Ningxia Medical University Yinchuan Ningxia China; ^3^ School of Basic Medical Sciences Ningxia Medical University Yinchuan Ningxia China; ^4^ Institute for Engineering Medicine Kunming Medical University Kunming Yunnan China; ^5^ Department of Microbiology School of Public Health Southern Medical University Guangzhou Guangdong China

**Keywords:** anti‐inflammatory and antioxidant, biomimetic nanovesicle, BMAL1, metabolic reprogramming, sepsis‐associated acute respiratory distress syndrome, tetrahedral DNA nanostructure

## Abstract

Sepsis‐associated acute respiratory distress syndrome (SA‐ARDS) is a life‐threatening complication characterized by excessive pulmonary inflammation and pulmonary edema, lacking effective treatments. This study identifies the transcription factor BMAL1 in alveolar macrophages (AMs) as a key therapeutic target. Mechanistically, BMAL1 represses the expression of the glycolytic enzyme PFKFB3 by binding to the *Pfkfb3* promoter, thereby inhibiting glycolysis, M1 polarization of AMs, and the generation of pro‐inflammatory cytokines and reactive oxygen species (ROS). Based on this regulatory mechanism, a biomimetic nanoplatform, RM@TNT, is engineered for precise SA‐ARDS therapy. Fabricated by hybridizing AM membrane‐derived nanovesicles with ROS‐responsive liposomes, the nanoplatform encapsulates tetrahedral DNA nanostructures (TNT) preloaded with nobiletin (Nob, a BMAL1 agonist) and Tuftsin (an AM‐targeting peptide). Following inhalation, the AM membrane tropism of RM@TNT ensures prolonged pulmonary retention, prompting targeted TNT release within the ROS‐rich pathological microenvironment. Tuftsin then precisely delivers TNT to AMs, where Nob is intracellularly released to activate BMAL1. This activation upregulates the BMAL1/PFKFB3 axis, suppressing AM glycolysis, inflammation, and oxidative stress. Treatment with RM@TNT resulted in significantly attenuated lung inflammation, injury, and edema, along with markedly improved survival in SA‐ARDS mice. Collectively, this multimodal, targeted metabolic reprogramming approach is a highly promising therapeutic strategy for SA‐ARDS.

## Graphical abstract

Upon inhalation, RM@TNT could persist long‐term in the diseased lungs, while undergoing disintegration to release TNT specifically within the ROS‐rich pathological microenvironments of SA‐ARDS. The released TNT was then precisely delivered to AMs via Tuftsin, where it released Nob intracellularly to activate BMAL1 expression, thus inhibiting AM glycolysis, M1 polarization, and the massive production of multiple pro‐inflammatory cytokines and ROS through the BMAL1/PFKFB3 axis.

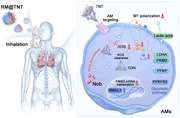

## 1 Introduction

Sepsis‐associated acute respiratory distress syndrome (SA‐ARDS) is a severe complication of sepsis characterized by diffuse alveolar damage and excessive inflammatory infiltration, which leads to acute hypoxemia and severe pulmonary oedema [1]. Clinical data indicate that the 30‐day mortality rate among septic patients who develop ARDS increases from 17.4% to 27.3%, positioning this condition as one of the leading causes of death in sepsis [2, 3]. Current treatment strategies for SA‐ARDS focus on lung‐protective mechanical ventilation, restrictive fluid management, and neuromuscular blockade, with no targeted pharmacological interventions available [4, 5]. Despite advancements in anti‐inflammatory therapies, endothelial barrier stabilization, and precision critical care management, the mortality rate of SA‐ARDS remains alarmingly high [6, 7, 8]. Consequently, there is an urgent need to develop more effective therapeutic strategies for SA‐ARDS to alleviate the global burden of sepsis.

The pathophysiology of SA‐ARDS is closely associated with metabolic dysregulation [9], in which macrophages play a critical role [10, 11, 12]. Specifically, activated macrophages rapidly switch from oxidative phosphorylation to aerobic glycolysis (the Warburg effect) in response to inflammatory stimuli, such as lipopolysaccharide (LPS) and interferon‐γ [13, 14, 15]. This metabolic shift provides the energy required for macrophages to adopt a pro‐inflammatory state, including M1 polarization and the production of pro‐inflammatory cytokines [16]. Additionally, enhanced glycolysis in macrophages results in elevated lactic acid production, which shows a significant positive correlation with mortality in septic patients [17]. Therefore, reversing the metabolic transformation of macrophages (i.e., inhibiting glycolysis and enhancing oxidative phosphorylation) may represent a promising therapeutic approach for SA‐ARDS, sepsis, and other inflammatory diseases with similar pathophysiological processes.

Brain and muscle ARNT‐like 1 (BMAL1), a core transcription factor involved in circadian rhythm regulation, has been reported to have the potential to inhibit cellular glycolysis [18, 19]. Previous studies have revealed that BMAL1 levels in the blood of sepsis patients are dramatically reduced [20]. In vitro experiments demonstrated that LPS stimulation downregulates BMAL1 expression in murine macrophages [21, 22]. Thus, precisely upregulating BMAL1 expression in macrophages may potentially suppress their glycolytic activity. However, the underlying molecular mechanism by which BMAL1 regulates macrophage glycolysis in SA‐ARDS remains unexplored. Nobiletin (Nob), a polymethoxyflavonoid abundant in citrus fruits, has emerged as a potential therapeutic agent for various diseases, including sepsis and acute lung injury [23, 24]. Moreover, Nob can upregulate BMAL1 expression by directly binding to and activating retinoic acid receptor‐related orphan receptors within the core circadian clock oscillator [25, 26]. Based on these findings, we hypothesize that BMAL1 is a key therapeutic target for SA‐ARDS and that Nob acts as a BMAL1 agonist; targeted delivery of Nob to macrophages can specifically upregulate BMAL1 expression, thereby suppressing macrophage glycolysis and ultimately achieving effective treatment of SA‐ARDS.

Recent advances in nanotechnology have opened new frontiers in medical applications [27]. Tetrahedral DNA nanostructures (TDNs) have emerged as highly promising nanomaterials due to their flexible structural programmability, inherent biocompatibility, efficient cellular internalization, and versatile drug‐loading capacity, making them ideal nanocarriers for peptides, nucleic acids, small molecules, and monomers [28, 29, 30] To date, numerous studies have investigated the application of TDNs as drug delivery vehicles for the treatment of various diseases. For example, TDNs have been used to deliver puerarin, baicalin, and microRNAs for the management of osteoporosis, fibrotic diseases, and sepsis, etc. [31, 32, 33]. Of note, TDNs exhibit intrinsic reactive oxygen species (ROS)‐scavenging properties, enabling them to mitigate oxidative stress while delivering drugs—making them particularly suitable for inflammatory diseases [34], including SA‐ARDS.

**SCHEME 1:**
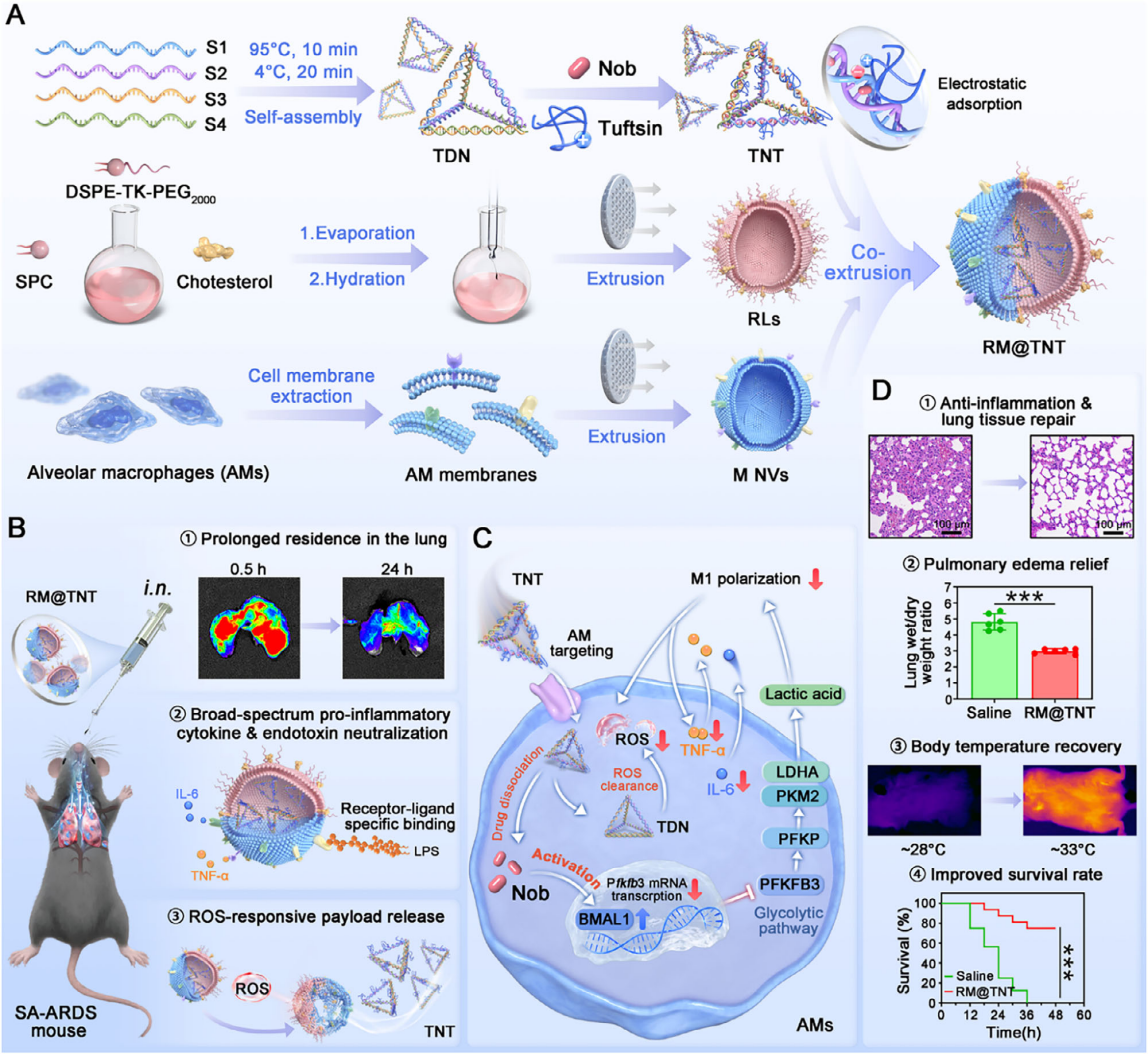
(A) Schematic diagram of the preparation of TNT, RLs, M NVs and RM@TNT. (B) RM@TNT enables prolonged residence in the lung, broad‐spectrum neutralization of pro‐inflammatory cytokines and LPS, and ROS‐responsive cargo release following intranasal administration (i.n.). (C) Schematic diagram illustrating the mechanism by which TNT precisely inhibits glycolysis, M1 polarization, inflammatory responses, and oxidative stress in AMs (metabolic reprogramming pattern). (D) Therapeutic outcomes of RM@TNT on SA‐ARDS mice. TDN: tetrahedral DNA nanostructures; Nob: nobiletin; RLs: ROS‐responsive liposomes. M NVs: AM membrane‐based nanovesicles.

In this study, we first synthesized TDNs through a self‐assembly approach. We then used TDN as carriers to load Nob through co‐incubation. To achieve specific targeting of alveolar macrophages (AMs), the central immune cells in SA‐ARDS pathophysiology, TDNs were functionalized with Tuftsin, a peptide known for its AM‐targeting properties [35], via electrostatic adsorption, ultimately creating TNT (Scheme 1A). To improve the in vivo delivery efficiency and bioavailability of TNT, biomimetic nanovesicles were developed by fusing ROS‐responsive liposomes (RLs) with AM membrane‐derived biomimetic nanovesicles (M NVs) to encapsulate TNT, thus forming RM@TNT (Scheme 1A). Following intranasal administration, this nanosystem achieves prolonged residence in the lungs of SA‐ARDS mice by leveraging the inflammatory tropism of the carried AM membranes. In addition, the high expression of multiple pro‐inflammatory cytokine receptors (e.g., TNF‐α and IL‐6 receptors) and pattern recognition receptors (e.g., Toll‐like receptor 4, TLR4 [36]) on AM cell membranes allows them to act as molecular decoys that sequester and neutralize inflammatory mediators such as pro‐inflammatory cytokines (such as TNF‐α and IL‐6) and endotoxins (such as LPS), hereby exerting broad‐spectrum anti‐inflammatory effects (Scheme 1B). Simultaneously, the ROS‐responsive liposome components of RM@TNT enable spatiotemporally TNT release specifically in the high‐ROS pathological microenvironments (Scheme 1B) [37]. The released TNT then precisely targets AMs via Tuftsin and is taken up by AMs, where it liberates Nob intracellularly to upregulate BMAL1 expression. The increased BMAL1 directly binds to the promoter region of *Pfkfb3* (*6‐phosphofructo‐2‐kinase/fructose‐2,6‐bisphosphatase 3*) to suppress its transcription, leading to reduced production of the key glycolytic enzyme PFKFB3 and blocking downstream glycolytic cascade reactions (Scheme 1C). Such glycolysis‐inhibiting regimen significantly suppressed M1 polarization of AMs in the course of SA‐ARDS, meanwhile attenuating excessive production of various pro‐inflammatory cytokines and ROS. Concurrently, TDN's inherent ROS scavenging activity synergistically reduces intracellular ROS levels (Scheme 1C). After treatment with RM@TNT, SA‐ARDS mice exhibited dramatically alleviated lung inflammation, repaired lung injury, reduced pulmonary edema, restored body temperature, and a markedly improved survival rate (Scheme 1D). Taken together, this nanoengineered strategy represents a promising candidate for the management of SA‐ARDS in clinical settings.

## 2 Results

### 2.1 BMAL1 is Downregulated in the Lung Tissue and AMs of SA‐ARDS Mice

**FIGURE 1:**
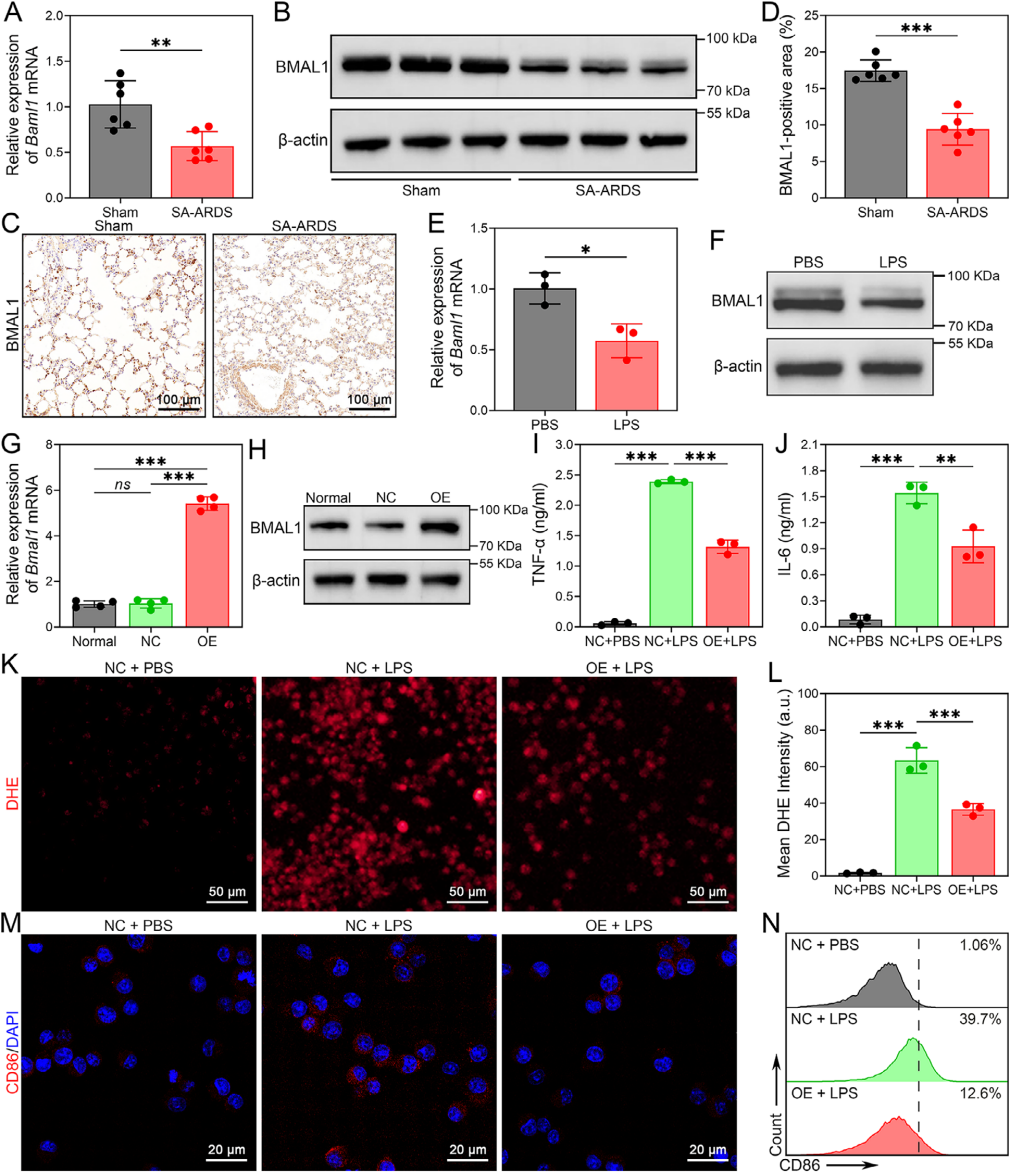
Critical role of BMAL1 in AMs during SA‐ARDS. (A,B) Comparison of *Bmal1* mRNA expressions (via RT‐qPCR) and BMAL1 protein expressions (through Western blot) in lung tissues between the sham and SA‐ARDS mice (*n* = 6). (C,D) Immunohistochemical (IHC) and quantitative analysis for BMAL1 (*n* = 6) in lung tissues of the sham and SA‐ARDS mice. (E,F) Comparison of *Bmal1* mRNA expressions (*n* = 3) and BMAL1 protein expressions in MH‐S cells before and after 24 h of LPS (1 µg·mL^−^^1^) stimulation. (G,H) Comparison of *Bmal1* mRNA expressions (*n* = 4) and BMAL1 protein expressions among normal MH‐S cells, MH‐S cells transfected with a negative control (NC) plasmid, and MH‐S cells transfected BMAL1‐overexpressing (OE) plasmid through lentiviral transduction. (I,J) Comparison of concentrations of TNF‐α and IL‐6 secreted from MH‐S cells upon different treatments (*n* = 3). **G1**: NC + PBS (NC MH‐S cells treated with PBS for 24 h); **G2**: NC + LPS (NC MH‐S cells treated with LPS (1 µg·mL^−^^1^) for 24 h); **G3**: OE + LPS (BMAL1‐OE MH‐S cells treated with LPS (1 µg·mL^−^^1^) for 24 h). (K,L) Fluorescence microscopic imaging and mean fluorescence intensity quantification (*n* = 3) of DHE (an intracellular ROS probe)‐stained MH‐S cells from different groups indicated in I. (M,N) Immunofluorescence and flow cytometry analyses of CD86 expression in MH‐S cells from different groups indicated in I. Quantitative data are presented as mean ± standard deviation (SD). Statistical significance between two groups was determined by two‐sided unpaired *t*‐test, while comparisons among three groups were analyzed using one‐way analysis of variance (ANOVA) with post‐hoc corrections. ^*^ *p* <0.05, ^**^ *p* <0.01, ^***^ *p* <0.001; ns: no significant difference.

BMAL1 represents a potential therapeutic target for SA‐ARDS. Accordingly, we assessed its expression in lung tissue, the primary organ affected in this disease. The SA‐ARDS mouse model was established via the standard cecal ligation and puncture (CLP) procedure, which has been validated for its stability and reproducibility and is characterized by severe lung injury (Figure S1A,B), pulmonary edema (Figure S1C), and a marked increase in the generation of various pro‐inflammatory cytokines (such as TNF‐α and IL‐6) (Figure S1D,E). As shown in Figure 1A,B and Figure S2, both the gene and protein expression levels of BMAL1 were dramatically reduced in the lung tissues of SA‐ARDS mice compared to those of mice that underwent laparotomy with immediate closure (sham). Immunohistochemical analysis (for BMAL1) of lung tissues confirmed a similar trend (Figure 1C,D).

To determine whether BMAL1 expression in AMs is affected, the mouse AM cell line MH‐S was employed as a model system. Concurrently, LPS stimulation of MH‐S cells was conducted to mimic the pathological state of AMs in SA‐ARDS mice. As shown in Figure 1E,F, and Figure S3, LPS treatment induced downregulation of BMAL1, evident at both the transcriptional and translational levels in MH‐S cells. Collectively, these findings indicate that BMAL1 is downregulated in lung tissues and AMs of SA‐ARDS mice, which may play an important role in the development and progression of the disease.

### 2.2 BMAL1 Upregulation Suppresses LPS‐Induced Inflammatory Responses in AMs

To investigate the role of BMAL1 in AMs, we constructed a BMAL1‐overexpressing (OE) MH‐S cell line via lentiviral transduction (Figure 1G,H; Figure S4). As depicted in Figure 1I,J, LPS stimulation induced control AMs (MH‐S cells transfected with the negative control (NC) plasmid through lentiviral transduction) to secrete multiple pro‐inflammatory cytokines including TNF‐α and IL‐6, while BMAL1 overexpression significantly suppressed this inflammatory response. mRNA levels of *Tnf‐α* and *Il‐6* exhibited the same trend (Figure S5A,B). In addition, BMAL1 overexpression robustly suppressed LPS‐induced production of intracellular ROS in MH‐S cells, as detected by dihydroethidium (DHE) probes (Figure 1K,L; Figure S5C). Furthermore, the overexpression of BMAL1 notably impeded LPS‐induced M1 polarization of AMs, as evident by immunofluorescence (Figure 1M; Figure S5D) and flow cytometry (Figure 1N; Figure S5E) analyses of the characteristic M1 marker CD86. These results indicate that BMAL1 exerted significant effects in alleviating inflammatory responses, oxidative stress, and M1 polarization of AMs. Further investigation is needed to elucidate the specific mechanisms underlying these phenomena.

### 2.3 BMAL1 Overexpression Effectively Inhibits AM Glycolysis

**FIGURE 2:**
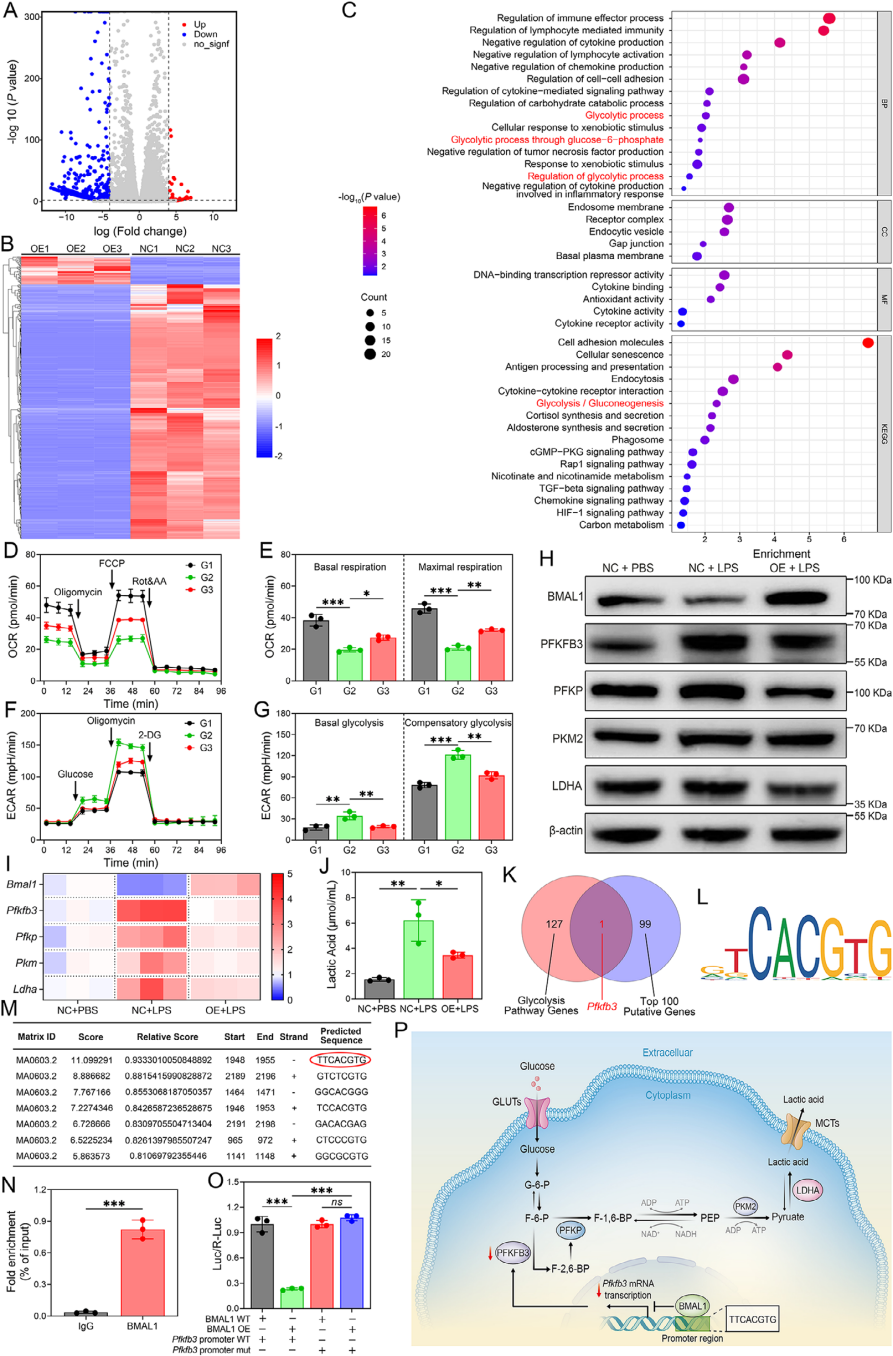
BMAL1 inhibits glycolysis by suppressing *Pfkfb3* transcription. (A,B) Volcano plot and heatmap (*n* = 3) of differentially expressed genes between BMAL1‐OE and NC MH‐S cells. Thresholds: |fold change| > 4 and *p* < 0.01. (C) GO (BP/CC/MF) and KEGG enrichment analysis of glycolysis‐related genes between BMAL1‐OE and NC MH‐S cells. (D,E) Mitochondrial basal respiration and maximal respiration parameters of MH‐S cells after different treatments, as determined by Seahorse XF Analyzer‐mediated OCR measurements (*n* = 3). **Treatments: G1**: NC + PBS (NC MH‐S cells treated with PBS); **G2**: NC + LPS (NC MH‐S cells treated with 1 µg·mL^−^^1^ of LPS for 24 h); **G3**: OE + LPS (BMAL1‐OE MH‐S cells treated with 1 µg·mL^−^^1^ of LPS for 24 h). (F, G) Basal glycolysis and compensatory glycolysis parameters of MH‐S cells after different treatments indicated in D, as determined by Seahorse XF Analyzer‐mediated ECAR measurement (*n* = 3). (H) Western blotting analysis of BMAL1 and key glycolytic enzymes (PFKFB3, PFKP, LDHA, and PKM2) in MH‐S cells following different treatments. (I) Heatmap of relative mRNA levels of *Bmal1*, *Pfkfb3*, *Pfkp*, *Pkm*, and *Ldha* in MH‐S cells after different treatments (*n* = 3). (J) Comparison of concentrations of lactic acid secreted from MH‐S cells after different treatments (*n* = 3). (K) Venn diagram showing overlap between glycolysis‐related genes and top 100 putative genes with BMAL1 ChIP‐seq peaks in promoter regions. (L,M) Putative BMAL1‐binding motif sequence and putative BMAL1‐binding sites in *Pfkfb3* promoter region using the Jaspar database. (N) Verification of the ability of BMAL1 binding to *Pfkfb3* promoter by CHIP‐qPCR assay (*n* = 3). (O) Dual‐luciferase reporter assay evaluating the transcriptional activity of the *Pfkfb3* promoter in response to BMAL1 modulation (*n* = 3). (P) Schematic diagram of the negative regulatory mechanism of BMAL1 in glycolysis. Quantitative data are presented as mean ± SD. Statistical significance was analysed using one‐way ANOVA with post‐hoc corrections. ^*^ *p* <0.05, ^**^ *p* <0.01, ^***^ *p* <0.001; ns: no significant difference.

Transcriptomic sequencing was performed on BMAL1‐OE and NC MH‐S cells. Differentially expressed genes between the two cell types were identified using thresholds of *p* <0.01 and |log_2_ fold change| > 4 (Figure 2A,B). Enrichment analyses of the Kyoto Encyclopedia of Genes and Genomes (KEGG) and Gene Ontology (GO) were conducted to interpret the data. The results indicated significant enrichment of glycolysis‐related pathways in both databases across all categories, including GO Biological Process (BP), Cellular Component (CC), and Molecular Function (MF) (Figure 2C).

We then examined the effect of BMAL1 on glycolytic activity in AMs. Seahorse extracellular flux (XF) analysis revealed that LPS stimulation not only suppressed mitochondrial respiration in AMs (NC MH‐S cells), as evidenced by decreased basal and maximal oxygen consumption rates (OCR), but also enhanced glycolytic function, including both basal glycolysis and glycolytic capacity, as indicated by extracellular acidification rate (ECAR) analysis (Figure 2D–G). Nevertheless, BMAL1 overexpression dramatically reversed these metabolic alterations. The expression of key glycolytic enzymes (PFKFB3, PFKP, LDHA, and PKM2) in AMs, which serve as functional markers for glycolysis, was markedly upregulated upon LPS stimulation (Figure 2H,I; Figure S6), concordant with the overall elevation in glycolytic activity. In contrast, their mRNA and protein expression levels in AMs were notably reduced when BMAL1 was overexpressed. We then measured extracellular lactic acid, a primary by‐product of glycolysis, in AMs after LPS challenge. As shown in Figure 2J, LPS treatment induced AMs to produce substantial amounts of lactic acid, whereas BMAL1 overexpression markedly suppressed this effect. These results indicate that upregulating BMAL1 potently suppress glycolysis in AMs, which might be a key mechanism for alleviating or reversing their M1 polarization, inflammatory responses, and oxidative stress.

### 2.4 BMAL1 Knockdown Aggravates AM Glycolysis and Lung Injury

Given the suppressive effect of BMAL1 overexpression on AM glycolysis, we hypothesized that BMAL1 deficiency would conversely exacerbate metabolic reprogramming and inflammatory responses. To test this hypothesis, we performed loss‐of‐function assays using a macrophage‐specific adeno‐associated virus (F4/80‐shBMAL1‐AAV). During in vitro experiments, we successfully transduced MH‐S cells with F4/80‐shBMAL1‐AAV and verified the significant reduction of BMAL1 mRNA levels via RT‐qPCR (Figure S7A). Following LPS stimulation, we conducted a series of assays examining glycolysis, inflammation, and ROS production (grouped as NC+LPS vs. KD+LPS). The result clearly demonstrates that, compared with the NC+LPS group, BMAL1 downregulation in MH‐S cells is entirely sufficient to significantly exacerbate glycolysis (Figure S7B–D), macrophage M1 polarization and the pro‐inflammatory responses (Figure S7E–H), and intracellular ROS generation (Figure S7I,J).

Furthermore, we extended this loss‐of‐function approach to the in vivo SA‐ARDS model. After intravenous administration of the macrophage‐specific shBMAL1‐AAV, we isolated AMs from the BALF and confirmed the effective in vivo knockdown of via RT‐qPCR (Figure S8A). As expected, mice with macrophage‐specific BMAL1 knockdown exhibited profoundly exacerbated lung injury (Figure S8B–D), characterized by aggravated histological damage scores (H&E), increased pulmonary edema (W/D ratio), massively amplified pro‐inflammatory cytokine release in the BALF (Figure S8E–G), and elevated ROS levels (Figure S8H,I) in lung cells. Collectively, these reverse‐verification results compellingly demonstrate that BMAL1 downregulation is fully sufficient to drive macrophage glycolytic hyperactivation and worsen SA‐ARDS progression.

### 2.5 BMAL1 Attenuates Glycolysis by Suppressing *Pfkfb3* Transcription

To elucidate the molecular mechanism by which BMAL1 regulates glycolysis, we analyzed the publicly available Gene Expression Omnibus (GEO) dataset GSM2522477, which contains chromatin immunoprecipitation sequencing (ChIP‐Seq) for BMAL1 in mouse macrophages. Using the Cistrome Data Browser, we retrieved the top 100 genes with the highest BMAL1 binding scores in the promoter region. After performing a Venn diagram intersection analysis with the glycolysis‐related gene set, *Pfkfb3* emerged as the sole intersecting gene (Figure 2K). UCSC Genome Browser confirmed that BMAL1 predominantly binds to the promoter region of the *Pfkfb3* gene (Figure S9). In addition, BMAL1 binding sites within the *Pfkfb3* promoter were predicted using the Jaspar database (Figure 2L,M). ChIP‐qPCR at the top‐ranked binding site confirmed significantly increased binding of BMAL1 to the predicted genomic region compared with the IgG control (Figure 2N). To validate these findings, we performed dual‐luciferase reporter assays using wild‐type and mutant *Pfkfb3* promoter plasmids were conducted and demonstrated that BMAL1 directly binds to the *Pfkfb3* promoter and negatively regulates its transcription (Figure 2O). These findings elucidate the mechanism by which the transcription factor BMAL1 regulates cellular glycolysis: it directly binds to the promoter region of *Pfkfb3* to repress its transcription, thereby reducing the production of PFKFB3 (a key glycolytic enzyme) and activation of the downstream glycolytic cascade (Figure 2P).

### 2.6 PFKFB3 is a Critical Initiator for Glycolysis in LPS‐Activated AMs

**FIGURE 3:**
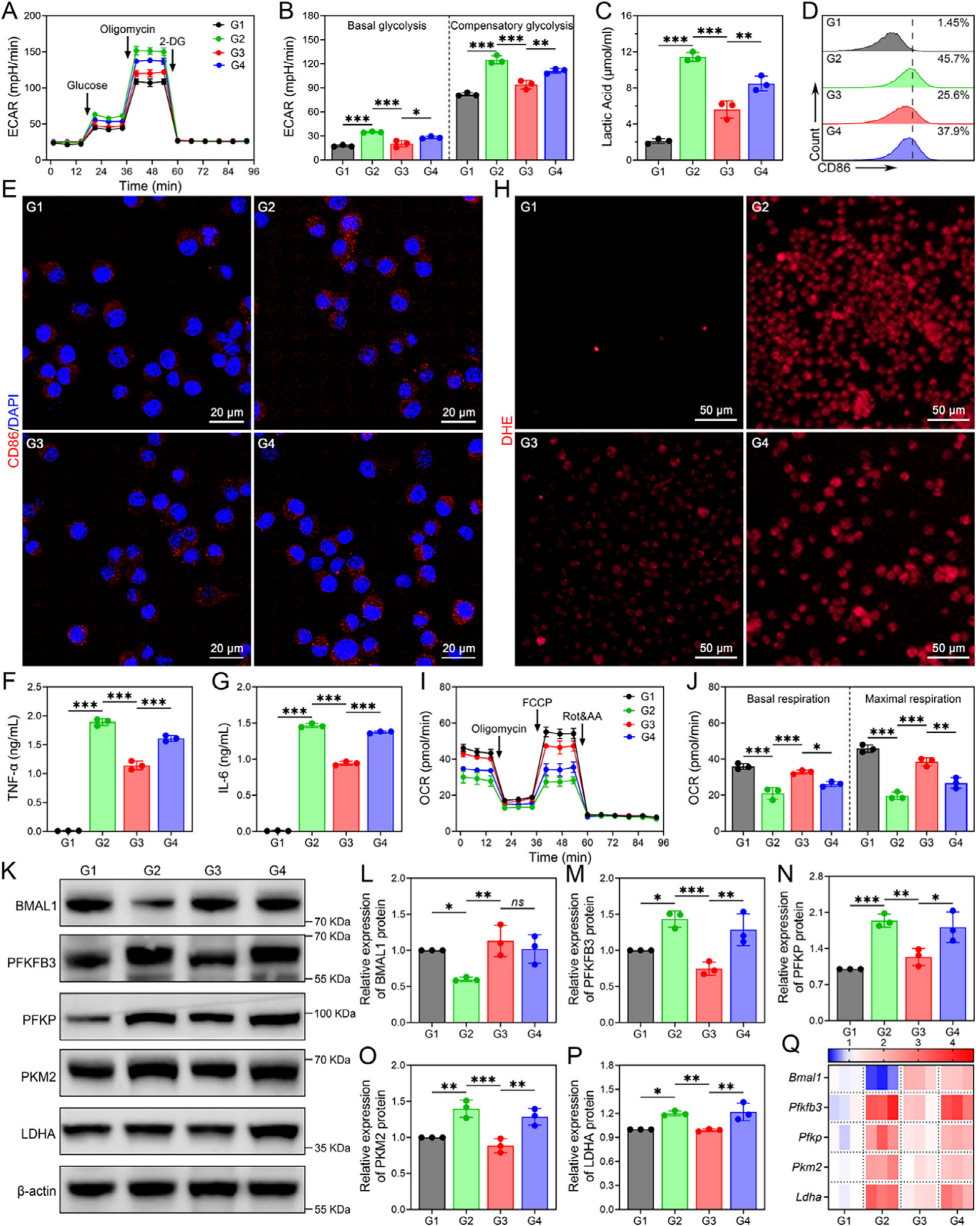
Assessment of whether PFKFB3 is a key initiator of glycolysis in LPS‐stimulated AMs. (A,B) Basal glycolysis and compensatory glycolysis parameters of MH‐S cells after different treatments, as determined by Seahorse XF Analyzer‐mediated ECAR measurement (*n* = 3). **Treatments**: **G1**: NC + PBS (NC MH‐S cells treated with PBS); **G2**: NC + LPS (NC MH‐S cells treated with 1 µg·mL^−^^1^ LPS for 24 h); **G3**: OE + LPS (BMAL1‐OE MH‐S cells treated with 1 µg·mL^−^^1^ LPS for 24 h); **G4**: OE + LPS + PFKFB3‐OE (BMAL1‐OE MH‐S cells transfected with the PFKFB3‐OE plasmid treated with 1 µg·mL^−^^1^ LPS for 24 h). (C) Comparison of lactic acid secretion levels of MH‐S cells following different treatments indicated in A (*n* = 3). (D,E) Flow cytometry analysis and Immunofluorescence assay for CD86 expression in MH‐S cells from different groups indicated in A. (F,G) Comparison of TNF‐α and IL‐6 secretion levels of MH‐S cells after different treatments indicated in A (*n* = 3). (H) Representative fluorescence microscopy images of DHE‐stained MH‐S cells after different treatments indicated in A. (I,J) Mitochondrial basal respiration and maximal respiration parameters in MH‐S cells after different treatments indicated in A, as determined by Seahorse XF Analyzer‐mediated OCR measurement (*n* = 3). (K–P) Western blotting and quantitative analysis of BMAL1, PFKFB3, PFKP, PKM2, and LDHA in MH‐S cells after different treatments indicated in A (*n* = 3). (Q) Heatmap of relative mRNA levels of *Bmal1*, *Pfkfb3*, *Pfkp*, *Pkm*, and *Ldha* in MH‐S cells after different treatments indicated in A (*n* = 3). Quantitative data are presented as mean ± SD. Statistical significance were analyzed using one‐way ANOVA with post‐hoc corrections. ^*^ *p* <0.05, ^**^ *p* <0.01, ^***^ *p* <0.001; ns: no significant difference.

To determine whether PFKFB3 is essential for initiating glycolysis, M1 polarization, and inflammatory responses in LPS‐activated AMs, we conducted rescue experiments. Specifically, we introduced a PFKFB3 overexpression (OE) plasmid into BMAL1‐OE MH‐S cells. As shown in Figure 3A–C, PFKFB3 overexpression resulted in a marked increase in glycolytic flux and lactic acid production in BMAL1‐OE MH‐S cells upon LPS stimulation, indicating an enhancement of glycolysis process. Moreover, overexpression of PFKFB3 promoted M1 polarization (Figure 3D,E,; Figure S10A,B), secretion of pro‐inflammatory cytokines TNF‐α and IL‐6 (Figure 3F,G; Figure S10C,D), and excessive production of ROS (Figure 3H; Figure S10E,F) of BMAL1‐OE MH‐S cells. Conversely, mitochondrial respiration, which had been restored by BMAL1 overexpression during LPS stimulation, was suppressed upon PFKFB3 overexpression (Figure 3I,J). Furthermore, PFKFB3‐OE overexpression directly upregulated downstream key glycolytic enzymes (PFKP, LDHA, and PKM2), both at the protein and mRNA levels (Figure 3K–Q). These results collectively demonstrated that PFKFB3 serves as an initiator of glycolysis in LPS‐activated AMs, which leads to subsequent AM M1 polarization and inflammatory responses. In addition, PFKFB3 overexpression reversed the protective effects of BMAL1 on LPS‐induced metabolic and inflammatory alterations.

### 2.7 Specificity of the BMAL1/PFKFB3 Regulatory Axis in Macrophage Glycolysis

Having established PFKFB3 as a primary initiator of glycolysis, we further investigated whether other upregulated glycolytic enzymes independently contribute to this process. We overexpressed PFKP, PKM2, and LDHA in LPS‐stimulated, BMAL1‐overexpressing (BMAL1‐OE) MH‐S cells. While PKM2 and LDHA overexpression showed weak rescuing effects, the overexpression of PFKP exhibited a robust rescue effect on glycolytic flux and inflammatory responses, highly comparable to that of PFKFB3 (Figure S11). This observation perfectly aligns with the canonical metabolic cascade, as the enzymatic product of PFKFB3 acts as a direct potent activator for PFKP. Therefore, these results indicate that PFKP functions as a downstream effector dependent on the initial activation of the PFKFB3 axis, rather than acting as a parallel independent node.

Furthermore, macrophage glycolytic reprogramming is also regulated by metabolic hubs such as HIF‐1α, mTOR, and c‐Myc. Our transcriptional screening revealed that BMAL1 overexpression in LPS‐stimulated MH‐S cells did not significantly alter the mRNA levels of *Mtor* or *Myc* (Figure S12A,B). Although a slight elevation in *Hif1a* expression was observed (Figure S12C), this finding functionally contradicts our observed phenotype. Since HIF‐1α is a potent activator of glycolysis, its upregulation would typically exacerbate glycolytic flux, whereas our data definitively show that BMAL1 robustly inhibits glycolytic hyperactivation. Consequently, these results suggest that the protective metabolic regulation of BMAL1 in SA‐ARDS is predominantly driven by the specific targeting of PFKFB3, acting independently of the HIF‐1α, mTOR, or c‐Myc signaling pathways.

### 2.8 Development of an Engineered Tetrahedral DNA Nanostructure for Precise Activation of BMAL1 in AMs

Building on the aforementioned findings, an advanced nanoplatform was designed to precisely and efficiently upregulate BMAL1 expression in AMs. This nano‐formulation, termed TNT, was prepared by using the tetrahedral DNA nanostructure (TDN) as a carrier to load the BMAL1 agonist Nobiletin (Nob, via intercalation into the center of double‐stranded DNA of TDN) and the AM‐targeting peptide Tuftsin (via electrostatic adsorption) (Scheme 1A). Incidentally, TDN was fabricated through the self‐assembly of 4 single‐stranded DNA (ssDNA) strands with specific sequences (Table S1).

**FIGURE 4:**
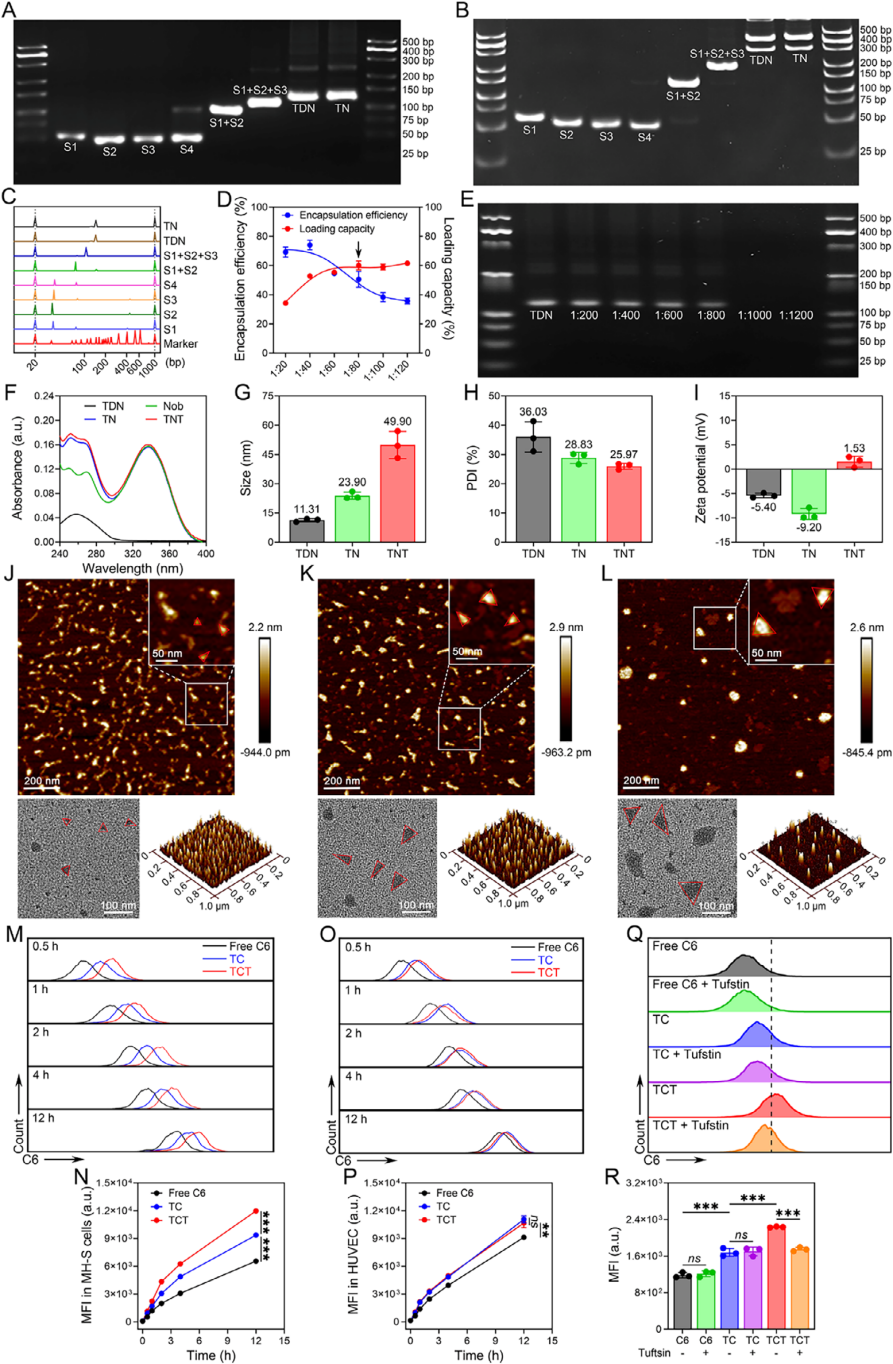
Synthesis and characterization of TNT. (A–C) Validation of successful synthesis of TDN and TN through AGE, PAGE, and CE. (D) Encapsulation efficiency and drug loading efficiency of Nob in TDN under different TDN‐to‐Nob ratios (*n* = 3). The arrow indicates optimal drug loading efficiency. (E) AGE of TDN‐Tuftsin complex under various TDN‐to‐Tuftsin ratios. (F) UV–vis absorption spectra of Nob, TDN, TN, and TNT in aqueous solution. (G–I) Hydrodynamic diameter, PDI, and zeta potential of TDN, TN, and TNT in ddH_2_O_2_ (*n* = 3). (J–L) AFM (top and bottom right) and TEM (bottom left) of TDN, TN, and TNT. (M–P) Flow cytometry analysis (for C6 fluorescence) and mean fluorescence intensity (MFI) quantification (*n* = 3) of MH‐S cells (M,N) or HUVECs (O,P) after incubation with free C6 (16 µm), TC (16 µm based on the loaded C6), or TCT (16 µm based on the loaded C6) for different times. (Q,R) Flow cytometry analysis (for C6 fluorescence) and MFI quantification (*n* = 3) of MH‐S cells that were pre‐treated with or without Tuftsin prior to incubation with free C6 (16 µm), TC (16 µm based on the loaded C6), or TCT (16 µm based on the loaded C6) for 2 h. Quantitative data are presented as mean ± SD. Statistical significance was analysed using one‐way ANOVA with post‐hoc corrections. ^**^ *p* <0.01, ^***^ *p* <0.001; *ns*: no significant difference.

The successful assembly of TDN was verified employing three electrophoretic techniques: agarose gel electrophoresis (AGE), polyacrylamide gel electrophoresis (PAGE), and capillary electrophoresis (CE) (Figure 4A–C). Additionally, TN (TDN loaded with Nob) exhibited similar bands to TDN. The encapsulation efficiency and drug loading capacity were assessed by measuring the absorbance at 334 nm (Nob's absorption peak) under different TDN‐to‐Nob ratios. As illustrated in Figure 4D, the optimal drug loading efficiency was achieved at a 1:80 ratio, with an encapsulation efficiency of 50.56% ± 5.42% and a drug loading rate of 60.00% ± 3.04%. Subsequently, AGE was performed to identify the optimal TDN‐to‐Tuftsin ratio. A ratio of 1:1000 was found to be optimal, as evidenced by the complete disappearance of the bands (negatively charged TDN became electrically neutral or positively charged upon incorporation of Tuftsin), indicating full binding of Tuftsin to TDN (Figure 4E). UV–vis absorbance spectra of TDN, Nob, TN, and TNT were then characterized, further confirming the successful synthesis of TN, and TNT (Figure 4F). Notably, the absorbance of TN and TNT was similar to that of Nob, confirming the successful loading of Nob onto TDN. To further evaluate the physicochemical properties of the nano‐formulation, dynamic light scattering (DLS) analysis was performed. As shown in Figure 4G,H, the hydrated sizes of TDN, TN, and TNT were measured to be 11.31 ± 0.79 nm, 23.90 ± 1.83 nm, and 49.90 ± 6.91 nm, respectively, with all samples exhibiting normal polydispersity index (PDI) values. The surface potentials of the three nanoparticles were measured to be −5.40 ± 0.46 mV, −9.20 ± 1.13 mV, and 1.53 ± 1.12 mV, respectively (Figure 4I). Of note, the addition of positively charged Tuftsin onto TN reversed its negative charge. Furthermore, atomic force microscopy (AFM) and transmission electron microscopy (TEM) imaging clearly revealed that TDN, TN, and TNT all exhibited typical tetrahedral structures, with dimensions ranging from 10 to 50 nm (Figure 4J–L). These results confirm the successful synthesis of TNT, which demonstrates potential for targeting AMs and the precise activation of BMAL1 in AMs.

### 2.9 Assessment of TNT's Targeting Capability for AMs In Vitro

The targeting efficiency of TNT toward AMs was comprehensively evaluated in vitro using Coumarin 6 (C6) as a fluorescent model drug to simulate Nob, resulting in the formation of TC (C6‐loaded TDN) and TCT (C6‐loaded TDN conjugated with Tuftsin). As shown in Figure 4M,N and Figure S13, the intracellular green fluorescence of C6 in MH‐S cells increased progressively over time when incubated with free C6, TC, or TCT. Specifically, the cellular uptake of TC by AMs was significantly higher than that of free C6, indicating that the TDN structure possesses an inherent ability to enhance drug delivery into cells. Following conjugation with Tuftsin to form TCT, the cellular uptake by AMs reached the highest level, suggesting that the targeting ability of Tuftsin toward AMs enhanced drug internalization. We then included a control cell line (HUVEC) to validate the specificity of Tuftsin targeting AMs. As shown in Figure 4O,P, both cell types demonstrated higher uptake efficiency for TC than for free C6. Nevertheless, unlike in MH‐S cells, no obvious difference in uptake between TC and TCT was observed in HUVECs, confirming the targeting specificity of Tuftsin for AMs. To further explore the role of Tuftsin, MH‐S cells were pre‐treated with Tuftsin prior to drug administration to block the corresponding receptors. As shown by flow cytometry, pre‐treatment with Tuftsin did not affect the efficiency of MH‐S cells in internalizing free C6 and TC, but apparently reduced their ability to uptake TCT (Figure 4Q,R), providing strong evidence for Tuftsin's specific targeting toward AMs.

### 2.10 Therapeutic Efficacy of TNT in In Vitro SA‐ARDS Model

**FIGURE 5:**
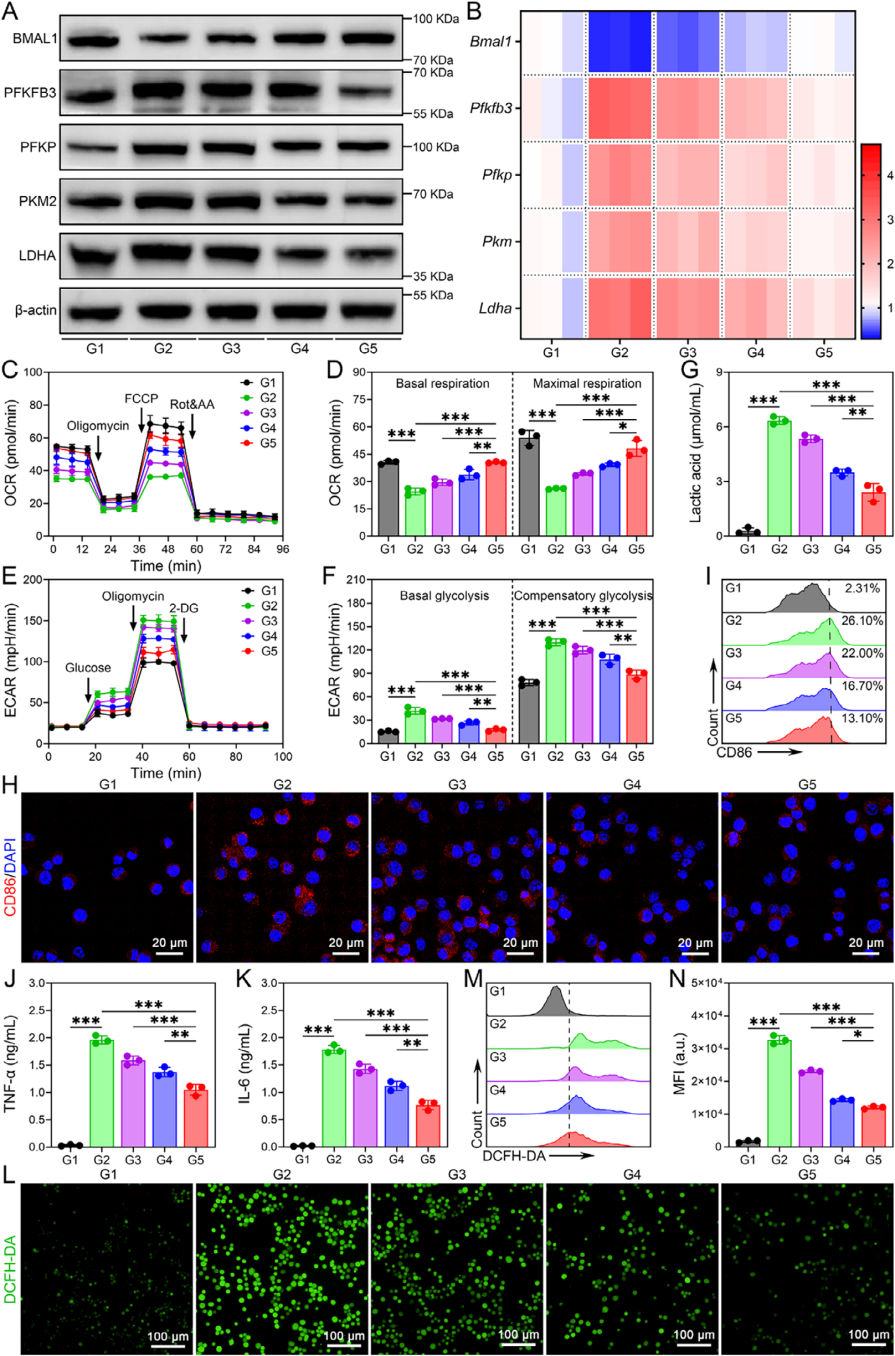
In vitro therapeutic efficacy of TNT in LPS‐stimulated AMs. (A) Western blotting analysis of BMAL1, PFKFB3, PFKP, PKM2, and LDHA in MH‐S cells following different treatments. **Treatments**: **G1**: MH‐S cells treated with PBS for 24 h; **G2**: MH‐S cells treated with LPS (1 µg·mL^−^^1^) for 24 h; **G3**: MH‐S cells treated with LPS (1 µg·mL^−^^1^) and Nob (16 μμ) for 24 h; **G4**: MH‐S cells treated with LPS (1 µg·mL^−^^1^) and TN (16 μΜ based on Nob) for 24 h; **G5**: MH‐S cells treated with LPS (1 µg·mL^−^^1^) and TNT (16 μΜ based on Nob) for 24 h. (B) Heatmap of relative mRNA levels of *Bmal1*, *Pfkfb3*, *Pfkp*, *Pkm*, *Ldha* in MH‐S cells after different treatments indicated in A (*n* = 3). (C,D) Mitochondrial basal respiration and maximal respiration parameters in MH‐S cells after different treatments, as determined by Seahorse XF Analyzer‐mediated OCR measurement (*n* = 3). (E,F) Basal glycolysis and compensatory glycolysis parameters of MH‐S cells after different treatments, as determined by Seahorse XF Analyzer‐mediated ECAR measurement (*n* = 3). (G) Comparison of lactic acid secretion in MH‐S cells after different treatments (*n* = 3). (H) Immunofluorescence staining for CD86 in MH‐S cells after different treatments. (I) Flow cytometry analysis of CD86 expression on MH‐S cells after different treatments. (J,K) Quantification of TNF‐α and IL‐6 production from MH‐S cells after different treatments (*n* = 3). (L–N) DCFH‐DA (an intracellular ROS probe) staining analysis, flow cytometry analysis of DCFH‐DA intensity and DCFH‐DA MFI statistics (*n* = 3) of differently treated MH‐S cells. Quantitative data are presented as mean ± SD. Statistical significance was calculated via ordinary one‐way ANOVA. Significance levels: ^*^ *p* <0.05, ^**^ *p* <0.01, ^***^ *p* <0.001.

The biological effects of TNT were investigated in vitro using LPS‐stimulated MH‐S cells to mimic the state of AMs in SA‐ARDS. Following LPS stimulation, both the protein and mRNA levels of BMAL1 in MH‐S cells were notably downregulated (Figure 5A,B; Figure S14A), consistent with previous findings. By contrast, treatment with Nob, TN, and TNT restored BMAL1 expression to varying degrees, with TNT exhibiting the most potent effect. Furthermore, LPS stimulation caused a decrease in mitochondrial respiration in MH‐S cells, manifested as reductions in both basal and maximal OCR (Figure 5C,D). Conversely, cellular glycolytic activity was enhanced, indicated by increased basal and compensatory glycolysis (Figure 5E,F) as well as lactic acid overproduction (Figure 5G). Treatment with Nob, TN, or TNT reversed these effects, with the most notable suppression of glycolytic activity and lactic acid levels occurring in the TNT group. Consistently, the expression of glycolysis‐related enzymes (PFKFB3, PFKP, LDHA, and PKM2) reflected the aforementioned metabolic changes and exhibited similar trends (Figure 5A,B,; Figure S14B–E).

After LPS activation, the proportion of M1‐phenotype AMs (Figure 5H; Figure S14F,G), generation of pro‐inflammatory cytokines (TNF‐α and IL‐6) (Figure 5J,K; Figure S14H,I) and ROS (Figure 5L–N) were significantly enhanced, Treatment with Nob, TN, or TNT alleviated these phenomena. More importantly, TNT consistently exhibited the best mitigation effects. To definitively conclusively confirm that these therapeutic benefits are intrinsically driven by the BMAL1 pathway, we evaluated the efficacy of TNT in BMAL1‐knockdown MH‐S cells transduced with F4/80‐shBMAL1‐AAV. Strikingly, BMAL1 deficiency abolished the TNT‐mediated suppression of macrophage glycolysis, as evidenced by a significant rebound in the ECAR and increased lactate production (Figure S15C). Furthermore, the capacity of TNT to inhibit M1 polarization and mitigate oxidative stress was substantially blunted. This loss of efficacy was demonstrated by the restored expression of the M1 marker CD86 (Figure S15D,E), increased secretion of pro‐inflammatory cytokines (TNF‐α and IL‐6) (Figure S15F,G), and elevated intracellular ROS levels (Figure S15H,I). Collectively, these in vitro results explicitly confirm that the anti‐glycolytic, anti‐inflammatory, and antioxidant efficacies of TNT are highly dependent on the activation of the BMAL1 pathway.

Furthermore, while suppressing M1‐driven inflammation is crucial, the reprogramming of macrophages toward an anti‐inflammatory M2 phenotype is equally crucial for the resolution of inflammation and subsequent tissue repair in SA‐ARDS. Given that both Nob and TDNs have been previously reported to exhibit M2‐promoting potential, we subsequently evaluated the effect of TNT on M2 polarization. As shown in Figure S16A–E, TNT treatment effectively induced this phenotypic switch in LPS‐challenged MH‐S cells, evidenced by the significant upregulation of the surface marker CD206, increased transcription of *Arg1* and *Mrc1*, and enhanced secretion of the anti‐inflammatory cytokine IL‐10. Functionally, these actively reprogrammed macrophages displayed robust capacities for tissue repair and debris clearance. In a Transwell co‐culture system, TNT‐treated macrophages significantly accelerated the wound healing of MLE‐12 alveolar epithelial cells, consistent with their elevated secretion of the repair factor TGF‐β1 (Figure S16F–H). Moreover, they demonstrated notably enhanced efferocytosis capacity for clearing apoptotic cells (Figure S16I–K), which functionally correlated with the targeted upregulation of *Mertk* (Figure S16D). Collectively, these findings suggest that TNT confers comprehensive cellular protection by simultaneously attenuating M1‐mediated inflammatory damage via the BMAL1/glycolysis axis and actively fostering a pro‐resolving microenvironment through M2 polarization.

### 2.11 Development and Characterization of RM@TNT: For In Vivo Application

**FIGURE 6:**
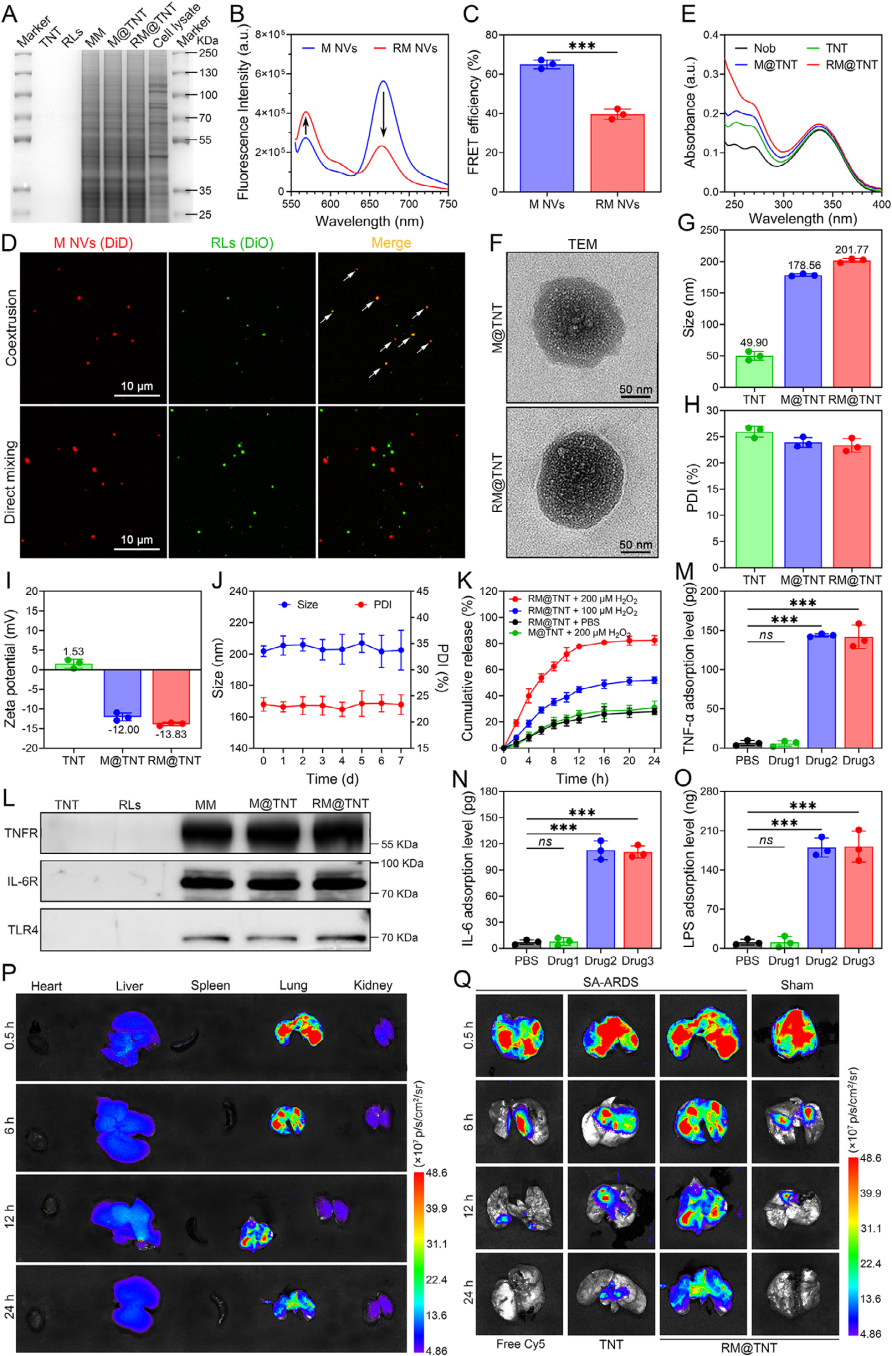
Preparation and characterization of RM@TNT. (A) SDS–PAGE analysis of protein components of TNT, RLs, MH‐S cell membrane (MM), M@TNT, RM@TNT, and MH‐S cell lysate. Samples were run at the same protein concentrations (1 mg·mL^−^^1^) except for the TNT and RLs. (B) Emission spectra of M NVs (labeled with both DiI and DiD) and RM NVs (DiI‐ and DiD‐labeled M NVs fused with unlabeled RLs) (Excitation wavelength: 549 nm). Arrowheads indicate fluorescence recovery of DiI (peaked at 565 nm) and attenuation of DiD (peaked at 663 nm), suggesting the disruption of FRET. (C) Quantification of FRET efficiency of M NVs and RM NVs indicated in B (*n* = 3). (D) Representative confocal images of DiD‐labeled M NVs (red) fused with DiO‐labeled RLs (green) after coextrusion through a 200 nm polycarbonate filter. Arrows indicate the resultant RM NVs after successful membrane fusion. M NVs directly mixing with RLs was set as control. (E) UV–vis spectra of Nob, TNT, M@TNT, and RM@TNT in aqueous solution. (F) Typical TEM images of a M@TNT and a RM@TNT. (G–I) Hydrodynamic diameter, PDI, and zeta potential comparison among TNT, M@TNT and RM@TNT (*n* = 3). (J) Stability assessment of RM@TNT in PBS at 4°C for 7 days by monitoring changes in hydrodynamic diameter and PDI (*n* = 3). (K) Cumulative TNT release profiles from M@TNT or RM@TNT after incubation in PBS or different concentrations of H_2_O_2_ (*n* = 3). (L) Western blotting analysis for IL‐6R, TNFR, and TLR4 in TNT, RL, MM, M@TNT, and RM@TNT. Samples were run at the same protein concentrations (2 mg·mL^−^^1^) except for the TNT and RLs. (M–O) Adsorption efficiency of TNT (Drug1, 16 µm based on Nob), M@TNT (Drug2, 16 µm based on Nob), and RM@TNT (Drug3, 16 µm based on Nob) toward IL‐6 (500 pg), TNF‐α (500 pg) and LPS (300 ng) following a 2 h co‐incubation at 37°C (*n* = 3). (P) Time‐lapse IVIS imaging of various organs isolated from SA‐ARDS mice after intranasal administration of Cy5‐labeled RM@TNT (10 mg·kg^−^^1^ based on Nob). (Q) Time‐lapse IVIS imaging of lungs isolated from SA‐ARDS or sham mice after intranasal administration of Free Cy5, Cy5‐labeled TNT, or Cy5‐labeled RM@TNT (10 mg·kg^−^^1^ based on Nob). Quantitative data are presented as mean ± SD. Statistical significance was calculated via ordinary one‐way ANOVA. Significance levels: ^***^ *p* <0.001; *ns*: no significant difference.

To improve the in vivo bioavailability and targeting efficiency to pulmonary inflammatory foci of TNT, AM cell membrane biomimetic nanovesicles (M NVs) were employed to encapsulate it (forming M@TNT). Specifically, M NVs are highly biocompatible and carry multiple chemokine receptors from AM membranes [38, 39]. Furthermore, given that ROS are predominantly generated in SA‐ARDS‐affected lung regions, we incorporated ROS‐responsive liposomes (RLs) into M NVs to construct a hybrid “shell” (RM NVs) for TNT loading, resulting in the final formulation RM@TNT (Scheme 1A). This design was designed to enable spatiotemporally controlled release of the therapeutic payload (TNT) specifically at the site of inflammation (Scheme 1B). Sodium dodecyl sulfate‐polyacrylamide gel electrophoresis (SDS‐PAGE) analysis confirmed that the protein profiles of RM@TNT and M@TNT closely matched those of AM membrane (MM), but differed from those of MH‐S whole cell lysates containing intracellular proteins (Figure 6A), indicating that only pure AM cell membrane components were used for TNT encapsulation. To verify the successful fusion between M NVs and RLs, a fluorescence resonance energy transfer (FRET) assay was employed using the lipid dyes 1,1'‐dioctadecyl‐3,3,3',3'‐tetramethylindocarbocyanine perchlorate (DiI) and 1,1'‐dioctadecyl‐3,3',3'‐tetramethylindodicarboximide perchlorate (DiD) as a pair of FRET dyes. In principle, M NVs were llabeled with both DiI and DiD. When the FRET phenomenon occurs, the fluorescence emitted by DiI (with an emission peak at ∼565 nm) can be absorbed by DiD, leading to an increase in the fluorescence intensity of DiD (peak ∼663 nm) and a concomitant decrease in that of DiI. However, upon successful fusion of unlabeled RLs with M NVs (forming RM NVs), the FRET efficiency was disrupted, leading to enhanced fluorescence at 565 nm and a corresponding reduction at 663 nm (Figure 6B,C). To visualize membrane fusion, M NVs and RLs were stained with DiD and 1,1'‐dioctadecyl‐3,3'‐tetramethylindocarbocyanine perchlorate (DiO), respectively, and then observed using confocal fluorescence microscopy. As shown in Figure 6D, coextrusion through a 200 nm filter resulted in substantial fusion between DiD‐labeled M NVs and DiO‐labeled RLs, confirming the successful formation of RM@TNT. In contrast, direct mixing without coextrusion failed to induce membrane fusion [40]. The UV–vis absorbance spectra of M@TNT and RM@TNV were similar to those observed for Nob and TNT (Figure 6E), indicating the successful loading of TNT into M NVs and RM NVs, respectively, and the effective fabrication of both M@TNT and RM@TNT. TEM imaging revealed that both the M@TNT and RM@TNT formulations displayed typical vesicular structures with diameters of approximately 170 nm (Figure 6F). The hydrodynamic particle sizes of M@TNT and RM@TNT, as determined by dynamic light scattering (DLS), were measured to be 178.56 ± 2.00 nm and 201.77 ± 3.33 nm, respectively (Figure 6G), with PDI values falling within the acceptable range (Figure 6H). Following encapsulation into M NVs or RM NVs, the surface potential of TNT shifted from positive (1.53 ± 1.12 mV) to negative values (−12.00 ± 1.01 mV and −13.83 ± 0.42 mV, respectively) (Figure 6I), indicating an enhancement in both the biocompatibility and stability of the formed M@TNT and RM@TNT. Specifically, TNT possesses a cationic surface that promotes non‐specific cellular adsorption through electrostatic interactions [41, 42]. In contrast, NV encapsulation confers an anionic charge, which minimizes non‐targeted binding and thereby indicating selective accumulation at SA‐ARDS lesions.

The storage stability of RM@TNT was then evaluated. After storage in PBS at 4°C for 7 d, no significant changes in particle size or PDI were detected (Figure 6J). To assess the ROS‐responsive drug release of RM@TNT, hydrogen peroxide (H_2_O_2_) was used to simulate the inflamed microenvironment of SA‐ARDS foci. As shown in Figure 6K, RM@TNT detected a sustained release of TNT in PBS, reaching a maximum release rate of 27.92% ± 2.33% at 24 h. However, the presence of H_2_O_2_ markedly accelerated drug release, with the cumulative release increasing to 52.04% ± 2.45% and 82.46% ± 3.57% at H_2_O_2_ concentrations of 100 and 200 µm, respectively. In contrast, M@TNT displayed no obvious acceleration of cargo release under a high‐concentration H_2_O_2_ environment_._

We further confirmed this ROS‐responsive release profile and structural stability in more physiologically relevant environments using Simulated Lung Fluid (SLF) and 10% FBS. As demonstrated in Figure S17A, RM@TNT retained its sensitive ROS‐responsive drug release capability in both SLF and 10% FBS, with release profiles highly consistent with those observed in PBS. Furthermore, RM@TNT displayed excellent colloidal stability throughout a 24‐h incubation period in SLF and 10% FBS, with minimal changes in hydrodynamic diameter and PDI (Figure S17B,C). These findings collectively confirm that RM@TNT possesses robust structural integrity and maintains its precise ROS‐triggered therapeutic delivery even within complex, protein‐rich biological fluids.

Macrophages inherently express various pro‐inflammatory cytokine receptors (such as TNFR and IL‐6R) and toll‐like receptors (TLRs, including TLR4) on their membrane surface [43, 44, 45]. Thus, biomimetic nanoparticles derived from macrophage membranes can serveas a decoy to adsorb and neutralize multiple pro‐inflammatory cytokines and LPS through receptor‐ligand specific binding, showing promise for treating sepsis [46]. Western blotting analysis confirmed that compared to TNT and RLs, M@TNT and RM@TNT retained TNFR, IL‐6R, and TLR4gaide, which originate from the cell membranes of AMs (Figure 6L). Moreover, unlike TNT which lacks adsorption properties, M@TNT and RM@TNT can directly and efficiently adsorb TNF‐α, IL‐6, and LPS in vitro, confirming their ability to neutralize inflammatory cytokines and endotoxins (Figure 6M–O).

### 2.12 In Vivo Pulmonary Retention and Macrophage‐Targeting of RM@TNT

We subsequently examined the residence time of RM@TNT in the lungs of SA‐ARDS mice and its ability to target pulmonary inflammatory foci. As shown in Figure 6P, following intranasal administration of Cy5‐labeled RM@TNT to SA‐ARDS mice, these nanoparticles preferentially accumulated in the lungs, with minimal accumulation detected in the liver and kidneys, and persisted in pulmonary tissues for over 24 h. In contrast, free Cy5 and TNT exhibited markedly shorter pulmonary residence times (Figure 6Q; Figure S18), highlighting the enhanced lung retention capability of RM@TNT. More importantly, RM@TNT persisted for extended periods only in the lung tissue of SA‐ARDS mice, whereas being rapidly cleared from the lungs of healthy mice (Figure 6Q; Figure S18). These results indicate that its inflammation‐targeting capability facilitates prolonged retention at pulmonary inflammatory sites.

To further investigate the cellular‐level distribution within the complex pulmonary microenvironment, we utilized a multi‐color flow cytometry gating strategy to identify different lung cell populations from single‐cell suspensions (Figure S19). The in vivo results revealed a highly specific targeted uptake profile: AMs exhibited a remarkably high Cy5‐positive uptake rate of 20.53% ± 0.95% (Figure S19A,B). In marked contrast, the uptake rates in other recruited immune cells, such as neutrophils (Neu) and monocytes (Mono), remained extremely low at approximately 1%. Furthermore, non‐professional phagocytes, including endothelial cells (EC) and epithelial cells (Epi), showed negligible background uptake (<1%). These quantitative single‐cell data explicitly demonstrate the exceptional and exclusive targeting specificity of RM@TNT toward AMs, ensuring precise delivery of its therapeutic cargo to the key cellular mediators of SA‐ARDS.

### 2.13 In Vivo Therapeutic Efficacy of RM@TNT for SA‐ARDS

**FIGURE 7:**
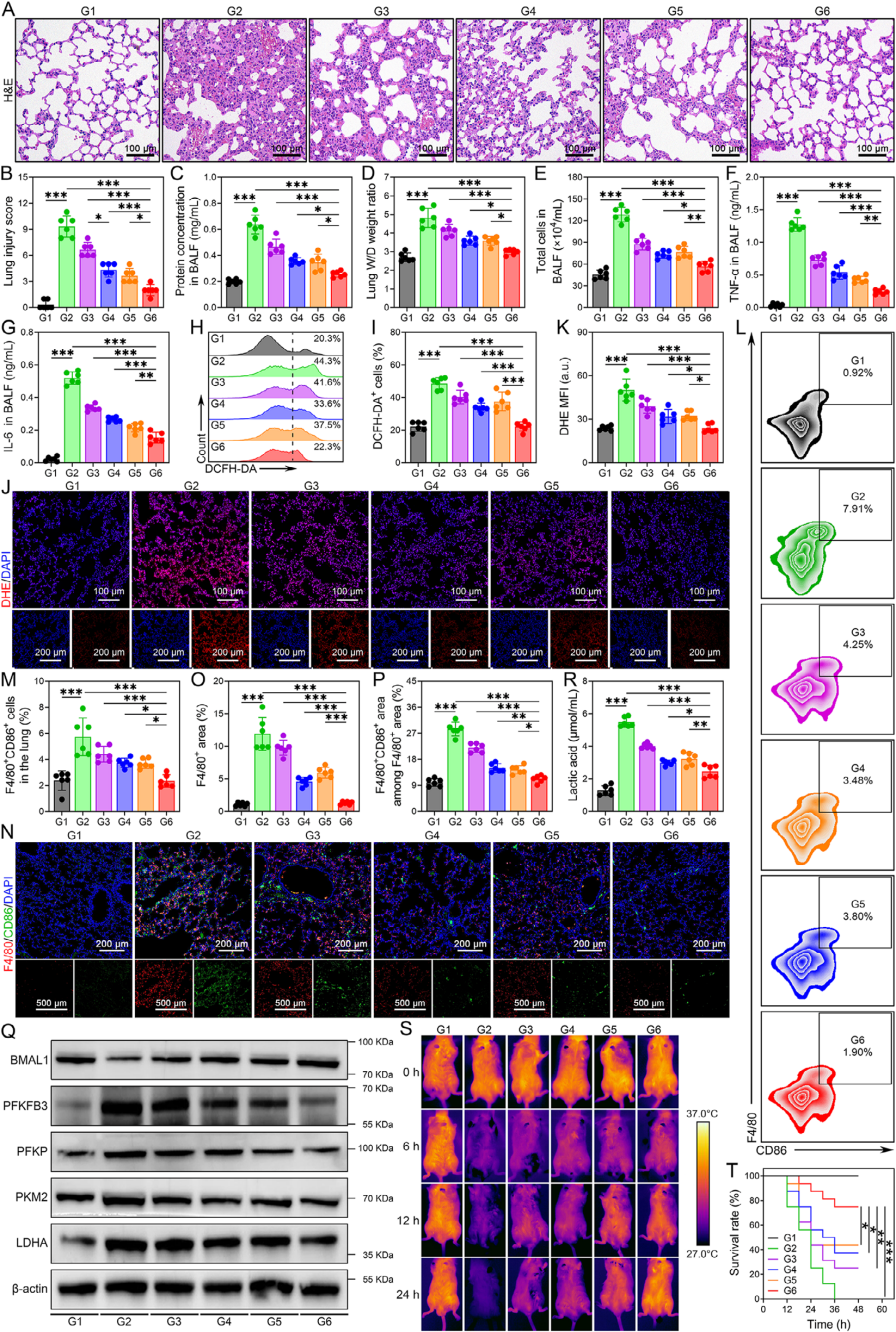
Therapeutic efficacy of RM@TNT for SA‐ARDS in mouse models. (A) H&E staining analysis of lung tissues of mice 24 h after different treatments. **Treatments**: The control group underwent laparotomy with immediate closure (the sham group, **G1**), while the remaining groups were subjected to standard CLP to induce SA‐ARDS. Thirty minutes after CLP, SA‐ARDS mice were intranasally administrated with 40 µL of saline (**G2**), Nob (10 mg·kg^−^^1^) (**G3**), TNT (10 mg·kg^−^^1^ based on Nob) (**G4**), M@TNT (10 mg·kg^−^^1^ based on Nob) (**G5**), or RM@TNT (10 mg·kg^−^^1^ based on Nob) (**G6**), respectively. (B–G) Quantification of lung injury scores (B), protein concentrations in BALF (C), pulmonary wet to dry (W/D) weight ratios (D), total cell counts in BALF (E), levels of TNF‐α (F) and IL‐6 (G) in BALF of mice after different treatments indicated in A (*n* = 6). (J,K) DHE (an intracellular ROS probe) staining analysis and DHE MFI statistics (*n* = 6) of lung tissues from differently treated mice. (L,M) Flow cytometry analysis and quantification of the percentage of F4/80^+^CD86^+^ cells (*n* = 6) in pulmonary single‐cell suspensions prepared by lung tissue homogenization of different groups of mice. (N) Immunofluorescence staining (for F4/80 and CD86) of lung tissues of mice after different treatments. (O,P) Quantification of the proportion of F4/80^+^ area in lung tissues and the ratio of F4/80^+^CD86^+^ area within F4/80^+^ area from different groups indicated in N (*n* = 6). (Q) Western blotting analysis of BMAL1, PFKFB3, PFKP, PKM2, and LDHA in lungs of different treated mice. (R) Comparison of lactic acid levels in BALF of mice after different treatments (*n* = 6). (S,T) Near‐infrared body temperature monitoring and survival rate statistics (*n* = 16) of different treated mice. Quantitative data are presented as mean ± SD. Statistical significance was calculated via ordinary one‐way ANOVA or log‐rank (Mantel‐Cox) test. Significance levels: ^*^ *p* <0.05, ^**^ *p* <0.01, ^***^ *p* <0.001.

To assess the therapeutic efficacy of RM@TNT in SA‐ARDS, mice were randomly divided into six groups. The control group underwent laparotomy with immediate closure (the sham group, G1), while the remaining groups were subjected to standard CLP to induce SA‐ARDS. Thirty minutes after CLP, mice in different groups were administered intranasally with saline (G2), Nob (G3), TNT (G4), M@TNT (G5), or RM@TNT (G6), respectively. After 24 h, lung tissues and bronchoalveolar lavage fluid (BALF) samples were collected for analysis. Histological examination of hematoxylin‐eosin (H&E)‐stained lung sections revealed substantial lung injury in the saline group (G2), characterized by alveolar wall thickening, increased secretions, extensive inflammatory cell infiltration, hemorrhage, and the formation of hyaline membranes (Figure 7A), confirming the successful establishment of the SA‐ARDS mouse model. Following the introduction of Nob (G3), these pathological manifestations were alleviated to a certain extent (Figure 7A), and the lung injury scores decreased accordingly (Figure 7B), further confirming the potential efficacy of BAML1 agonist in the treatment of SA‐ARDS in vivo. When Nob was loaded into the TDN carrier and modified with the AM‐targeting peptide Tuftsin to form TNT (G4), its therapeutic efficacy was obviously enhanced, indicating that precise targeting toward AMs can effectively enhance the efficacy of Nob. Incidentally, the inherent ROS‐scavenging activity of TDN may also contribute to this enhanced therapeutic effect. These findings suggested that encapsulating TNT within AM membrane‐derived nanovesicles (M NVs) (G5) could extend its retention time in the lungs of SA‐ARDS mice (Figure 6P,Q) and achieved broad‐spectrum clearance of multiple pro‐inflammatory cytokines and endotoxins (Figure 6M–O), thereby enhancing the therapeutic efficacy of TNT. However, only incorporating ROS‐responsive liposomes into M NVs (G6) remarkably improved its therapeutic efficacy (Figure 7A,B), demonstrating the advantages of a precise spatiotemporally drug release and delivery strategy.

SA‐ARDS mice (G2) displayed severe pulmonary barrier damage and edema, manifested as elevated total protein concentration in BALF (Figure 7C) and increased lung wet‐to‐dry (W/D) weight ratio (Figure 7D). Treatment with the various agents reduced these values to varying extents. The RM@TNT group (G6) exhibited the most pronounced reduction, with their levels approaching those of the sham control group (G1), demonstrating its superior capacity to reverse lung barrier dysfunction. Furthermore, compared to Nob, TNT, and M@TNT, RM@TNT treatment most effectively reduce the total cell count (predominantly inflammatory cells) (Figure 7E) and levels of the pro‐inflammatory cytokines TNF‐α and IL‐6 (Figure 7F,G) in BALF. Such anti‐inflammatory effect was further confirmed at the transcriptional level, with RM@TNT showing the most effective reduction in the expression of *Tnf‐α* and *Il‐6* in the lung tissues (Figure S20A,B). Oxidative stress levels are another critical indicator of the severity of lung damage during SA‐ARDS [47], with the concentration of ROS directly reflecting its intensity. Flow cytometric analysis of single‐cell suspensions derived from lung tissue and stained with DCFH‐DA (an indicator of intracellular ROS) revealed that the ROS levels in the lungs of SA‐ARDS mice (G2) were dramatically elevated compared to the sham group (G1) (Figure 7H,I). Nob, TNT, or M@TNT treatment moderately reduced oxidative stress levels, whereas treatment with RM@TNT effectively alleviated the production of ROS within pulmonary cells. DHE (another intracellular ROS indicator)‐stained lung tissue sections exhibited a similar trend (Figure 7J,K).

Macrophage M1 polarization is a key factor driving excessive inflammation and oxidative stress [48, 49]. Therefore, we assessed macrophage M1 polarization in the lung tissues of SA‐ARDS mice by quantifying the expression of related biomarkers. As depicted by flow cytometry, a notable increase in the proportion of F4/80^+^CD86^+^ cells (indicating M1 macrophages) in lungs (Figure 7L,M) was observed in SA‐ARDS mice (G2). Treatments with Nob, TNT, or M@TNT moderately reduced the percentage of M1 macrophages, while RM@TNT treatment produced the greatest reduction. To further visualize the quantity and distribution of macrophages and M1 macrophages in lung tissue, immunofluorescence was performed. As displayed in Figure 7N,O, compared with the sham group, the number of F4/80^+^ cells (representing all macrophages) in the alveolar region of SA‐ARDS mice (G2) was obviously increased, indicating enhanced macrophage infiltration. Moreover, the proportion of F4/80^+^CD86^+^ cells among F4/80^+^ cells also increased (Figure 7P,R), signifying enhanced M1 polarization of macrophages. Treatment with Nob, TNT, M@TNT, or RM@TNT reduced the proportion of total macrophages, particularly the pro‐inflammatory M1 subset, within the lungs, with the most pronounced reduction observed in the RM@TNT group (G6).

Complementing the suppression of M1 polarization, RM@TNT treatment effectively induced an in vivo phenotypic switch toward a pro‐resolving M2 state. Flow cytometric analysis demonstrated a significant expansion of the F4/80^+^CD206^+^ macrophage population in lung tissues following RM@TNT administration (Figure S21A–C). This shift was further corroborated by the upregulated pulmonary expression of M2‐specific markers (*Arg1* and *Mrc1*) and the efferocytosis receptor Mertk (Figure S21D). Consistent with these transcriptional changes, the levels of anti‐inflammatory and repair‐related factors (IL‐10 and TGF‐β1) in the BALF were markedly elevated in the RM@TNT group (Figure S21E,F). These findings demonstrate that RM@TNT facilitates pulmonary repair by actively fostering an immunoresolving M2 microenvironment, paralleling the therapeutic trends observed in vitro.

RM@TNT treatment significantly reduced BMAL1 expression in the lungs of SA‐ARDS mice, as demonstrated by RT‐qPCR and western blotting analyses, which showed marked downregulation at both the mRNA (Figure 7S) and protein (Figure 7Q; Figure S20C) levels. This finding was consistent with our in vitro data (Figure 5A,B). The introduction of Nob, TNT, or M@TNT partially upregulated BMAL1 expression in the lungs, which is attributed to the action of the BMAL1 agonist Nob. Notably, RM@TNT produced the most obvious upregulation.

Glycolysis is an important mechanism promoting M1 polarization of macrophages, as well as a major contributor to excessive inflammatory responses and oxidative stress. Upregulating BMAL1 effectively suppresses glycolysis in macrophages. (Figure 2). Therefore, the inhibitory effect of RM@TNT on the glycolytic signaling pathway in lung tissue was investigated. As displayed by RT‐qPCR, mRNA levels of glycolysis‐related genes, including *Pfkfb3*, *Pfkp*, *Pkm*, and *Ldha*, were upregulated in SA‐ARDS mice (G2) (Figure S20D). Following the application of Nob, TNT, M@TNT, or RM@TNT, these genes were dramatically downregulated, with the most significant reduction observed in the RM@TNT group. Such a trend was also corroborated by western blotting analysis (Figure 7Q; Figure S20E–H). Moreover, the level of lactic acid, a product of glycolysis, in the BALF was significantly suppressed following RM@TNT treatment (Figure 7R), further indicating that RM@TNT robustly inhibited pulmonary glycolytic activity. Collectively, the underlying mechanism of RM@TNT in treating SA‐ARDS lies in suppressing glycolysis, a process that inhibits M1 macrophage polarization, thereby alleviating excessive inflammation and oxidative stress in the lungs.

To confirm that the therapeutic efficacy of RM@TNT is dependent on the BMAL1 pathway and the active targeting mechanism, we introduced two additional experimental controls. First, when RM@TNT was administered to BMAL1‐knockdown (BMAL1‐KD) SA‐ARDS mice, its ability to suppress pulmonary inflammation, oxidative stress, and lactic acid accumulation was substantially neutralized (Figure S22). Second, competitive blockade of targeted receptors on alveolar macrophages by pre‐treatment with an excess of free Tuftsin abolished the therapeutic efficacy of RM@TNT, resulting in exacerbated lung injury compared to the non‐blocked group (Figure S23). These findings explicitly demonstrate that the superior efficacy of our formulation originates from its precise AM‐targeting capability and the subsequent activation of the BMAL1‐mediated metabolic regulatory axis.

Finally, the overall therapeutic efficiency of RM@TNT was detected. Notably, while the CLP model was used for mechanistic studies, a lethal‐dose LPS‐induced model (15 mg·kg^−^^1^, i.p.) was employed for survival and thermoregulation assays to ensure temporal consistency in systemic inflammation. As shown in Figure 7S, the body temperature of SA‐ARDS mice (G2) decreased dramatically over time (from 33.3°C ± 0.6°C to 28.1°C ± 1.6°C at 24 h), as compared to the sham group (G1). In contrast, the application of Nob, TNT, or M@TNT led to partial alleviation of the reduction in body temperature (to 29.2°C ± 1.8°C, 30.6°C ± 2.0°C, and 30.6°C ± 2.0°C, respectively, after 24 h of treatment), while the administration of RM@TNT restored body temperature to near‐normal levels (33.0°C ± 2.6°C). Of particular note, administration of RM@TNT pronouncedly improved survival rates of SA‐ARDS mice (48‐h survival increased from 0% to 75%), far exceeding those observed in the Nob (25%), TNT (38%), or M@TNT (44%) treatment groups (Figure 7T).

To further enhance the translational relevance of this study, we performed in vivo survival analyses to preliminarily evaluate the therapeutic time window and dose–response relationship (n = 6 per group). Delayed administration of RM@TNT at 2 h post‐challenge showed a survival trend comparable to treatment at 0.5 h, suggesting a potential early therapeutic window (Figure S24). Although the survival improvement attenuated when administration was delayed to 6 and 12 h—attributed to the rapid and aggressive progression of severe sepsis—RM@TNT still provided a certain degree of survival benefit compared to the untreated group. This sustained efficacy underscores its therapeutic advantage across multiple disease stages. We next assessed the dose–response relationship using 5, 10, and 20 mg·kg^−^^1^ (based on Nob). As shown in Figure S25, 5 mg·kg^−^^1^ provided limited protection, whereas 10 mg·kg^−^^1^ and 20 mg·kg^−^^1^ produced comparable survival benefits. As no additional improvement was observed at 20 mg·kg^−^^1^, possibly due to the limited sample size, 10 mg·kg^−^^1^ was selected as the standard effective dose for subsequent experiments. Collectively, these results define preliminary therapeutic parameters and support the translational potential of this strategy.

### 2.14 Biosafety Evaluation of RM@TNT

To assess the clinical translation potential of RM@TNT, we evaluated its biotoxicity both in vitro and in vivo. We initially co‐incubated RM@TNT with normal cell lines (MH‐S and HUVEC) for 24 h. As demonstrated in Figure S26A, even at a concentration of 160 µm (based on Nob concentration), no significant reduction in cell viability was observed. In contrast, free Nob significantly reduced cell viability at a concentration of 80 µm. To further assess safety, RM@TNT was incubated with freshly collected red blood cells from healthy mice. The results showed that RM@TNT at all tested concentrations, as well as the PBS control group, did not induce hemolysis (Figure S26B,C). No significant swelling or rupture of erythrocytes was observed microscopically, with cells maintaining healthy morphology characterized by the typical “biconcave disc” shape (Figure S26D).

In vivo, healthy mice received intranasally administeration of RM@TNT (equivalent to 10 mg·kg^−^^1^ Nob). Analysis at 48 h post‐treatment revealed no significant differences in red blood cell (RBC), white blood cell (WBC), platelet (PLT) counts, or haemoglobin (HBG) levels, between the RM@TNT‐treated group and the saline control group, indicating no adverse effects on the hematopoietic system (Figure S26E). Plasma biochemical markers—including alanine transaminase (ALT), aspartate transaminase (AST), blood urea nitrogen (BUN), and creatinine (CRE)—showed no significant abnormalities (Figure S26F). Moreover, no pronounced changes in body weight were detected between groups. Histopathological examination of major organs (heart, liver, spleen, lung, and kidney) 48 h post‐administration revealed no evidence of tissue damage (Figure S26G).

## 3 Discussion

Our findings reveal that BMAL1 is significantly downregulated in both the sepsis ARDS mouse model and LPS‐induced macrophages. BMAL1 has been shown in previous studies to play a pivotal role in immune regulation by controlling macrophage polarization, cellular metabolism, and cytokine secretion, thereby maintaining immune homeostasis [50]. Our experimental data further demonstrate that, in SA‐ARDS, BMAL1 downregulation is closely associated with M1 macrophage polarization, excessive release of inflammatory cytokines, and heightened oxidative stress. These observations suggest that BMAL1 may serve as a pivotal regulatory factor in immune dysregulation induced by sepsis.

Through comprehensive gain‐ and loss‐of‐function studies, we demonstrated the indispensable role of BMAL1 in restraining macrophage inflammation. In sepsis, glycolytic hyperactivation directly drives M1 polarization and exacerbates inflammatory damage [51]. Mechanistically, BMAL1 actively suppresses this metabolic reprogramming by negatively regulating PFKFB3. To investigate whether this regulatory mechanism extends directly to other key glycolytic nodes, we evaluated the effects of overexpressing PFKFB3, PFKP, PKM2, and LDHA. The functional restoration of PFKP exacerbated inflammatory damage to a degree similar to PFKFB3, reflecting their closely related upstream positions in the glycolytic pathway. Conversely, overexpressing the downstream enzymes PKM2 and LDHA induced comparatively milder phenotypic reversals. Furthermore, these specific genes were absent from the top predicted direct transcriptional targets of BMAL1 (Figure 2K). Combined with their differential rescue efficacies, our results indicate that PFKP, PKM2, and LDHA are not direct primary targets of BMAL1. Instead, their systemic downregulation is presumably an indirect consequence driven by the overall dampening of the glycolytic flux.

In terms of therapeutic strategy, TNT nanoparticles were constructed by loading the BMAL1 agonist Nob onto Tuftsin‐functionalized TDNs. In vitro experiments confirmed that TNT effectively restored BMAL1 expression in macrophages, thereby curtailing glycolytic hyperactivation and attenuating inflammatory responses. To maximize its in vivo bioavailability and therapeutic efficacy in SA‐ARDS models, TNT was further cloaked in ROS‐responsive, lipid‐hybridized macrophage membranes to formulate RM@TNT. This biomimetic coating acts as a decoy to neutralize LPS and inflammatory cytokines while enabling rapid drug release triggered by the high‐ROS microenvironment. Regarding this ROS‐responsive behavior, the 100–200 µm H_2_O_2_ utilized in vitro serves as a controlled oxidative challenge to simulate moderate inflammatory stress, rather than an exact representation of highly heterogeneous in situ peroxide concentrations [52, 53, 54]. Crucially, the indispensable role of the Tuftsin targeting moiety was explicitly validated in vivo. Competitively blocking alveolar macrophage receptors with free Tuftsin significantly abrogated the therapeutic efficacy of RM@TNT, confirming that our robust outcomes are driven by precise peptide‐mediated active targeting rather than mere passive pulmonary accumulation.

These results suggest that the BMAL1 agonist‐based nanoplatform not only improves macrophage function and alleviates inflammation but also protects lung tissue from injury by regulating glycolysis and oxidative stress. More importantly, we definitively proved that the core therapeutic efficacy of our nanoplatform is intrinsically BMAL1‐dependent. Macrophage‐specific knockdown of BMAL1 substantially compromised the anti‐glycolytic and anti‐inflammatory effects of TNT in vitro and largely neutralized the protective outcomes of RM@TNT in SA‐ARDS mice. These mechanistic rescue experiments confirm that our nanodrug exerts its potent targeted effects precisely through the activation of the BMAL1 pathway. Notably, in vivo drug distribution and biosafety assessments showed that RM@TNT possesses excellent targeting and safety profiles, providing strong support for its potential in clinical applications.

However, despite the promising advancements in BMAL1 agonist treatment for sepsis‐associated acute lung injury, some challenges remain. First, the long‐term biosafety and immune response of the nanoplatform require further evaluation. Second, certain key mechanistic experiments were conducted with relatively small sample sizes (e.g., *n* = 3). We explicitly acknowledge the inherent statistical limitations of such sample sizes, which may constrain the statistical power and broad generalizability of these specific findings. Additionally, regarding the optimization of clinical parameters, it is important to note that our current survival analyses mapping the therapeutic window and dosage remain preliminary. Although initial evaluations suggested a potential early intervention window at 2 h and supported 10 mg·kg^−^^1^ as a balanced standard dose for our current models, the therapeutic efficacy notably declined at delayed time points (6 and 12 h). Therefore, the optimal dosage regimen, administration frequency, and exact therapeutic time window of the BMAL1 agonist necessitate further comprehensive exploration in future studies to ensure maximum clinical efficacy.

Overall, this study highlights the potential role of BMAL1 in SA‐ARDS and, through the design of a nanoplatform for targeted delivery of BMAL1 agonists, introduces a novel therapeutic strategy for sepsis‐induced acute respiratory distress syndrome. This approach not only offers a new way to utilize BMAL1 as a therapeutic target but also lays an important theoretical foundation for future research on the regulatory role of immune metabolism in inflammatory diseases.

## 4 Conclusion

In summary, downregulation of BMAL1 represents a key pathophysiological feature in SA‐ARDS. Artificially and precisely increasing BMAL1 expression in AMs can effectively treat SA‐ARDS, which primarily involves inhibiting cellular glycolysis through the BMAL1/PFKFB3 pathway (a metabolic reprogramming therapeutic strategy). Specifically, BMAL1 directly binds to a specific region on the promoter of *Pfkfb3* and transcriptionally suppresses the expression of this gene, thus downregulating the translational level of PFKFB3 (a key enzyme in the glycolysis process). The nanoplatform (RM@TNT) developed in this study demonstrates long‐term retention in diseased lungs after inhalation, ROS‐responsive TNT release, and precision targeting of AMs, which achieves targeted delivery of Nob (a BMAL1 agonist) to AMs, thereby inhibiting AM glycolysis, M1 polarization, excessive inflammatory responses, and oxidative stress. Importantly, treatment with RM@TNT significantly attenuated pulmonary inflammation, tissue damage, and edema in SA‐ARDS mice, leading to a marked improvement in survival rate. This targeted metabolic reprogramming strategy positions RM@TNT as a highly promising precision therapeutic option for SA‐ARDS and sepsis in clinical practice.

## 5 Statistical Analysis

All quantitative data are presented as mean ± standard deviation (SD). Statistical analyses were performed using GraphPad Prism 8.0 software. The normality of data distributions was assessed using the Shapiro‐Wilk test, and the homogeneity of variances was confirmed by Brown‐Forsythe tests. Comparisons between two groups were analyzed using an unpaired Student's t‐test. Multiple group comparisons were evaluated using one‐way analysis of variance (ANOVA) followed by Tukey's post hoc test. A P value of < 0.05 was considered statistically significant. Significance levels are indicated as follows: ^*^ *p* <0.05, ^**^ *p* <0.01, ^***^ *p* <0.001; *ns*: no significant difference.

## Conflicts of Interest

The authors declare no conflicts of interest.

## Supporting information




**Supporting File 1**: advs75093‐sup‐0001‐SuppMat.docx.

**Supporting File 2**: advs75093‐sup‐0002‐DataFile.xlsx.

## Data Availability

The data that support the findings of this study are available from the corresponding author upon reasonable request.
