# Supplementary material for: Tetrahedral DNA Nanostructure‐Based Biomimetic Nanovesicles Attenuate Sepsis‐Associated ARDS by Suppressing Glycolysis via the BMAL1/PFKFB3 Axis
Source: Adv Sci (Weinh). 2026 Apr 20;13(34):e23782. doi: 10.1002/advs.202523782 (PMC13285138; doi:10.1002/advs.202523782)
Supplement: Supplementary file 1 — Supporting File 1: advs75093‐sup‐0001‐SuppMat.docx. [file ADVS-13-e23782-s001.docx]

Supporting Information

**Tetrahedral DNA Nanostructure-Based Biomimetic Nanovesicles Attenuate Sepsis-Associated ARDS by Suppressing Glycolysis via the BMAL1/PFKFB3 Axis**

Yunlong Zhang,^‡^ Bin Li,^‡^* Zhijin Fan,^‡^ Yan Yan, Fei Ma, Changting He, Shiping Liu, Mingliang Pan, Zhou Pan, Huijuan Wang, Xinting Fu, Jiamei Wang, Yue Jia, Qin Gu, Duo Jiang, Xueting Liu, Bohua Ren, Qinqin Wang, Yuehua Hei, Han Duan, Yanqiu Wu, Zihui Wei, Liying Zhan,* and Yuhui Liao*

Y. Zhang, Y. Yan, S. Liu, M. Pan, Z. Pan, H. Wang, L. Zhan

Department of Critical Care Medicine & Department of Emergency, Renmin Hospital of Wuhan University, Wuhan, Hubei 430060, China

E-mail: zhanliying@whu.edu.cn

B. Li, C. He, Q. Gu, D. Jiang, X. Liu, B. Ren, Q. Wang, Y. Hei, Y. Liao

School of Inspection, Ningxia Medical University, Yinchuan, Ningxia 750004, China

E-mail: libin63@alumni.sysu.edu.cn; liaoyh8@mail.sysu.edu.cn

Z. Fan, J. Wang, Y. Liao

Institute for Engineering Medicine, Kunming Medical University, Kunming, Yunnan 650500, China

B. Li, F. Ma, Y. Jia

School of Basic Medical Sciences, Ningxia Medical University, Yinchuan, Ningxia 750004, China

X. Fu, H. Duan, Y. Wu, Z. Wei

Department of Microbiology, School of Public Health, Southern Medical University, Guangzhou, Guangdong 510515, China

‡These authors contributed equally to this work.

**Material and Methods**

*Reagents*

Dulbecco’s modified eagle medium (DMEM), Opti-minimal essential medium (MEM), RPMI 1640 medium, fetal bovine serum (FBS), penicillin/streptomycin (P/S), 2-mercaptoethanol, and phosphate-buffered saline (PBS) were purchased from Gibco Life Technology. Ethanol absolute and xylene were acquired from Sinopharm Chemical Reagent Co. Ltd. Dichloromethane (DCM) was purchased from Energy Chemical. DNA size marker (Cat. No. C109100) was acquired from Bioptic. GelRed nucleic acid stain, Cyanine5 (Cy5), DNA ladder (Cat. No. B600303-0100) and Tuftsin (TKPR) peptide were sourced from Sangon Biotech. Formaldehyde, Coumarin 6 (C6) and sodium bicarbonate (NaHCO_3_) were purchased from Aladdin. Nobiletin (Nob) was acquired from Macklin. DSPE-TK-PEG_2000_ was provided by Xi’an Ruixi Biological Technology. Cholesterol (Cat. No. HY-N0322) and soybean phosphatidylcholine (SPC, Cat. No. HY-125853) were obtained from MedChemExpress. TNF-α and IL-6 mouse enzyme-linked immunosorbent assay (ELISA) kits were purchased from Dakewe Biotech Co. Ltd. Polybrene plus was supplied by Obio Technology Co. Ltd. The mitochondrial stress test kit (Cat. No. 103015-100) and glycolysis stress test kit (Cat. No. 103020-100) were purchased from Agilent Technology. Rabbit anti-mouse β-actin antibody (RRID: AB_2305186) was purchased from Abcam. Rabbit anti-mouse CD86 (RRID: AB_2892094), mouse EGF-like module-containing mucin-like hormone receptor-like 1 (F4/80, RRID: AB_2799771), BMAL1 (RRID: AB_2728705) and horseradish peroxidase (HRP)-conjugated goat anti-rabbit IgG (RRID: AB_2099233) antibodies were from Cell Signaling Technology. Rabbit anti-mouse 6-phosphofructo-2-kinase/fructose-2,6-biphosphatase 3 (PFKFB3, RRID: AB_2162854), pyruvate kinase M2 (PKM2, RRID: AB_1851537), phosphofructokinase, platelet (PFKP, RRID: AB_2252278), lactic acid dehydrogenase A (LDHA, RRID: AB_10646429), and tetramethylrhodamine isothiocyanate (TRITC)-conjugated goat anti-rabbit IgG (RRID: AB_2889939) antibodies were obtained from Proteintech. Protein dual-color markers (Cat. No. 26619), lipofectamine 3000 transfection kit, LIVE/DEAD™ Fixable Aqua Viability Kit (Cat. No. L34991)**,** phycoerythrin (PE)-conjugated CD86 Monoclonal Antibody (RRID:AB_465770), and fluorescein 5-isothiocyanate (FITC)-conjugated F4/80 Monoclonal Antibody (RRID:AB_2637191) were sourced from Thermo Fisher Scientific.

Reactive oxygen species (ROS) detection kit (Dihydroethidium, DHE, Cat. No. S0064S) , ROS detection kit (2',7'-Dichlorodihydrofluorescein Diacetate, DCFH-DA, Cat. No. S0033S), BCA protein assay kit (Cat. No. P0012), RNA Isolation Kit (Cat. No. R0027), non-fat dry milk, RIPA lysis buffer, 4',6-diamidino-2-phenylindole (DAPI), Coomassie Blue Staining Solution, Tris Acetate-EDTA buffer (TAE), Agarose, cell counting kit-8(CCK-8, C0037), 1,1'-dioctadecyl-3,3'-tetramethylindocarbocyanine perchlorate (DiO), 1,1'-dioctadecyl-3,3,3',3'-tetramethylindocarbocyanine perchlorate (DiI), 1,1'-dioctadecyl-3,3',3'-tetramethylindodicarboximide perchlorate (DiD), and chromogenic limulus amebocyte lysate (LAL) endotoxin detection kit (Cat. No. C0276S) were purchased from Beyotime Biotechnology. TM buffer (pH 8.0) was acquired from Mreda Technology. Puromycin was provided by Biosharp. Phenylmethylsulfonyl fluoride (PMSF), trypsin-ethylene diamine tetraacetic acid (EDTA) solution, 4% paraformaldehyde (PFA), and L-lactic acid assay kit (Cat. No. BC2235) were obtained from Solarbio. Running buffer powder, and transfer buffer powder were purchased from GenScript. Goat Serum was obtained from Boster. Protein loading buffer was sourced from Coolaber. Lipopolysaccharide (LPS, Cat. No. L2880) and type V collagenase (Cat. No. C9263) were obtained from Sigma-Aldrich. Hematoxylin-eosin (H&E) staining solution, Cyanine3(Cy3)-tyramide, 488-tyramide, anti-fluorescence quenching sealing agent, hydrogen peroxide (H₂O₂), bovine serum albumin (BSA), Diaminobenzidine (DAB) chromogenic kit, neutral balsam, EDTA antigen retrieval solution (pH 9.0), citrate antigen retrieval solution (pH 6.0) were obtained from Bioqiandu Technology Co. Ltd. Evo M-MLV RT Reaction Premix (Cat. No. AG11728) and SYBR Green Premix Pro Taq HS qPCR Kit (Cat. No. AG11701) were obtained from Accurate Biology.

*Cell lines and culture*

The mouse alveolar macrophage cell line (MH-S, CL-0597, RRID: CVCL_3855), human umbilical vein endothelial cell line (HUVEC, CL-0675), and human embryonic kidney cell line (293T, CL-0005, RRID: CVCL_0063) were procured from Wuhan Pricella Biotechnology Co., the mouse lung epithelial cell line (MLE-12) was purchased from Jinyuan Biotechnology Co., Ltd. MH-S cells were cultured in RPMI 1640 medium supplemented with 10% heat-inactivated FBS, 1% penicillin/streptomycin (10,000 U·mL^-1^ penicillin and 10 mg·mL^-1^ streptomycin), and 0.05 mM 2-mercaptoethanol. HUVEC cells and 293T cells were maintained in DMEM containing 10% FBS and 1% P/S. MLE-12 cells were cultured in DMEM/F-12 medium supplemented with 10% FBS and 1% P/S. All cell lines were incubated in a sterile humidified atmosphere with 5% CO₂ at 37°C and passaged at 70-80% confluency using 0.25% trypsin-EDTA.

*In vitro sepsis model construction*

To investigate the molecular mechanisms of BMAL1 and evaluate the therapeutic efficacy of the nanoplatforms, an *in vitro* sepsis model was established using MH-S alveolar macrophages. To induce inflammatory injury, cells were challenged with 1 μg·mL⁻¹ lipopolysaccharide (LPS) for 24 h. The subsequent mechanistic and therapeutic evaluations were systematically categorized into genetic manipulation and pharmacological intervention modules.

For comprehensive gain- and loss-of-function studies, specific genetic modifications were performed prior to LPS stimulation. Notably, to ensure consistent and robust gene expression, BMAL1 overexpression was achieved by transducing MH-S cells with recombinant lentiviral vectors, followed by appropriate antibiotic selection to establish a stable BMAL1-overexpressing cell line (designated as OE). Conversely, BMAL1 knockdown was performed using adeno-associated virus (AAV) vectors, which were transduced into the wild-type cells 48 h prior to the LPS challenge

To further dissect the downstream metabolic networks, a series of rescue experiments were conducted. BMAL1-overexpressing cells were transfected with plasmids encoding specific glycolytic enzymes, including PFKFB3, PFKP, PKM2, or LDHA, at 24 h prior to LPS exposure. Corresponding empty vectors were utilized as negative controls (NC).

For the assessment of therapeutic formulations, cells were first exposed to 1 μg·mL⁻¹ LPS to initiate the sepsis model. One hour post-exposure, the cells were treated with either PBS (vehicle control), free Nob (16 μM), TN (16 μM, calculated as Nob equivalent), or the targeted nanoplatform TNT (16 μM, calculated as Nob equivalent).

Based on these standardized protocols, cells were flexibly allocated into specific control, model, genetic intervention, or drug treatment groups according to the analytical requirements of each distinct assay.

*Establishment of a sepsis-associated ARDS (SA-ARDS) mouse model*

Animal experiments were approved by the Experimental Animal Ethics Committee of Renmin Hospital of Wuhan University (Approval No. 20230406A) and conducted in compliance with “Animal Research: Reporting of *In Vivo* Experiments” (ARRIVE) guidelines. Male C57BL/6 mice (6–8 weeks old, RRID:MGI:2159769) were obtained from the Guangdong Medical Laboratory Animal Center (Guangdong, China) and maintained under specific pathogen-free (SPF) conditions with a 12 h light/dark cycle, ad libitum access to food and water. The SA-ARDS mouse model was established through standard cecal ligation and puncture (CLP),^[1]^ which was used to build sepsis mouse model. In detail, mice were first fasted for 6 h and anesthetized *via* isoflurane inhalation. Following abdominal hair removal and disinfection, a 1-1.5 cm midline laparotomy was performed to expose the cecum. The distal half of the cecum was ligated with silk suture, punctured twice with a 21-gauge needle, and gently compressed to extrude fecal content. The cecum was returned to the abdominal cavity, and the incision was sutured in layers. Immediately following surgical procedures, fluid resuscitation was initiated *via* subcutaneous administration of 1 mL pre-warmed (37°C) sterile saline solution. To confirm successful establishment of SA-ARDS model, lung tissue samples were collected at 3, 6, and 12 hours post-CLP for histopathological analysis; consistent pathological features of ARDS confirmed model validity.

*Lentiviral transduction and plasmid transfection*

The BMAL1-overexpressing lentivirus was constructed by Obio Technology using the GV341 vector. For transduction, MH-S cells at 30–40% confluency were incubated with serum-free RPMI 1640 medium containing lentiviral particles at a titer of 1 × 10⁸ TU·mL⁻¹ and polybrene (8 μg·mL⁻¹) for 8 h. The medium was replaced with complete RPMI 1640, and cells were cultured for 72 h. Transfection efficiency (>70%) was confirmed by fluorescence microscopy (GFP signal), real-time quantitative PCR (qPCR), and Western blotting (WB). Experimental groups included untreated MH-S cells (Normal), cells transduced with empty GV341 vector (Negative control, NC), and cells transduced with BMAL1-overexpressing lentivirus (BMAL1-OE).

For the targeted BMAL1 knockdown (KD) experiments, an adeno-associated virus (AAV) vector expressing a short hairpin RNA against BMAL1 (shBMAL1) driven by the macrophage-specific F4/80 promoter was utilized. The specific shRNA sequence targeting BMAL1 was forword: 5'-CCGAAUGCUGAGGAATT-3′ and reverse: 5'-UUCCUCAGCAAUCAUUCGGTT-3′ and reverse: 5'-ACGUGACACGUUCGGAGAATT-3′.. The sequence of the si-NC was forward: 5'-UUCUCCGAACGUGUCACGUTT-3′ MH-S cells were transduced with the AAV particles according to the manufacturer’s optimized multiplicity of infection. The specific knockdown efficiency was subsequently validated via qPCR and Western blotting prior to further treatments.

To dissect the downstream metabolic targets, rescue experiments were conducted by transiently transfecting the stable BMAL1-OE cells with plasmids overexpressing specific glycolytic enzymes, including PFKFB3, PFKP, PKM2, and LDHA (synthesized by Obio Technology). Transfections were performed using the Lipofectamine 3000 reagent according to the manufacturer’s protocol. The designated experimental groups comprised BMAL1-OE cells transfected with empty control plasmids (OE + Vector) and BMAL1-OE cells transfected with the respective target plasmids (designated as OE + PFKFB3, OE + PFKP, OE + PKM2, and OE + LDHA, respectively).

*Western blotting analysis*

The protein levels of BMAL1, PFKFB3, PFKP, PKM2, and LDHA were analyzed by WB. Cells were lysed in RIPA buffer supplemented with 1% PMSF on ice for 10 minutes, followed by centrifugation at 14,000 ×g for 10 minutes at 4°C to collect the supernatant. For lung tissues, approximately 20 mg samples were homogenized in RIPA buffer supplemented with 1% PMSF using a cryogenic grinder with liquid nitrogen cooling. The homogenates were then centrifuged under identical conditions (14,000 × *g*, 10 min, 4°C) to obtain protein supernatants. Protein concentrations were determined using a BCA Protein Assay Kit, and samples were mixed with protein loading buffer and heated at 95°C for 10 minutes. Equal amounts of protein (20 μg per lane) were separated by SDS-PAGE and transferred onto PVDF membranes. The membranes were blocked with 5% non-fat milk for 1 hour at room temperature and then incubated overnight at 4°C with primary antibodies against BMAL1, PFKFB3, PFKP, PKM2, LDHA, and β-actin (rabbit anti-mouse IgG). After washing, the membranes were incubated with HRP-conjugated goat anti-rabbit secondary antibody for 1 hour at room temperature. Protein bands were visualized using a chemiluminescence imaging system (Amersham, ImageQuant 800) and quantified *via* grayscale analysis with Chemiluminescence Imaging software. Quantitative data were normalized to β-actin expression levels.

*Real-time quantitative PCR*

Total RNA was isolated using the RNA Isolation Kit for gene expression analysis. Complementary DNA (cDNA) was synthesized from RNA using the Evo M-MLV RT Reaction Premix following the manufacturer’s protocol. Quantitative PCR was performed with the SYBR Green Premix Pro Taq HS qPCR Kit on a Real-Time PCR System (DLAB Scientific, Accurate 96). The relative expression levels of target genes were calculated using the 2−ΔΔCt method and normalized to the housekeeping gene *Actb*. Primer sequences are listed in Table S2.

*RNA sequencing (RNA-Seq) and bioinformatics analysis*

Total RNA was isolated from NC and BMAL1-OE MH-S cells, with RNA integrity verified using an Agilent 2100 Bioanalyzer (Agilent Technologies) and concentration quantified *via* NanoDrop spectrophotometry. mRNA was enriched using oligo-dT magnetic beads and fragmented. First- and second-strand cDNA synthesis was performed using reverse transcriptase, followed by end repair, adenylation, and Illumina adapter ligation. PCR amplification with adapter-specific primers generated sequencing libraries, which were purified using magnetic beads. Library quality was assessed *via* Qubit fluorometric quantification and Agilent Fragment Analyzer.

Sequencing was conducted on the Illumina NovaSeq 6000 platform with PE150 configuration (paired-end 150 bp reads). Raw sequencing data underwent quality control using FastQC, followed by alignment to the GRCm38.102 reference genome (GENCODE M31 annotation) using HISAT2. Differential gene expression patterns (DEGs) were visualized using ggplot2 (v3.5.2) in R software (v4.5.0). Volcano plots visualized differential gene expression with thresholds of |log_2_FC| > 4 and adjusted p-value < 0.01, employing enhanced aesthetics through the ggrepel package for label positioning. Heatmaps displayed Z-score normalized expression values of significant genes using the pheatmap package with hierarchical clustering. Functional enrichment analysis of DEGs was performed through Gene Ontology (GO) and Kyoto Encyclopedia of Genes and Genomes (KEGG) pathway analyses. The results were visualized using bubble plots generated *via* the online platform Bioinformatics.com.cn (https://www.bioinformatics.com.cn/plot_basic_gopathway_enrichment_bubbleplot_081). ^[2]^

*Public database analyses*

BMAL1 Chromatin Immunoprecipitation-Sequencing (ChIP-seq) data in murine macrophages^[3]^(accession GSM2522477) were retrieved from the Cistrome DATA Browser^[4]^ (http://cistrome.org/db/#/) and visualized using the UCSC Genome Browser (GRCm38/mm10 assembly). Glycolysis pathway gene sets were curated through KEGG (https://www.kegg.jp) and Gene Ontology (http://geneontology.org) databases. Putative BMAL1 binding motifs were predicted using JASPAR 2024 (https://jaspar.elixir.no), ^[5]^ with subsequent analysis of potential BMAL1 binding sites within the *Pfkfb3* promoter region (-2000 to +200 bp relative to TSS) performed with a relative profile score threshold >80%.

*ChIP-qPCR assay*

BMAL1-overexpressing MH-S cells (8 × 10⁶) were cross-linked with 1% formaldehyde in PBS for 10 min at room temperature. The reaction was quenched with 125 mM glycine for 5 min. After two washes with ice-cold PBS, cells were lysed in ChIP lysis buffer (1% SDS, 10 mM EDTA, 50 mM Tris-HCl, pH 8.0) containing protease inhibitors. Chromatin was fragmented using a Scientz-IID Ultrasonic Cell Disruptor (Ningbo Scientz) at 30% amplitude (15 sec ON/3 min OFF, 4 cycles) to generate 200-1000 bp fragments.

5% of the lysate was reserved as input control. The remaining lysate was incubated with 0.8 μg anti-BMAL1 antibody or IgG control overnight at 4°C, followed by 2 hr incubation with Protein A/G magnetic beads. After reversing cross-links at 65°C for 6 hr, DNA was purified using a DNA purification kit. Enrichment of BMAL1 binding at the PFKFB3 promoter was quantified by qPCR with primers: *Pfkfb3*-forward (5′-AGCAGGCTGTCTCTTG-3′) and *Pfkfb3*-reverse (5′-TCTCCAGTTCTGTCGG-3′).Data were normalized to input DNA using the ΔΔCt method.

*Dual-luciferase reporter assay*

Wild-type (WT) and mutant (mut) *Pfkfb3* promoter sequences (synthesized by Obio Technology) were cloned into the pGL4.1-Basic vector. All constructs were verified by Sanger sequencing. 293T cells seeded in 96-well plates were transfected at 70% confluency with 0.1 μg firefly luciferase reporter, 0.005 μg Renilla luciferase vector, and 0.1 μg expression plasmids using lipofectamine 3000 transfection reagent. After 24 h, luciferase activity was measured using the Dual-Luciferase® Reporter Assay System (Promega E1960). Firefly/Renilla luciferase ratios were calculated to normalize transfection efficiency.

*Macrophage targeting evaluation*

To assess the macrophage-targeting capability of TNT, C6 was employed as a fluorescent analog of Nob for enhanced visualization. C6-loaded TDN (TC) and Tuftsin-conjugated TC (TCT) were prepared using the identical protocol established for TN, TNT synthesis, with C6 substituted for Nob at the same mass ratio.

Free C6, TC, or TCT were incubated with MH-S or HUVEC cells at 37°C for varying durations (0.5, 1, 2, 4, and 12 h). Following incubation, cells were washed three times with PBS to remove uninternalized nanoparticles. Additionally, to assess the macrophage-targeting efficacy of Tuftsin, cells were pretreated with 100 μg·mL⁻¹ Tuftsin for 1 h prior to administration of free C6, TC, or TCT nanocomplexes. Control groups received equivalent volumes of PBS pretreatment.

Fluorescence microscopy was performed to evaluate cellular internalization of C6, TC, or TCT. Imaging was conducted using an inverted fluorescence microscope (Olympus IX73) equipped with GFP filter sets (excitation 470-490 nm, emission 510-550 nm). Three random fields per sample were captured at 20× magnification. Mean fluorescence intensity (MFI) was quantified using ImageJ software with consistent thresholding and background subtraction applied across all images.

Cellular uptake efficiency was further confirmed by flow cytometry (excitation/emission: 488/504 nm) using an Attune™ NxT AFC2 flow cytometer (Thermo Fisher Scientific). Cells were analyzed at a flow rate of 300-500 events/sec, with fluorescence intensity quantified in the FITC channel. Data analysis was performed using FlowJo software.

*Cytokine and LPS binding assay*

The expression levels of receptors for TNF-α (TNFR), IL-6 (IL-6R), and LPS (TLR4) in MM, M@TNT, and RM@TNT were analyzed by WB. Samples were evaluated at the same protein concentrations (1 mg·mL⁻¹).

To evaluate the binding capacity of TNT, M@TNT, and RM@TNT toward cytokines and LPS, IL-6 (500 pg), TNF-α (500 pg), or LPS (300 ng) were incubated with PBS (control), TNT, M@TNT, or RM@TNT at 37°C for 2 h. The concentration of all nanoparticles was maintained at 16 μM (calculated as Nob equivalent). After incubation, the mixtures were centrifuged at 16,000 × *g* for 10 min to collect supernatants. Unbound IL-6 and TNF-α in supernatants were quantified using ELISA kits, while unbound LPS was measured with a chromogenic LAL endotoxin assay kit according to the manufacturer's instructions. Bound cytokines/LPS were calculated by subtracting the unbound concentrations from the initial concentrations.

*Mitochondrial respiration and glycolytic fux analysis*

To assess mitochondrial respiration (OCR) and glycolytic flux (ECAR) in LPS-treated MH-S macrophages, cellular metabolism was profiled using the Seahorse XFe96 Analyzer (Agilent Technologies, USA). Cells were seeded at 10,000 cells/well in XF96 plates and cultured overnight. Experimental groups included plasmid transfection, LPS stimulation, and NOB/TN/TNT treatments as described. Prior to analysis, cells were washed and incubated in Seahorse XF base medium (pH 7.4) supplemented with 1 mM pyruvate, 2 mM glutamine, and 10 mM glucose (for ECAR) or 1 mM pyruvate and 2 mM glutamine (for OCR).

Mitochondrial Stress Test:

Baseline OCR was recorded. Sequential injections of 1 μM oligomycin (ATP synthase inhibitor), 1 μM carbonyl cyanide 4-(trifluoromethoxy) phenylhydrazone (FCCP, mitochondrial uncoupler), and 0.5 μM rotenone/antimycin A (Rot&AA, complex I/III inhibitors) were performed to assess basal respiration and maximal respiration.

$$Basal respiration= Baseline OCR-PostRot/AA OCR$$

$$Maximal respiration= PostFCCP OCR-PostRot/AA OCR$$

Glycolytic Stress Test:

Baseline ECAR was measured. Sequential injections of 10 mM glucose, 1 μM oligomycin, and 50 mM 2-DG (glycolysis inhibitor) were used to quantify basal glycolysis and compensatory glycolysis.

$$Basal glycolysis= Postglucose ECAR-Post2DG ECAR$$

$$Compensatory glycolysis= Postoligomycin ECAR-Post2DG ECAR$$

*Membrane fusion analysis*

To evaluate membrane-liposome fusion efficiency, fluorescence resonance energy transfer (FRET) analysis was performed using DiI (λex/em = 549/565 nm) and DiD (λex/em = 644/663 nm). M NVs were labeled by incubating with FRET dyes at 25°C for 30 min, followed by centrifugation (20,000 × *g*, 30 min) to remove unbound dyes. Fluorescently labeled M@TNT and RM@TNT were synthesized sequentially following the aforementioned protocol, with consistent concentrations of fluorescently labeled M NVs maintained throughout the preparation process. Fluorescence spectra (550-750 nm) of pre- and post-fusion complexes were recorded on a Shimadzu RF-6000 spectrofluorometer (λex = 545 nm), with FRET efficiency quantified *via* fluorescence intensity at 565 nm (I565) and 663 nm (I663).

$$FRET efficiency \left( \% \right)= \frac{I_{663}}{I_{663}+I_{565}}\times100$$

For spatial validation, DiO-labeled RL and DiD-labeled M NVs were co-assembled into RM@TNT. Fluorescence colocalization was confirmed by confocal microscopy (100× oil immersion objective,), with sequential imaging of DiO (λex/em = 484/501 nm) and DiD (λex/em = 644/663 nm) channels to minimize crosstalk.

*ROS-responsive drug release assay*

To evaluate the oxidative stress-triggered release of Nob from RM@TNT, a dialysis-based method was performed. Briefly, the RM@TNT suspension (2 mL, 400 μM, calculated as Nob equivalent) was loaded into dialysis bags (MWCO 10 kDa, pre-treated by boiling in 10 mM EDTA for 10 min). The bags were subsequently immersed in 48 mL of respective release media—specifically PBS, simulated lung fluid (SLF), or 10% FBS—maintained at 37°C with gentle agitation (100 rpm). To assess ROS-responsive behavior, the media were supplemented with 0.1 mM or 0.2 mM H₂O₂, with H₂O₂-free media serving as negative controls.

At predetermined intervals (0, 2, 4, 6, 8, 10, 12, 16, 20, and 24 h), 1 mL of the external medium was collected and immediately replaced with an equal volume of fresh, pre-warmed corresponding medium. The collected samples were centrifuged (12,000 rpm, 10 min) to remove residual nanoparticles, and the supernatant Nob concentration was quantified at 334 nm using a UV-Vis spectrophotometer (Shimadzu UV-2600) with a pre-calibrated standard curve.

Cumulative Nob release was calculated as:

$$Cumulative release (\%)=\frac{Total Nob released}{Total Nob loaded in RM@TNT}\times100$$

*Nanoparticle stability assay*

The stability of RM@TNT nanoparticles was comprehensively evaluated under both storage and physiological conditions. For long-term storage stability, RM@TNT was dispersed in PBS (pH 7.4) at a final concentration of 160 μM (calculated as Nob equivalent), aliquoted into sterile microcentrifuge tubes, and stored at 4°C under dark conditions, with samples collected at predetermined time points over a 7-day period. To assess short-term physiological stability, the nanoparticles were incubated in simulated lung fluid (SLF) and 10% fetal bovine serum (FBS) at 37°C, and samples were collected at 0, 6, 12, 18, and 24 h. All collected samples from both stability assays were subsequently analyzed for hydrodynamic diameter and polydispersity index (PDI) using dynamic light scattering (Litesizer 500, Anton Paar).

*Cytokine quantification by ELISA*

IL-6, TNF-α, IL-10 and TGFβ1 concentrations in MH-S cell supernatants and murine BALF were measured using mouse-specific ELISA kits according to the manufacturer's instructions. Briefly, samples were centrifuged at 1,000 × *g* for 10 min at 4°C to remove particulates. Cleared supernatants and standards were added to antibody-precoated wells, followed by incubation with biotinylated detection antibodies at 37°C for 1 hr. After washing with PBS-T buffer, streptavidin-HRP conjugates were added and incubated at 37°C for 30 min. Following additional washes, 3,3',5,5'-tetramethylbenzidine (TMB) substrate was developed for 15 min in darkness. Reactions were terminated with 2M H2SO4 and absorbance measured at 450 nm using an iMark microplate reader (Bio-Rad).

*Intracellular ROS levels detection*

Intracellular ROS levels across various in vitro cellular models and ex vivo pulmonary single-cell suspensions were comprehensively assessed using a panel of specific fluorescent probes, namely DCFH-DA, DHE, and CellROX Orange. The specific probe for each assay was systematically selected based on the fluorescence compatibility requirements of the respective experimental design to avoid spectral overlap (e.g., with GFP-expressing vectors or other fluorophores). Briefly, cells were incubated with the designated ROS probe at 37°C under light-protected conditions following optimized established protocols. Following appropriate washing steps to remove excess dye, the fluorescence signals were acquired using an Attune™ NxT AFC2 flow cytometer (Thermo Fisher Scientific) equipped with the corresponding excitation and emission filter sets for each specific dye. The MFI was subsequently quantified from the flow cytometry data using FlowJo software.

MH-S cells seeded in 6-well plates were stained with either DHE or DCFH-DA probes according to their respective experimental groups, consistent with the protocols described above. Fluorescence imaging was performed using an Olympus IX73 microscope with optimized filter sets: DCFH-DA-stained samples used 488 nm excitation/525 nm emission (GFP channel), while DHE-stained samples employed 535 nm excitation/610 nm emission (TRITC channel). Three random fields per well were captured using a 20× objective, and fluorescence micrographs were quantified for MFI using ImageJ software.

*Flow cytometric analysis of macrophage polarization*

For *in vitro* experiments, MH-S cells were harvested and stained with PE-conjugated anti-mouse CD86 and PerCP/eFluor 710-conjugated anti-mouse CD206 antibodies to assess M1 and M2 polarization, respectively. For *in vivo* studies, single-cell suspensions from lung tissues were prepared via enzymatic digestion and sequentially stained with LIVE/DEAD™ Fixable Aqua viability dye, FITC-conjugated anti-mouse F4/80, PE-conjugated anti-mouse CD86, and PerCP/eFluor 710-conjugated anti-mouse CD206 antibodies. Within the gated live cell population, M1 and M2 macrophages were specifically defined as F4/80⁺CD86⁺ and F4/80⁺CD206⁺ cells, respectively. All samples were acquired using an Attune™ NxT AFC2 flow cytometer (Thermo Fisher Scientific). Flow cytometry data were analyzed using FlowJo software, employing corresponding isotype controls and unstained samples to establish precise gating specificity.

*Immunofluorescence assay*

For *in vitro* experiments, MH-S cells were fixed with 4% PFA, blocked with 5% BSA/PBS, and incubated with rabbit anti-mouse CD86 antibody (1:50) at 4°C overnight. Following primary antibody incubation, samples were treated with TRITC-conjugated goat anti-rabbit IgG secondary antibody (1:100), then counterstained with DAPI for nuclear visualization. Fluorescence imaging was performed using an Andor confocal microscope, with three random fields captured per sample.

Dual-label immunofluorescence was performed on paraffin-embedded mouse lung sections. Following deparaffinization in xylene and rehydration through graded ethanol, antigen retrieval was achieved by microwave heating in EDTA antigen retrieval solution (pH 9.0) for 15 min. Sections were blocked with 10% goat serum for 0.5 h at room temperature, then incubated overnight at 4°C with rabbit anti-mouse F4/80 primary antibody (1:1000). After PBS washes, HRP-conjugated goat anti-rabbit secondary antibody (1:400) was applied for 1 h, followed by Cy3-tyramide signal amplification for 20 min. For sequential co-staining, microwave-mediated antigen retrieval was repeated to denature bound antibodies. After re-blocking with 10% goat serum, sections were incubated with rabbit anti-mouse CD86 (1:400) overnight at 4°C, followed by HRP-conjugated secondary antibody (1:400) and 488-tyramide amplification. Nuclei were counterstained with DAPI for 10 min prior to mounting with anti-fluorescence quenching sealing agent. Imaging was conducted using an Olympus IX73 microscope.

To assess pulmonary ROS levels, rehydrated lung sections were incubated with 10 μM DHE in a light-protected humidified chamber at 37°C for precisely 40 min. The reaction was terminated by three washes with ice-cold PBS, followed by nuclear counterstaining using DAPI for 5 min at room temperature. Oxidized ethidium signals were immediately captured through the TRITC channel (excitation/emission: 518/605 nm) on an Olympus IX73 microscope. Quantitative analysis of MFI was conducted using ImageJ software.

*Lactic acid assay*

The lactic acid concentration in cell supernatants or murine BALF was measured using a L-lactic acid assay kit following the manufacturer’s protocol. Briefly, samples were centrifuged at 1,000 × *g* for 10 min at 4°C to remove particulates. Cleared supernatants and standards were incubated with reagent A (containing lactic acid dehydrogenase and cofactors) at 37°C in the dark for 30 min, during which lactic acid was oxidized to pyruvate with concomitant NADH generation. The reaction was terminated by adding stop solution, and absorbance was measured at 340 nm using a microplate reader. Lactic acid concentrations were calculated based on a standard curve.

*In vitro safety evaluation*

MH-S macrophages and HUVEC cells were seeded in 96-well plates at 5×10³ cells/well and cultured for 24 h at 37°C.Cells were incubated with RM@TNT (0, 5, 10, 20, 40, 80, 160 μM) for 24 h (*n* = 4 per group).10 μL CCK-8 reagent was added to each well, followed by 2 h incubation at 37°C. Absorbance at 450 nm was measured using a microplate reader. Cell viability (%) was calculated as:

$$Viability (\%)=\frac{\mathrm{OD}_{S\mathrm{ample}}-\mathrm{OD}_{\mathrm{Blank}}}{\mathrm{OD}_{Control}-\mathrm{OD}_{\mathrm{Blank}}}\times100$$

Fresh mouse blood was centrifuged (2000 rpm, 10 min), washed 3× with PBS, and resuspended as a 5% RBC suspension. RM@TNT (0, 5, 10, 20, 40, 80, 160 μM) was mixed with RBC suspension (1:1 v/v) and incubated at 37°C for 4 h. Controls included 1% ddH2O (positive) and PBS (negative). After centrifugation (2000 rpm, 10 min), supernatant absorbance at 562 nm was measured. Hemolysis (%) was determined by:

$$Hemolysis (\%)=\frac{\mathrm{OD}_{Sample}-\mathrm{OD}_{\mathrm{Negative}}}{\mathrm{OD}_{Positive}-\mathrm{OD}_{\mathrm{Negative}}}\times100$$

Erythrocyte pellets were resuspended in PBS, smeared on glass slides, and observed for morphological analysis under an optical microscope (Olympus IX7).

*In vivo biosafety evaluation*

Male C57BL/6 mice (8 weeks old) were randomly assigned to two groups and acclimatized for one week under controlled environmental conditions. RM@TNT (10 mg·kg⁻¹ based on the loaded Nob) or saline (control) was administered *via* intranasal instillation. At 24 hours post-administration, blood samples were collected for hematological analysis, including red blood cells (RBC), white blood cells (WBC), platelets (PLT), hemoglobin (HGB), and biochemical markers such as alanine aminotransferase (ALT), aspartate aminotransferase (AST), albumin (ALB), blood urea nitrogen (BUN), and creatinine (CRE). Major organs (heart, liver, spleen, lungs, and kidneys) were harvested, rinsed in PBS, and fixed in 4% PFA for subsequent histopathological examination.

Tissue sections were paraffin-embedded, sliced into thin sections, and stained with H&E. Morphological changes in organs were evaluated under a light microscope to assess potential toxicity.

*Synthesis and characterization of TDN, TN, and TNT*

TDN was synthesized by mixing four single-stranded DNA chains (S1~S4, Table S1, Sangon Biotech) at equimolar ratios in TM buffer (10 mM Tris-HCl, 5 mM MgCl₂, pH 8.0). The mixture was denatured at 95°C for 10 min in a PCR thermal cycler (TC1000-G, DLAB) and cooled to 4°C for 20 min for self-assembly.^[6]^

The stepwise synthesis of TDN was verified through 8% polyacrylamide gel electrophoresis (PAGE, 100 V, 90 min in 1× TAE), 2% agarose gel electrophoresis (AGE, 120 V, 40 min in 1× TAE), and capillary electrophoresis (CE, Qsep100, Bioptic).

TDN and Nob were mixed at 1:20, 1:40, 1:60, 1:80, 1:100, and 1:120 molar ratios in PBS. After 8 h incubation at 4°C with shaking (100 rpm), unbound Nob was removed by ultrafiltration (30 kDa MWCO filter, 10,000 rpm, 4°C, 10 min). The retentate was collected as TN complexes for subsequent characterization.

To determine the optimal ratio for Nob loading, the filtrate was analyzed at 334 nm using an Ultraviolet-visible spectroscopy (UV-Vis) spectrophotometer (Shimadzu).

Encapsulation efficiency (EE) and drug loading capacity (DLC) were calculated to select the optimal ratio:

$$\mathrm{DLC}\left( \% \right)=\frac{Total Nob mass-Free Nob mass}{TN mass}\times100$$

$$\mathrm{EE}\left( \% \right)=\frac{Total Nob mass-Free Nob mass}{Total NOB mass}\times100$$

TNT nanocomplexes were prepared by mixing TN and tuftsin in PBS at 1:200, 1:400, 1:600, 1:800, 1:1000, and 1:1200 molar ratios. The mixtures were incubated at 25°C with orbital shaking (200 rpm) for 30 min. Binding efficiency was analyzed through 2% agarose gel electrophoresis (100 V, 60 min in 1× TAE).

Free Nob, TDN, TN, and TNT were quantified at 334 nm using a UV-Vis spectrophotometer (Shimadzu). Hydrodynamic diameter, polydispersity index (PDI), and zeta potential were analyzed by dynamic light scattering (Litesizer 500, Anton Paar). Morphological evaluation was performed using Transmission Electron Microscopy (TEM, JEOL JEM-F200) and Atomic Force Microscopy (AFM, Bruker Dimension Icon).

*Cell membrane extraction*

MH-S cells cultured in T-175 flasks (80-90% confluence) were harvested using cell scrapers, washed twice with PBS, and pelleted by centrifugation at 500 × *g* for 5 min. The cell pellet was resuspended in cell membrane extraction buffer containing 10 mM NaHCO₃, 0.2 mM EDTA, and 1 mM PMSF (freshly added). After a 15-min incubation on ice, cells were lysed through three freeze-thaw cycles alternating between liquid nitrogen immersion and a 37°C water bath. The lysate was centrifuged at 3000 × *g* (4°C, 15 min) to remove small and large cellular debris, followed by membrane precipitation at 18,000 × *g* (4°C, 60 min). The MH-S cell membranes were collected as pale white pellets and stored at −80°C for subsequent use. Membrane protein concentration was quantified using a bicinchoninic acid (BCA) protein assay kit.

*Synthesis of M@TNT and RM@TNT*

A mixture containing 200 μL of TNT solution (0.8 mM based on), 400 μL of MH-S cell membrane suspension (0.5 mg·mL⁻¹ based on protein concentration), and 400 μL PBS was sonicated in a 37°C water bath using a probe sonicator (52 kHz frequency, 100 W power) for 20 min. The resulting suspension was sequentially extruded through polycarbonate membranes with decreasing pore sizes (800 nm, 400 nm, 200 nm) using a LiposoFast extruder (Avestin) to obtain monodisperse M@TNT nanoparticles.

While maintaining identical preparation parameters, MH-S cell membrane-based nano-vesicles (M NVs) were synthesized without TNT loading.

A lipid film was formed by dissolving 9 mg SPC, 1.5 mg DSPE-TK-PEG2000, and 1.5 mg cholesterol in 12 mL DCM, followed by rotary evaporation (IKA RV10, 40°C, 30 min). The dried film was hydrated with 6 mL PBS at 37°C for 15 min and sonicated (52 kHz, 100 W) for 15 min to generate ROS-responsive liposomes (RL). Subsequently, 200 μL of TNT solution (0.8 mM) was combined with 400 μL alveolar macrophage membrane (MM) suspension (0.5 mg·mL⁻¹) and 400 μL RL suspension (2 mg·mL⁻¹). The mixture was sonicated (52 kHz, 100 W, 37°C, 20 min) and extruded through polycarbonate membranes (800 nm, 400 nm, 200 nm) to yield RM@TNT.

Nanoparticle morphology was analyzed by TEM (JEOL JEM-F200) after staining with 2% uranyl acetate. Hydrodynamic diameter, PDI, and zeta potential were analyzed by dynamic light scattering (Litesizer 500, Anton Paar).

Protein profiles of TNT, RL, MM, M@TNT, RM@TNT, and MH-S cell lysates were analyzed by SDS-PAGE. Samples were separated on 10% polyacrylamide gels at 120 V for 90 min, followed by Coomassie Brilliant Blue staining for 1 h. After destaining, gels were imaged using a Tanon MINI Space 2000 documentation system. Samples were evaluated at the same protein concentrations (1 mg·mL⁻¹), except for the TNT (320 μM based on the loaded Nob) and RL (4mg·mL⁻¹) groups.

*In vivo biodistribution and targeted delivery assessment*

Cy5-labeled nanostructures were prepared through the conjugation of Cy5 with single-stranded DNA S1 within the TDN, generating Cy5-S1 (Table S1) for the construction of Cy5-labeled TNT and Cy5-labeled RM@TNT. Free Cy5 dye was utilized as the non-targeted tracking control. Forty-eight mice were randomly divided into four groups (n = 12 per group): (1) SA-ARDS mice receiving free Cy5, (2) SA-ARDS mice administered Cy5-labeled TNT, (3) SA-ARDS mice treated with Cy5-labeled RM@TNT, and (4) Sham-operated mice treated with Cy5-labeled RM@TNT. All formulations were administered via intranasal instillation at 0.5 h post-modeling or sham operation. To ensure accurate comparative imaging, the administered dosage for the nanoplatform groups was equivalent to 10 mg·kg⁻¹ of Nob, and all formulations were strictly standardized to deliver an equal Cy5 fluorescence intensity across all groups.

At designated time points (0.5, 6, 12, and 24 h post-administration), mice were euthanized via cervical dislocation under 5% isoflurane anesthesia. Major organs (heart, liver, spleen, lungs, kidneys) were immediately excised and subjected to *ex vivo* fluorescence imaging using an *in vivo* imaging system (AniView Phonex600, Biolight Biotechnology). Cy5 signals were captured with excitation and emission wavelengths set at 640 nm and 670 nm, respectively. Lung fluorescence intensity was quantified to assess pulmonary accumulation and retention, with data analyzed as mean fluorescence intensity normalized to the background signal.

*In vivo therapeutic efficiency evaluation of RM@TNT*

Mice were randomly assigned to six groups: Sham (Sham surgery control mice); Model (SA-ARDS mice); Model + Nob (SA-ARDS mice with Nob treatment); Model + TNT (SA-ARDS mice with TNT treatment); Model + M@TNT (SA-ARDS mice with M@TNT treatment); Model + RM@TNT (SA-ARDS mice with RM@TNT treatment).

At 0.5 h post-CLP, mice in the Model + Nob, Model + TNT, Model + M@TNT, and Model + RM@TNT groups received intranasal administration of free Nob (10 mg·kg⁻¹) or equivalent doses of nanoparticle formulations, while the Sham and Model groups received PBS. All mice were euthanized 24 h post-CLP *via* overdose anesthesia and cervical dislocation.

Twelve mice per experimental group were systematically allocated for tissue sampling to maximize data acquisition while minimizing inter-individual variability. For the first six mice, bronchoalveolar lavage fluid (BALF, 0.8 mL) was collected from the left lung for cell counts using a hemocytometer, with total protein (BCA kit), lactic acid (L-lactic acid assay kit), and inflammatory cytokines (TNF-α/IL-6 ELISA) quantified per manufacturer protocols, while the right lung was fixed in 4% PFA for subsequent paraffin embedding, histopathology, and immunofluorescence analysis.

For the remaining six mice, the left lung was divided into superior and inferior regions: the superior lobe was enzymatically digested into single-cell suspensions for intracellular ROS detection *via* DCFH-DA staining and flow cytometry, while the inferior lobe underwent similar processing for M1 macrophage polarization assessment using surface marker staining. The right lung was bisected with the anterior/median lobes immediately weighed for wet mass determination followed by 48 h drying at 60°C to calculate wet/dry (W/D) ratio, while the posterior/caudal lobes were snap-frozen in liquid nitrogen for molecular analyses—protein extracts subjected to WB for BMAL1, PFKFB3, PFKP, PKM2, and LDHA expression, and RNA isolated for qPCR quantification of *Tnf-α*, *Il-6*, *Bmal1*, *Pfkfb3*, *Pfkp*, *Pkm*, and *Ldha* transcriptional changes.

*Histological study*

For H&E Staining and Analysis, lung tissues collected from mice of were fixed in 4% PFA for 24 h, embedded in paraffin, and sectioned at 5 μm thickness. Tissue sections were stained with H&E staining solution, followed by dehydration, clearing, and mounting with neutral resin. Histopathological evaluation (inflammatory infiltration, edema, necrosis) was performed under a light microscope.

Lung tissues harvested from mice were fixed in 4% paraformaldehyde (PFA) for precisely 24 h at 4°C, embedded in paraffin, and sectioned at 5 μm thickness. After deparaffinization, sections were stained with H&E staining solution for 1 min, then dehydrated in 95% and absolute ethanol, cleared in xylene, and mounted with neutral resin. Histopathological evaluation was performed under an Olympus IX73 microscope by two blinded pathologists using a semi-quantitative scoring system based on three pathological parameters: alveolar edema, hemorrhage, and inflammatory infiltration. Each scored from 0 to 4 using the following criteria: 0 indicated no or minimal abnormalities; 1 represented mild localized pathological changes; 2 denoted moderately severe but confined alterations; 3 signified either moderately extensive alterations or locally prominent pathological features; while 4 reflected widespread and profoundly severe manifestations. Lung injury score was calculated as the sum of individual parameters.

For Immunohistochemical (IHC) staining of lung tissues, paraffin-embedded lung tissue sections were deparaffinized, rehydrated, and subjected to antigen retrieval in citrate buffer (pH 6.0, 95°C, 20 min). Endogenous peroxidase and nonspecific binding were blocked with 3% H₂O₂ and 5% BSA, respectively. Sections were incubated overnight at 4°C with primary antibodies (BMAL1, 1:100), followed by HRP-conjugated goat anti-rabbit IgG (1:500) for 1 h at room temperature. Signals were developed with DAB chromogen, counterstained with hematoxylin, dehydrated, and mounted. Negative controls omitted primary antibodies. Stained sections were imaged under a light microscope (Olympus IX73), and positive staining areas (%) were quantified using Image-J software.

References

[1] R. Soncini, J. Vieira, A. C. Ramos Lopes, et al."Glucocorticoid receptor gene expression in a CLP-induced ARDS-like rat model treated with dexamethasone and metyrapone"*Mol Cell Endocrinol* (**2018)**, 474, 151. https://doi.org/10.1016/j.mce.2018.03.001

[2] D. Tang, M. Chen, X. Huang, et al."SRplot: A free online platform for data visualization and graphing"*PLoS One* (**2023)**, 18, e0294236. https://doi.org/10.1371/journal.pone.0294236

[3] Y. Oishi, S. Hayashi, T. Isagawa, et al."Bmal1 regulates inflammatory responses in macrophages by modulating enhancer RNA transcription"*Sci Rep* (**2017)**, 7, 7086. https://doi.org/10.1038/s41598-017-07100-3

[4] S. Mei, Q. Qin, Q. Wu, et al."Cistrome Data Browser: a data portal for ChIP-Seq and chromatin accessibility data in human and mouse"*Nucleic Acids Res* (**2017)**, 45, D658. https://doi.org/10.1093/nar/gkw983

[5] I. Rauluseviciute, R. Riudavets-Puig, R. Blanc-Mathieu, et al."JASPAR 2024: 20th anniversary of the open-access database of transcription factor binding profiles"*Nucleic Acids Res* (**2024)**, 52, D174. https://doi.org/10.1093/nar/gkad1059

[6] T. Tian, T. Zhang, S. Shi, et al."A dynamic DNA tetrahedron framework for active targeting"*Nat Protoc* (**2023)**, 18, 1028. https://doi.org/10.1038/s41596-022-00791-7


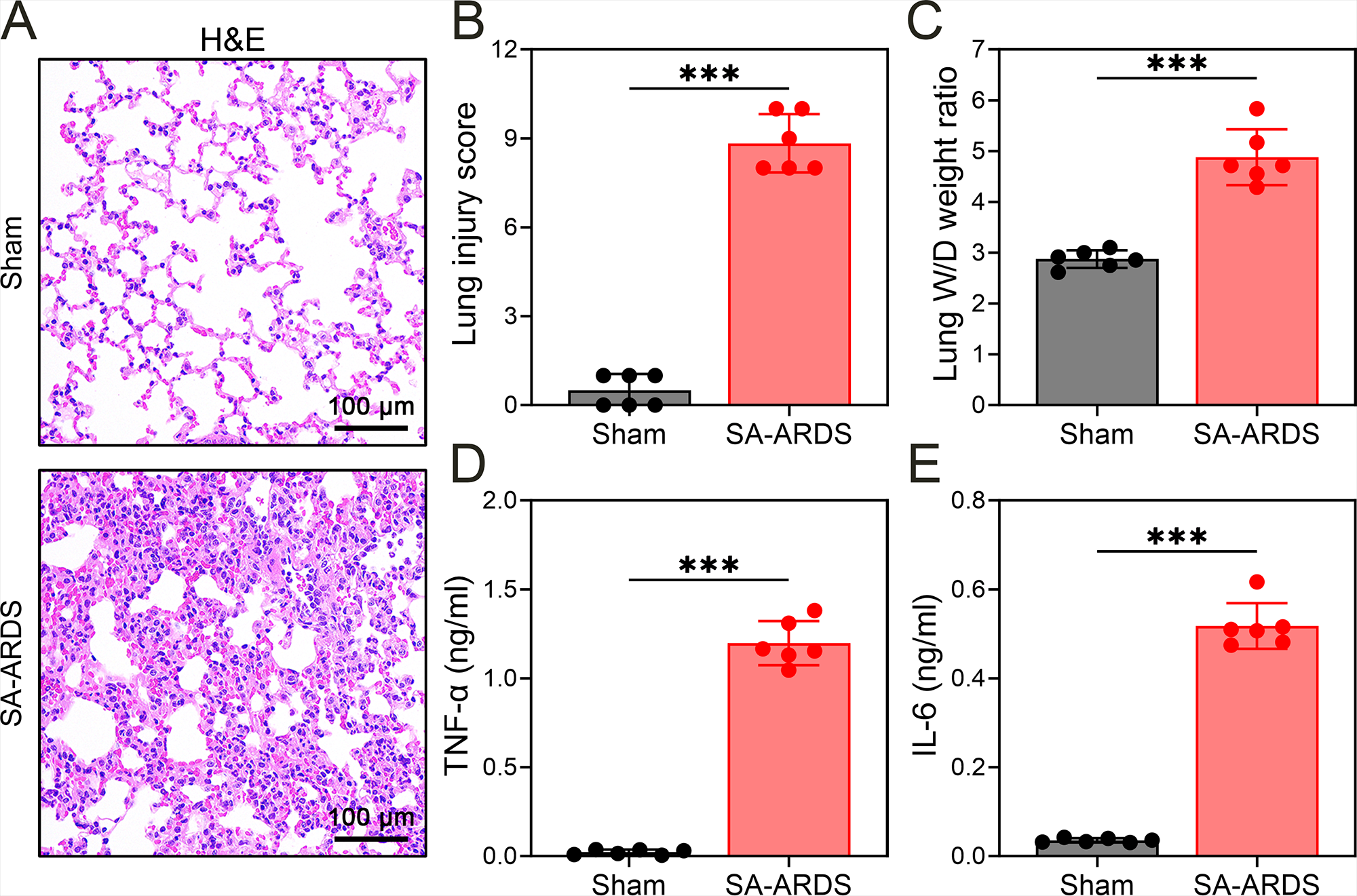


**Figure S1. Establishment of SA-ARDS mouse model.** (**A**) H&E staining analysis of lung tissues isolated from sham or SA-ARDS mice (*n* = 6). (**B**) Statistics of lung injury scores of lung tissues isolated from sham or SA-ARDS mice (*n* = 6). (**C**) Pulmonary edema assessment by wet/dry weight ratio of lung tissues isolated from sham or SA-ARDS mice (*n* = 6). (**D, E**) TNF-α levels and IL-6 levels in BALF of sham or SA-ARDS mice. Quantitative data are presented as mean ± standard deviation (SD). Statistical significance between two groups was determined by independent samples *t*-test. Significance levels: ****P* < 0.001.


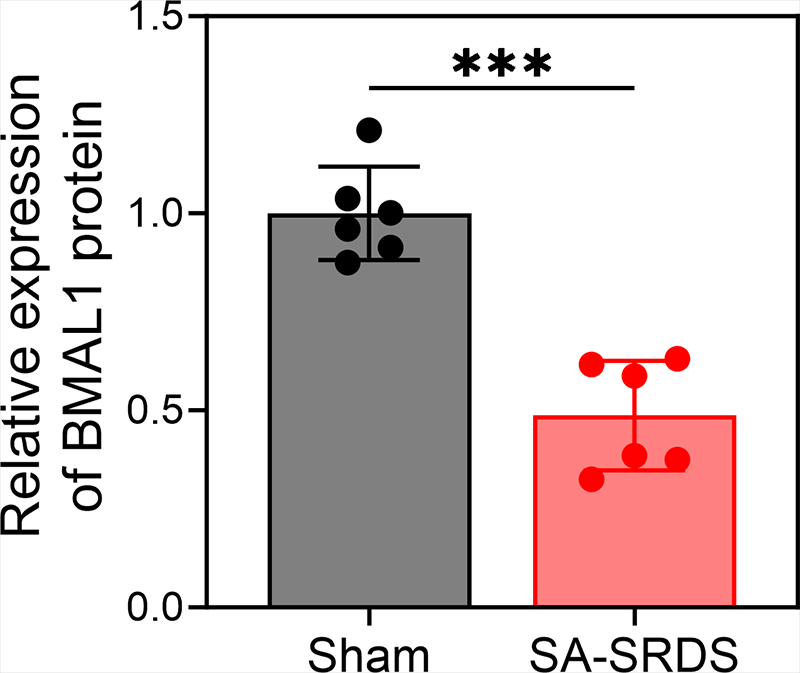


**Figure S2.** Comparison of relative expression levels of BMAL1 in lung tissues of sham and SA-ARDS mice (*n* = 6). Data are presented as means ± SD. Statistical significance between two groups was determined by independent samples *t*-test. Significance levels: ****P* < 0.001.


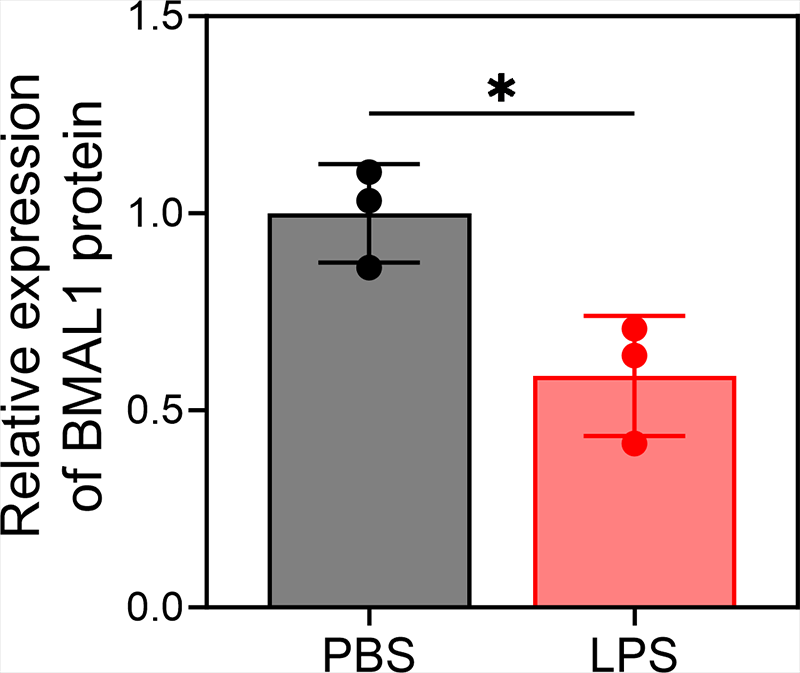


**Figure S3.** Comparison of relative expression levels of BMAL1 in MH-S cells before and after LPS (1 μg·mL⁻¹, 24 h) stimulation (*n* = 3). Data are presented as means ± SD. Statistical significance between two groups was determined by independent samples *t*-test. Significance levels: **P* < 0.05.


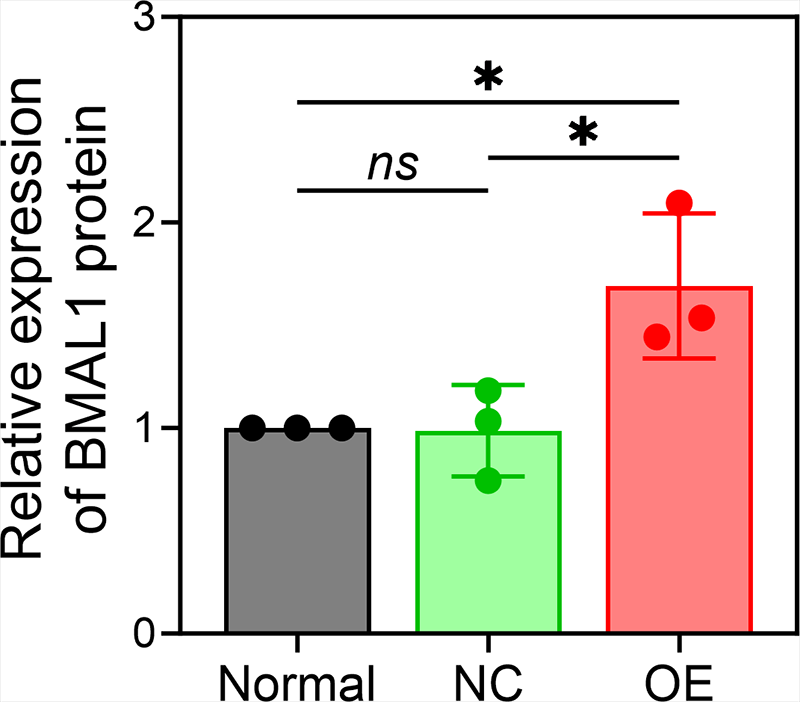


**Figure S4.** Comparison of relative expression levels of BMAL1 in normal MH-S cells (Normal), MH-S cells transfected with negative control plasmid (NC), BMAL1-overexpressing MH-S cells (OE) (*n* = 3). Data are presented as means ± SD. Statistical significance among three or more groups were analyzed using one-way analysis of variance (ANOVA). Significance levels: **P* < 0.05, *ns*: no significant difference.


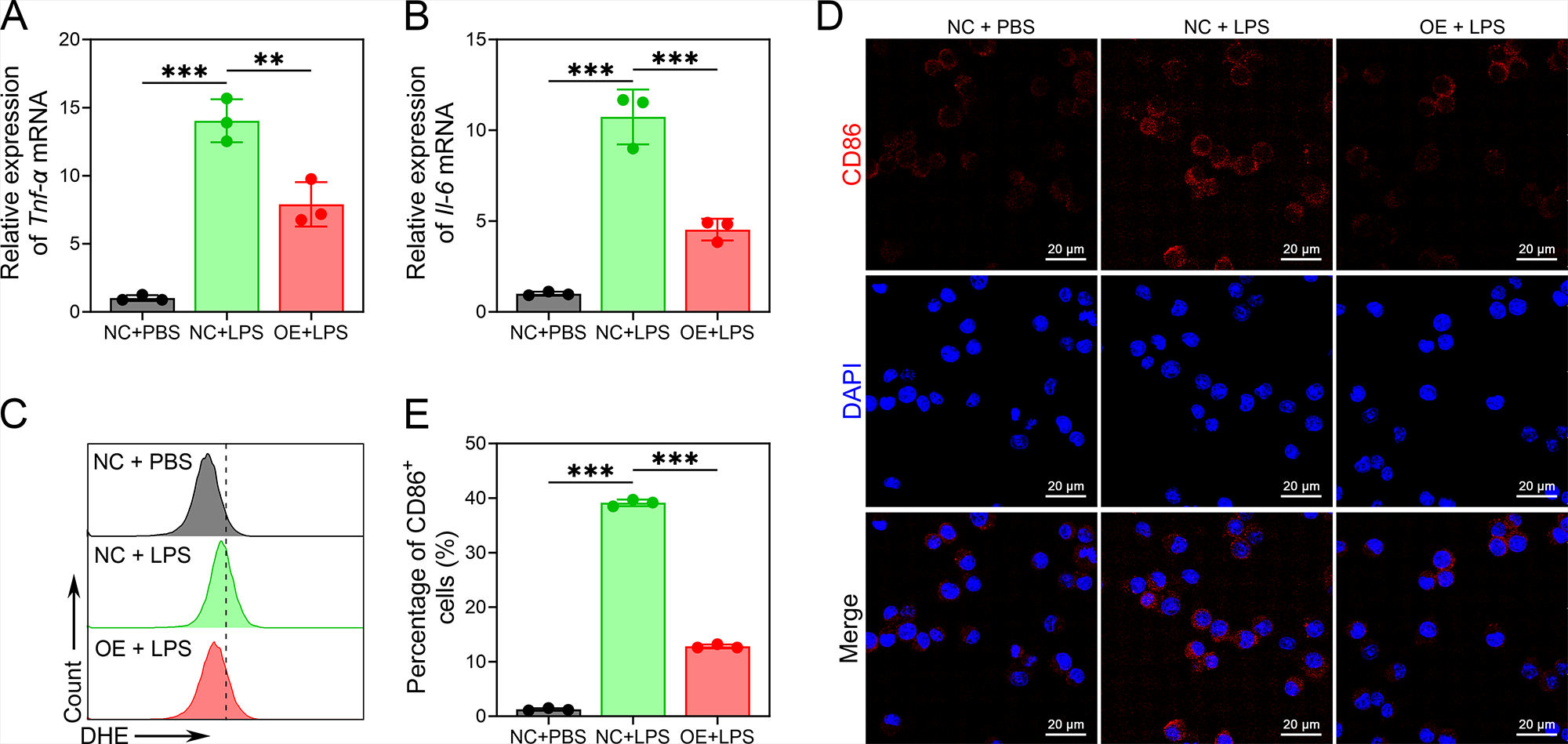


**Figure S5.** **Critical role of BMAL1 in SA-ARDS.** (**A, B**) Comparison of relative expression levels of *Tnf-α* and *Il-6* (*n* = 3). Experimental Groups. **NC + PBS** (Normal control MH-S cells with PBS); **NC + LPS** (Normal control MH-S cells with LPS (1 μg/mL, 24 h) challenge); **OE + LPS** (BMAL1-overexpressing MH-S cells with LPS (1 μg/mL, 24h) challenge). (**C**) Flow cytometric quantification of DHE-stained intracellular ROS of different groups. (**D**) Immunofluorescence staining for CD86 in MH-S cells of different groups (*n* = 3). (**E**) Flow cytometric quantification of CD86 positive cells of different groups (*n* = 3). Quantitative data are presented as mean ± SD. Statistical significance among three or more groups were analyzed using one-way ANOVA. Significance levels: ***P* < 0.01, ****P* < 0.001.


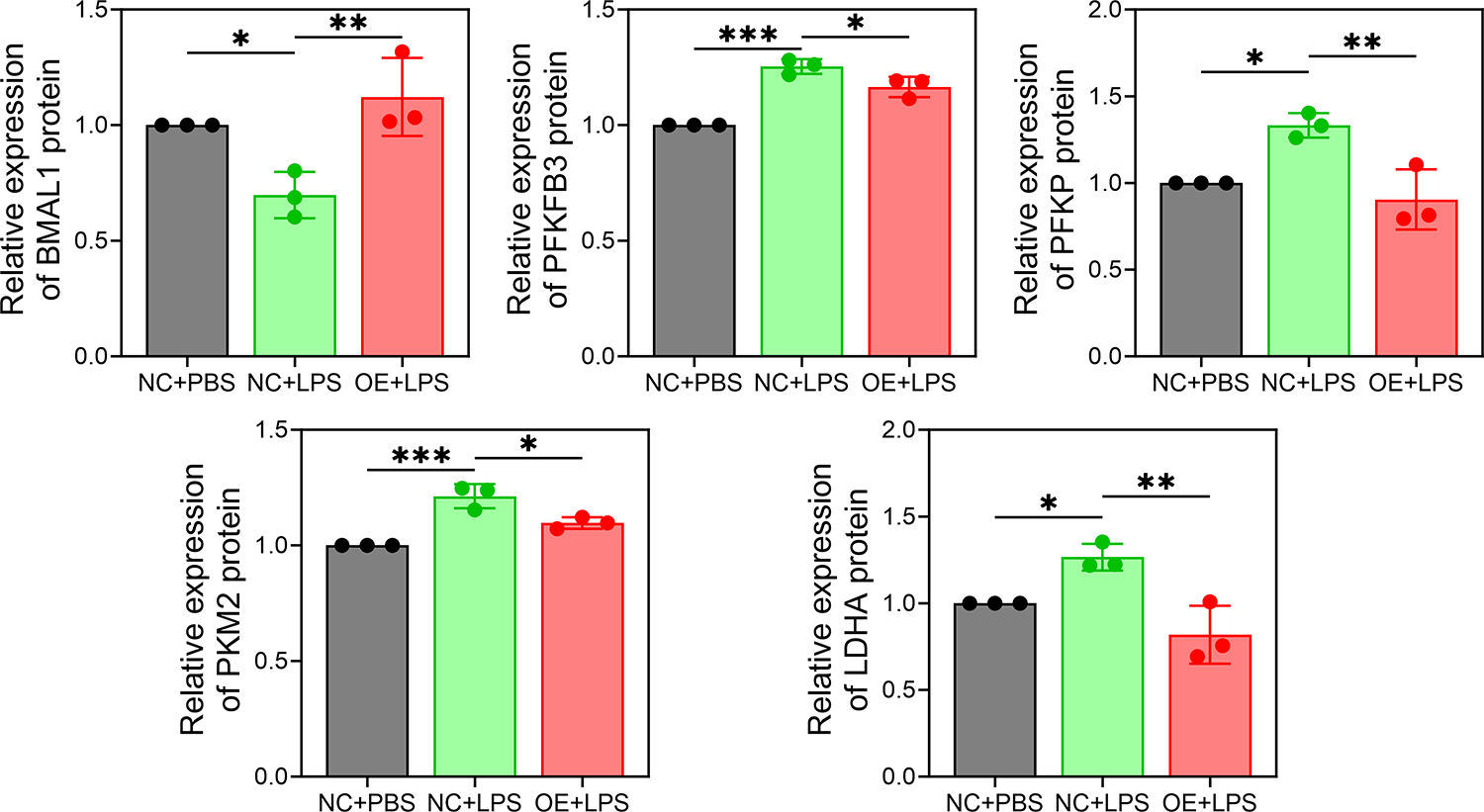


**Figure S6.** Comparison of relative expression levels of BMAL1, PFKFB3, PFKP, PKM2, and LDHA (*n* = 3). Data are presented as means ± SD. Statistical significance among three or more groups were analyzed using one-way ANOVA. Significance levels: **P* < 0.05, ***P* < 0.01, ****P* < 0.001.


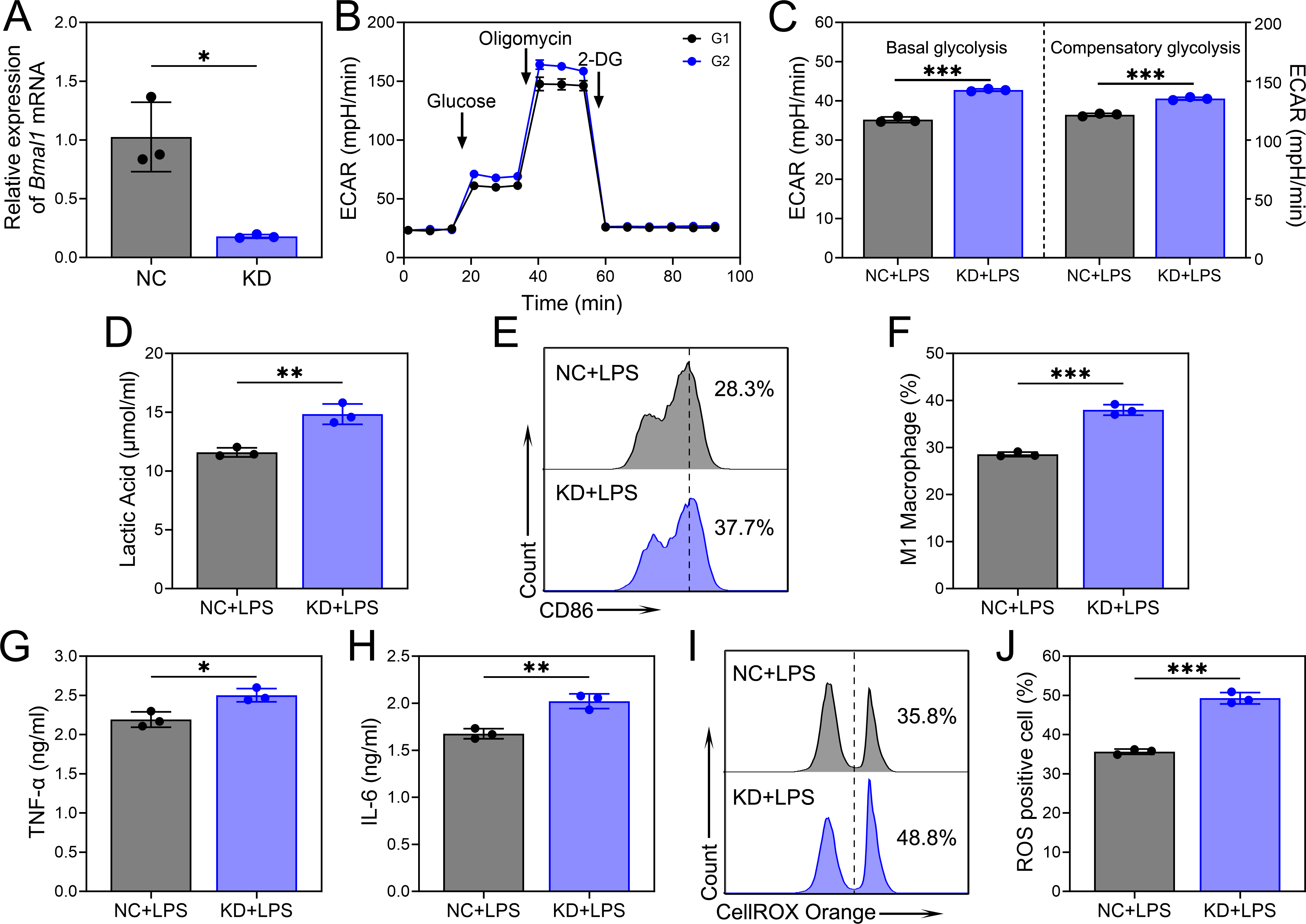


**Figure S7. *In vitro* effects of BMAL1 knockdown in MH-S cells. Treatment: G1**: NC + LPS (NC MH-S cells treated with LPS (1 μg·mL⁻¹) for 24 h); **G2**: KD + LPS (BMAL1-KD MH-S cells treated with LPS (1 μg·mL⁻¹) for 24 h). **(A)** qPCR validation of *Bmal1* mRNA levels (*n* = 3). **(B, C)** Basal glycolysis and compensatory glycolysis parameters of MH-S cells after different treatments, as determined by Seahorse XF Analyzer-mediated ECAR measurement (*n* = 3). **(D)** Comparison of concentrations of lactic acid secreted from MH-S cells after different treatments (*n* = 3). **(E, F)** Representative flow cytometry histograms and quantitative analysis (*n* = 3) of the CD86 expression in MH-S macrophages upon different treatments. **(G, H)** Comparison of concentrations of TNF-α and IL-6 secreted from MH-S cells upon different treatments (*n* = 3). **(I, J)** Representative flow cytometry histograms and quantitative analysis (*n* = 3) of intracellular ROS levels in MH-S macrophages stained with CellROX Orange across different treatments. Quantitative data are presented as mean ± SD. Statistical significance was analyzed using Student’s t-test. Significance levels: **P* < 0.05, ***P* < 0.01, ****P* < 0.001.


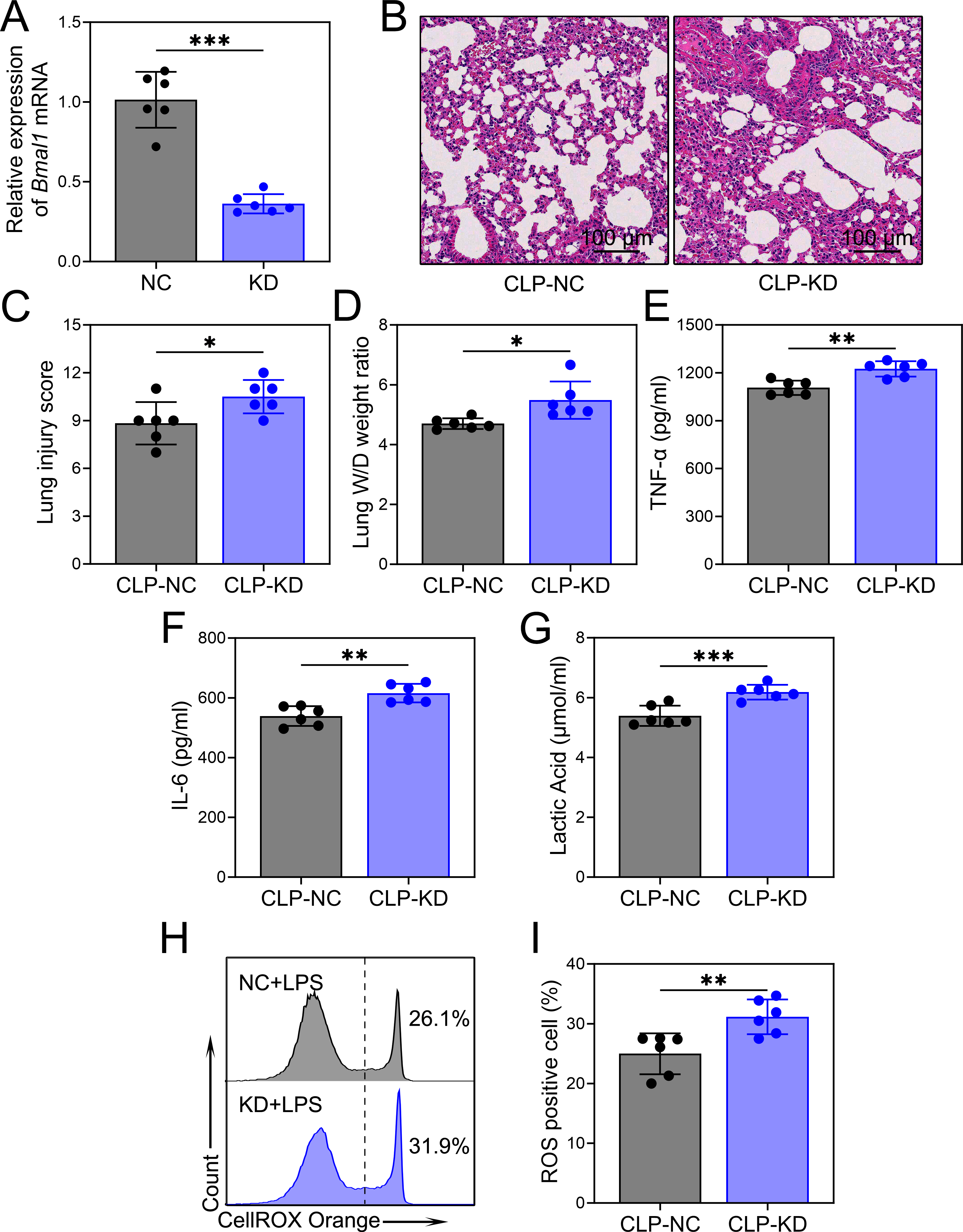


**Figure S8. *In vivo* effects of macrophage-specific BMAL1 knockdown. Treatments:** **G1**: CLP-NC (AAV-NC-pretreated mice subjected to CLP); **G2**: CLP-KD (AAV-shBMAL1-pretreated mice subjected to CLP). (**A**) qPCR of *Bmal1* in isolated AMs (*n* = 6). (**B**) Representative H&E staining images of lung tissues from mice after different treatments. (**C**) Quantitative analysis of the lung injury scores based on H&E pathology (*n* = 6). (**D**) Lung wet-to-dry (W/D) weight ratios of mice after different treatments (*n* = 6). (**E-G**) Comparison of concentrations of TNF-α, IL-6, and lactic acid in the BALF of mice upon different treatments (*n* = 6). (**H, I**) Representative flow cytometry histograms and quantitative analysis (*n* = 6) of intracellular ROS levels in lung single-cell suspensions stained with CellROX Orange across different treatments. Quantitative data are presented as mean ± SD. Statistical significance was analyzed using Student’s t-test. Significance levels: **P* < 0.05, ***P* < 0.01, ****P* < 0.001.


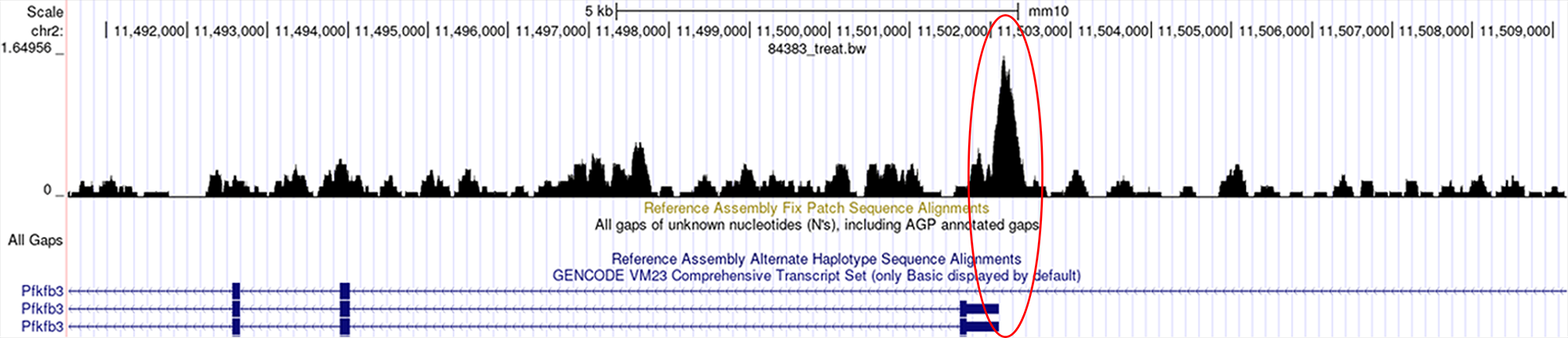


**Figure S9.** Genomic visualization of BMAL1 binding peaks from ChIP-seq analysis.


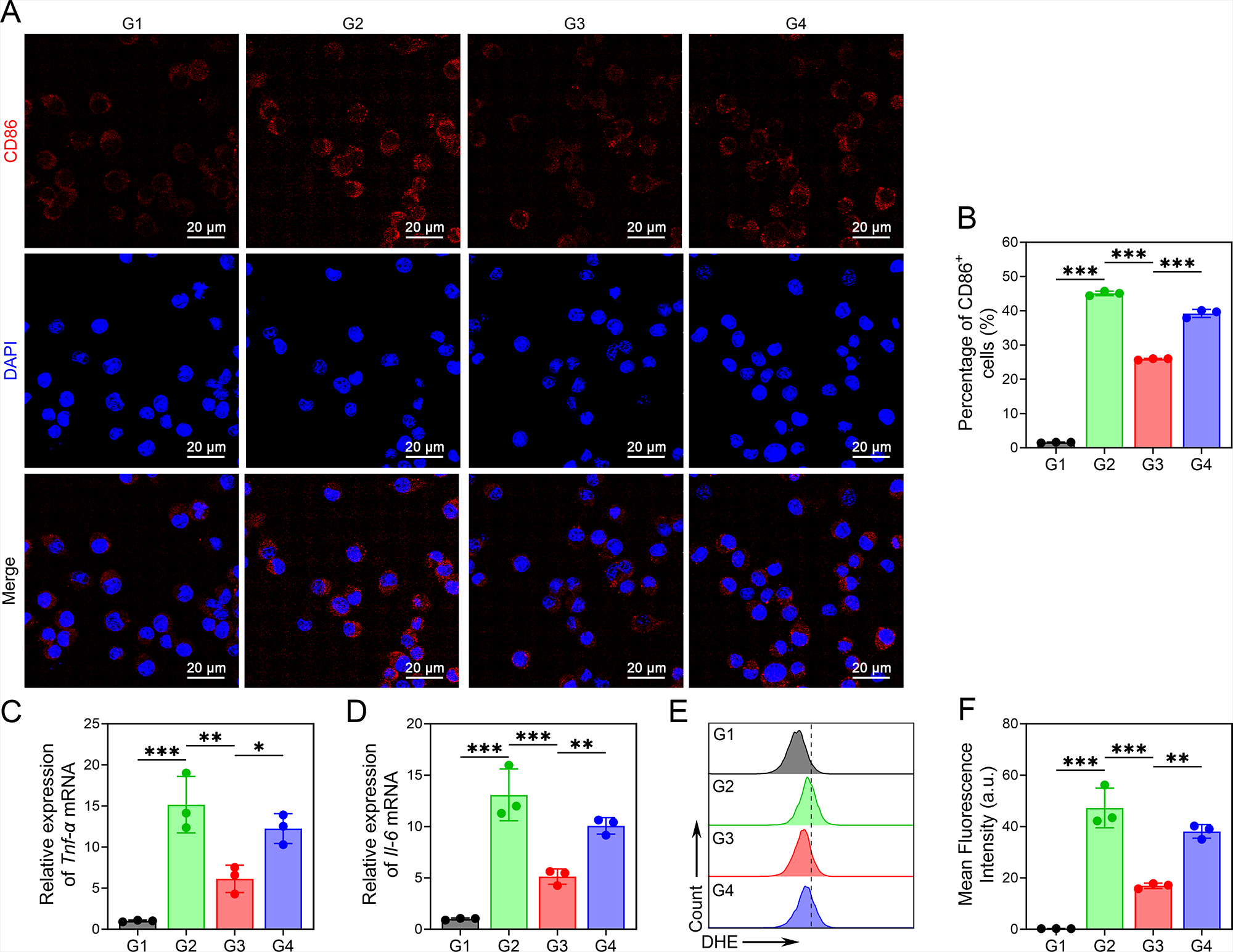


**Figure S10. BMAL1-mediated glycolytic control through PFKFB3 modulates macrophage polarization and inflammation in LPS-stimulated AMs *in vitro*.** (**A**) Immunofluorescence staining for CD86 in MH-S cells of different groups (*n* = 3). Experimental Groups. **G1**: NC + PBS (Normal control MH-S cells with PBS); **G2**: NC + LPS (Normal control MH-S cells with LPS (1 μg·mL⁻¹, 24 h) challenge); **G3**: OE + LPS (BMAL1-overexpressing MH-S cells with LPS (1 μg·mL⁻¹, 24 h) challenge); **G4**: OE + LPS + PFKFB3 (BMAL1-overexpressing MH-S cells with LPS (1 μg·mL⁻¹, 24 h) challenge and PFKFB3 plasmid transfection). (**B**) Flow cytometric quantification of CD86 positive cells of different groups indicated in **A** (*n* = 3). (**C, D**) Comparison of relative expression levels of *Tnf-α* and *Il-6* of different groups indicated in **A** (*n* = 3). (**E**) Flow cytometric histograms of DHE-stained intracellular ROS of different groups indicated in **A. (F)**. Statistical quantification of the MFI for DHE staining presented in **Figure 3H** (*n* = 3) Quantitative data are presented as mean ± SD. Statistical significance was calculated *via* ordinary one-way ANOVA. Significance levels: **P* < 0.05, ***P* < 0.01, ****P* < 0.001.


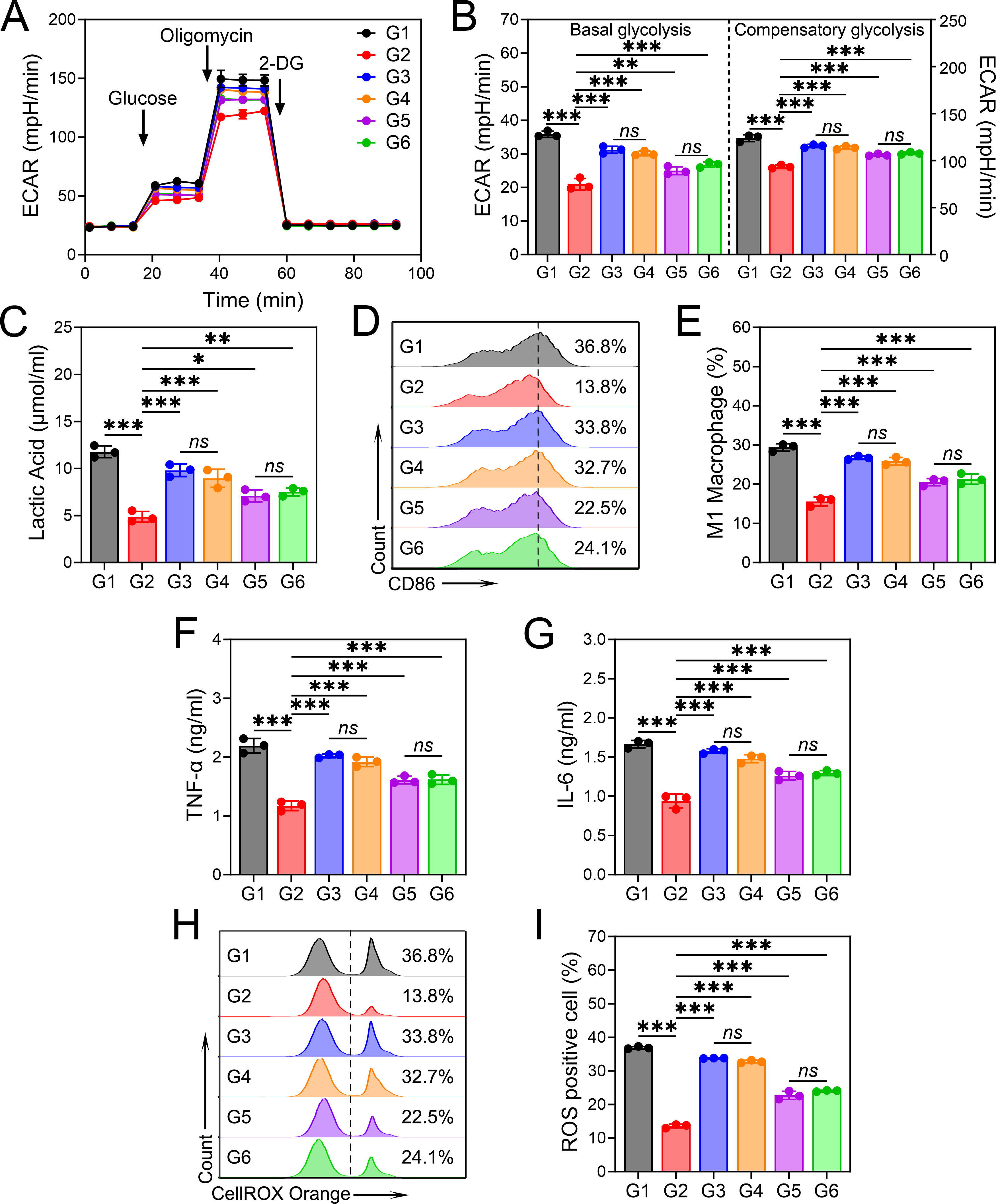


**Figure S11. Rescue effects of multiple glycolytic enzymes *in vitro*.** **Treatments:** **G1**: NC + LPS (NC MH-S cells treated with 1 μg·mL⁻¹ LPS for 24 h); **G2**: OE + LPS (BMAL1-OE MH-S cells treated with 1 μg·mL⁻¹ LPS for 24 h); **G3**: OE + LPS + PFKFB3-OE (BMAL1-OE MH-S cells transfected with the PFKFB3-OE plasmid treated with 1 μg·mL⁻¹ LPS for 24 h); **G4**: OE + LPS + PFKP-OE (BMAL1-OE MH-S cells transfected with the PFKP-OE plasmid treated with 1 μg·mL⁻¹ LPS for 24 h); **G5**: OE + LPS + PKM2-OE (BMAL1-OE MH-S cells transfected with the PKM2-OE plasmid treated with 1 μg·mL⁻¹ LPS for 24 h); G7: OE + LPS + LDHA-OE (BMAL1-OE MH-S cells transfected with the LDHA-OE plasmid treated with 1 μg·mL⁻¹ LPS for 24 h). **(A, B)** Basal glycolysis and compensatory glycolysis parameters of MH-S cells after different treatments, as determined by Seahorse XF Analyzer-mediated ECAR measurement (*n* = 3). **(C)** Comparison of concentrations of lactic acid secreted from MH-S cells after different treatments (*n* = 3). **(D, E)** Representative flow cytometry histograms and quantitative analysis (*n* = 3) of the CD86 expression in MH-S macrophages upon different treatments. **(F, G)** Comparison of concentrations of TNF-α and IL-6 secreted from MH-S cells upon different treatments (*n* = 3). **(H, I)** Representative flow cytometry histograms and quantitative analysis (*n* = 3) of intracellular ROS levels in MH-S macrophages stained with CellROX Orange across different treatments. Quantitative data are presented as mean ± SD. Statistical significance was calculated *via* ordinary one-way ANOVA. Significance levels: **P* < 0.05 ***P* < 0.01, ****P* < 0.001, *ns*: no significant difference.


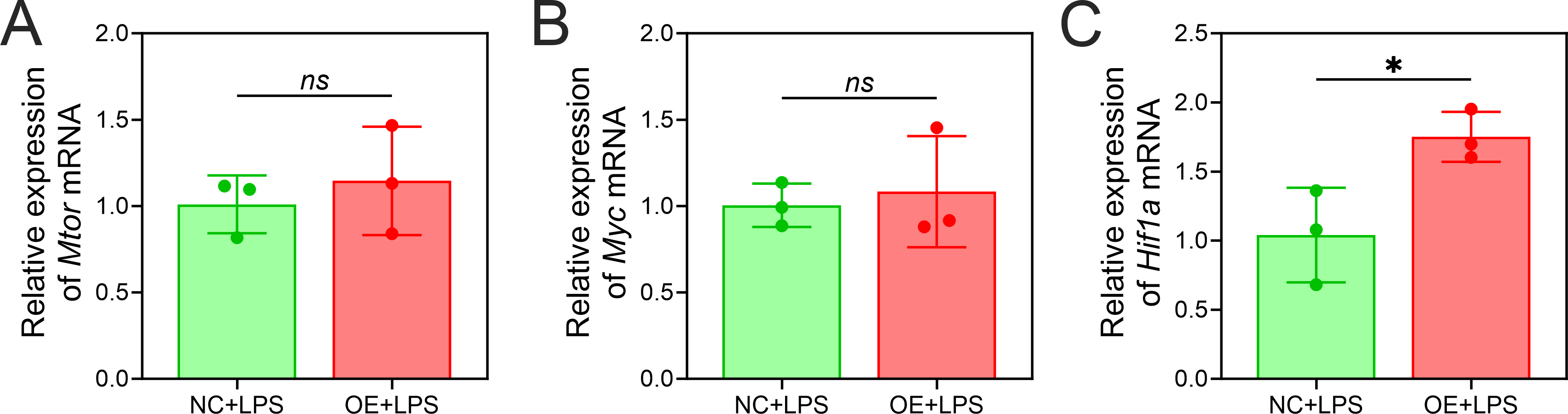


**Figure S12. Expression analysis of alternative glycolytic regulators in MH-S cells. (A-C)** mRNA expression levels of *Mtor*, *Myc*, and *Hif1a* in LPS-induced MH-S cells following BMAL1 overexpression, as determined by qPCR (*n* = 3). Data are presented as mean ± SD. Statistical significance was analyzed using Student’s t-test. Significance levels: **P* < 0.05, *ns*: not significant.


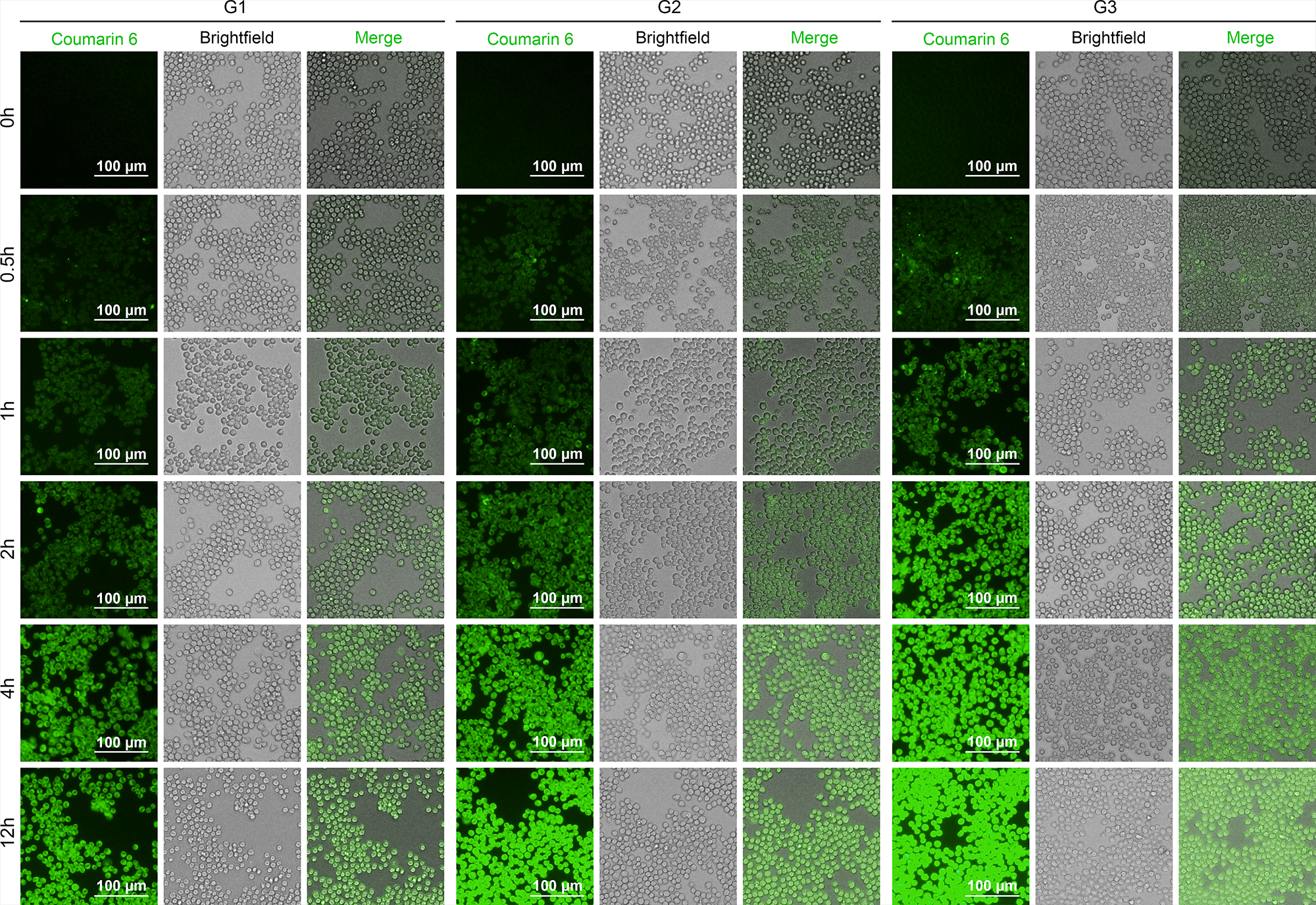


**Figure S13.** Fluorescence microscopic imaging of coumarin-6 (G1, 16 μM), TC (G2, 16 μM based on the loaded Nob), and TCT (G3, 16 μM based on the loaded Nob) uptake in MH-S cells over time.


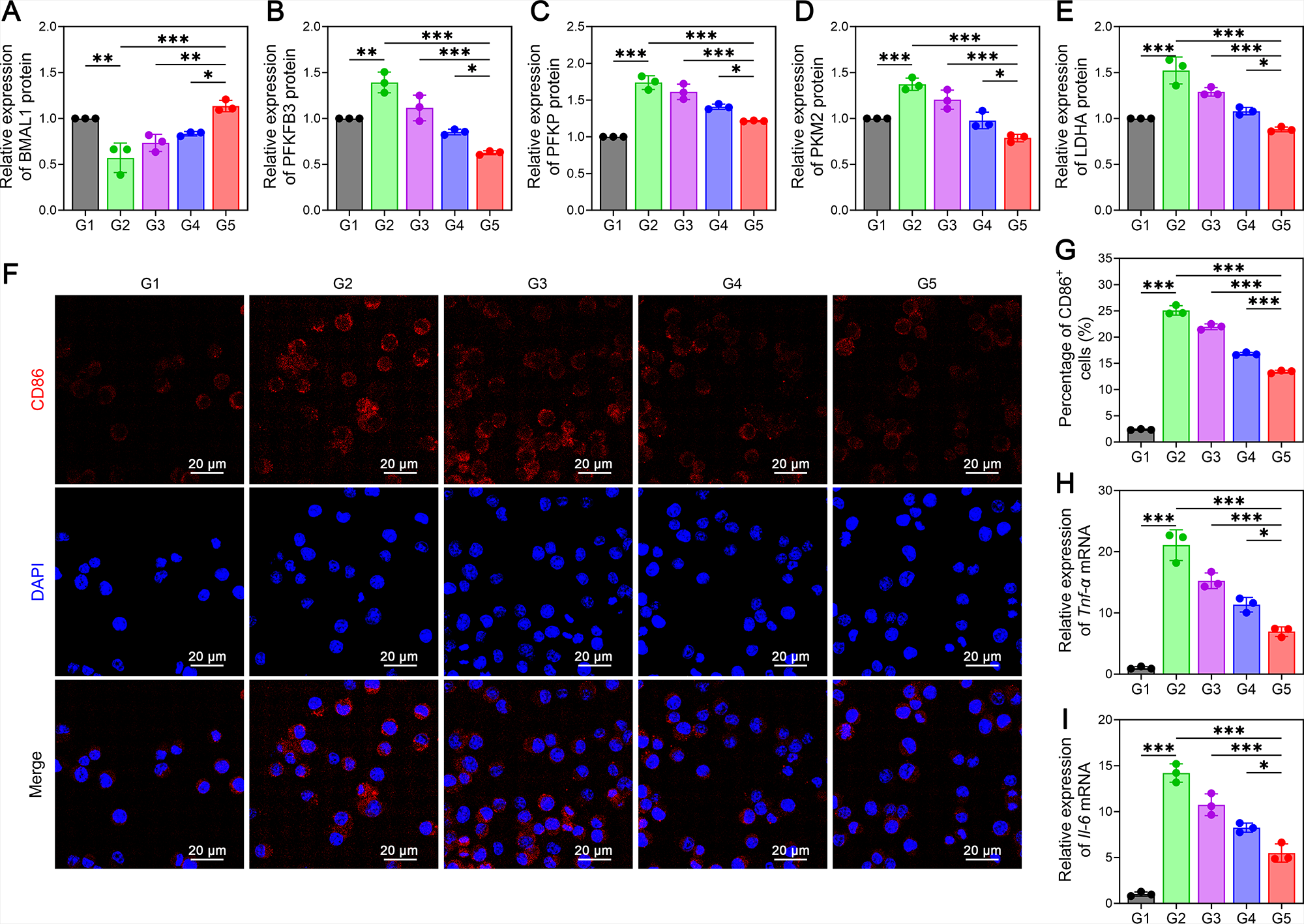


**Figure S14.** **Therapeutic efficacy of TNT in LPS-stimulated AMs *in vitro*.** (**A-E**) Comparison of relative expression levels of BMAL1, PFKFB3, PFKP, PKM2, and LDHA of different groups indicated (*n* = 3). **Experimental Groups. G1**: PBS (MH-S cells with PBS); **G2**: LPS (MH-S cells with LPS (1 μg·mL⁻¹, 24 h) challenge); **G3**: LPS + Nob (MH-S cells with LPS (1 μg·mL⁻¹, 24 h) challenge and Nob (16 μΜ, 23 h, calculated as Nob equivalent) treatment); **G4**: LPS + TN (MH-S cells with LPS (1 μg·mL⁻¹, 24 h) challenge and TN (16 μΜ, 23 h, calculated as Nob equivalent) treatment); **G5**: LPS + TNT (MH-S cells with LPS (1 μg·mL⁻¹, 24 h) challenge and TNT (16 μΜ, 23 h, calculated as Nob equivalent) treatment). (**F**) Immunofluorescence staining for CD86 in MH-S cells of different groups indicated in **A-E** (*n* = 3). (**G**) Flow cytometric quantification of CD86 positive cells of different groups indicated in **A-E** (*n* = 3). (**H-I**) Comparison of relative expression levels of *Tnf-α* and *Il-6* of different groups indicated in **A-E** (*n* = 3). Statistical significance was calculated *via* ordinary one-way ANOVA. Significance levels: **P* < 0.05, ***P* < 0.01, ****P* < 0.001.


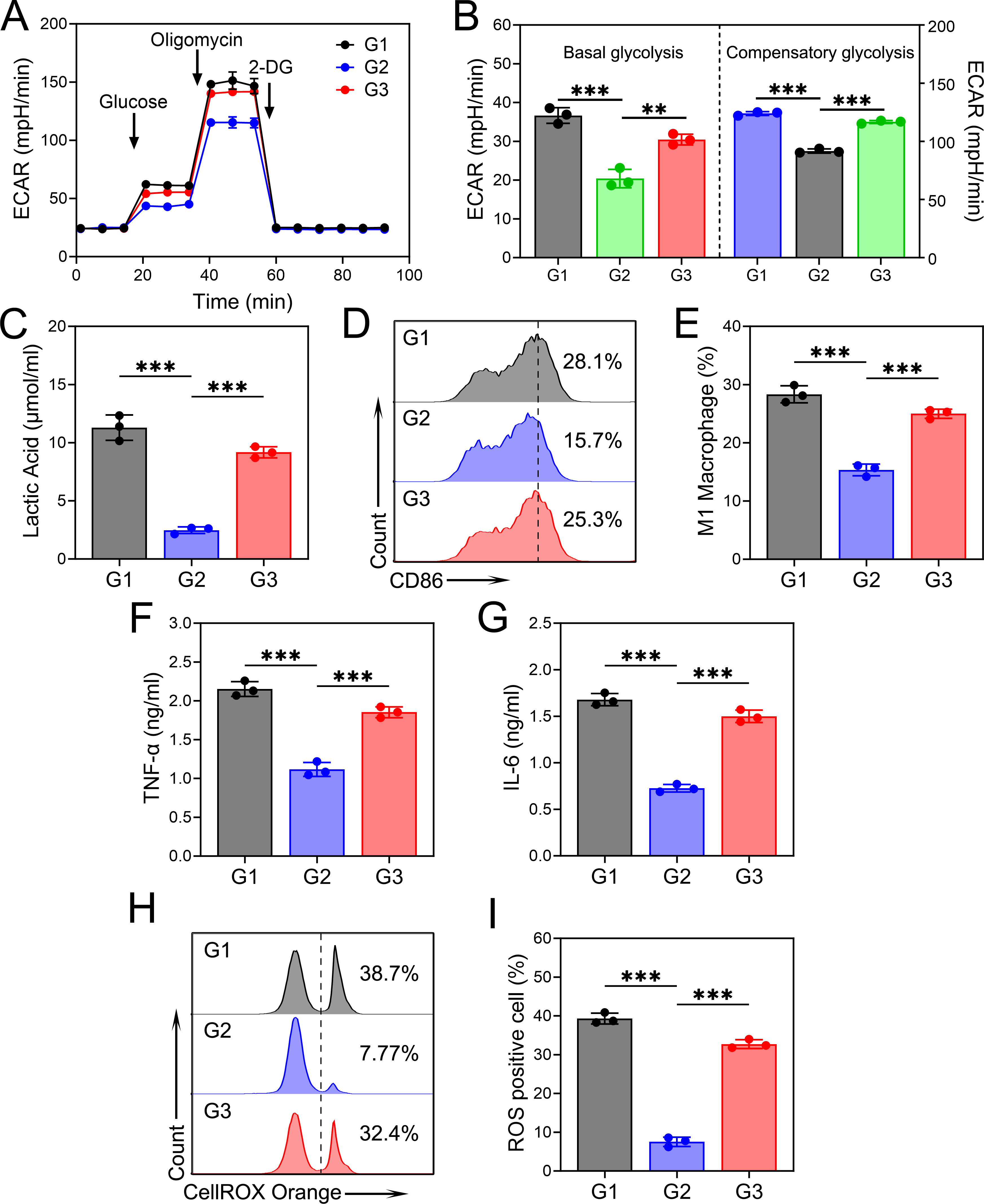


**Figure S15. The *in vitro* therapeutic efficacy of TNT is dependent on BMAL1.** **Treatments: G1**: AAV-NC-transfected MH-S cells treated with LPS (1 μg·mL⁻¹) for 24 h; **G2**: AAV-NC-transfected MH-S cells treated with LPS (1 μg·mL⁻¹) and TNT (16 μΜ based on Nob) for 24 h; **G3**: AAV-shBMAL1-transfected MH-S cells treated with LPS (1 μg·mL⁻¹) and TNT (16 μΜ based on Nob) for 24 h. **(A, B)** Basal glycolysis and compensatory glycolysis parameters of MH-S cells after different treatments, as determined by Seahorse XF Analyzer-mediated ECAR measurement (*n* = 3). **(C)** Comparison of concentrations of lactic acid secreted from MH-S cells after different treatments (*n* = 3). **(D, E)** Representative flow cytometry histograms and quantitative analysis (*n* = 3) of the CD86 expression in MH-S macrophages upon different treatments. **(F, G)** Comparison of concentrations of TNF-α and IL-6 secreted from MH-S cells upon different treatments (*n* = 3). **(H, I)** Representative flow cytometry histograms and quantitative analysis (*n* = 3) of intracellular ROS levels in MH-S macrophages stained with CellROX Orange across different treatments. Quantitative data are presented as mean ± SD. Statistical significance was calculated *via* ordinary one-way ANOVA. Significance levels: ***P* < 0.01, ****P* < 0.001.


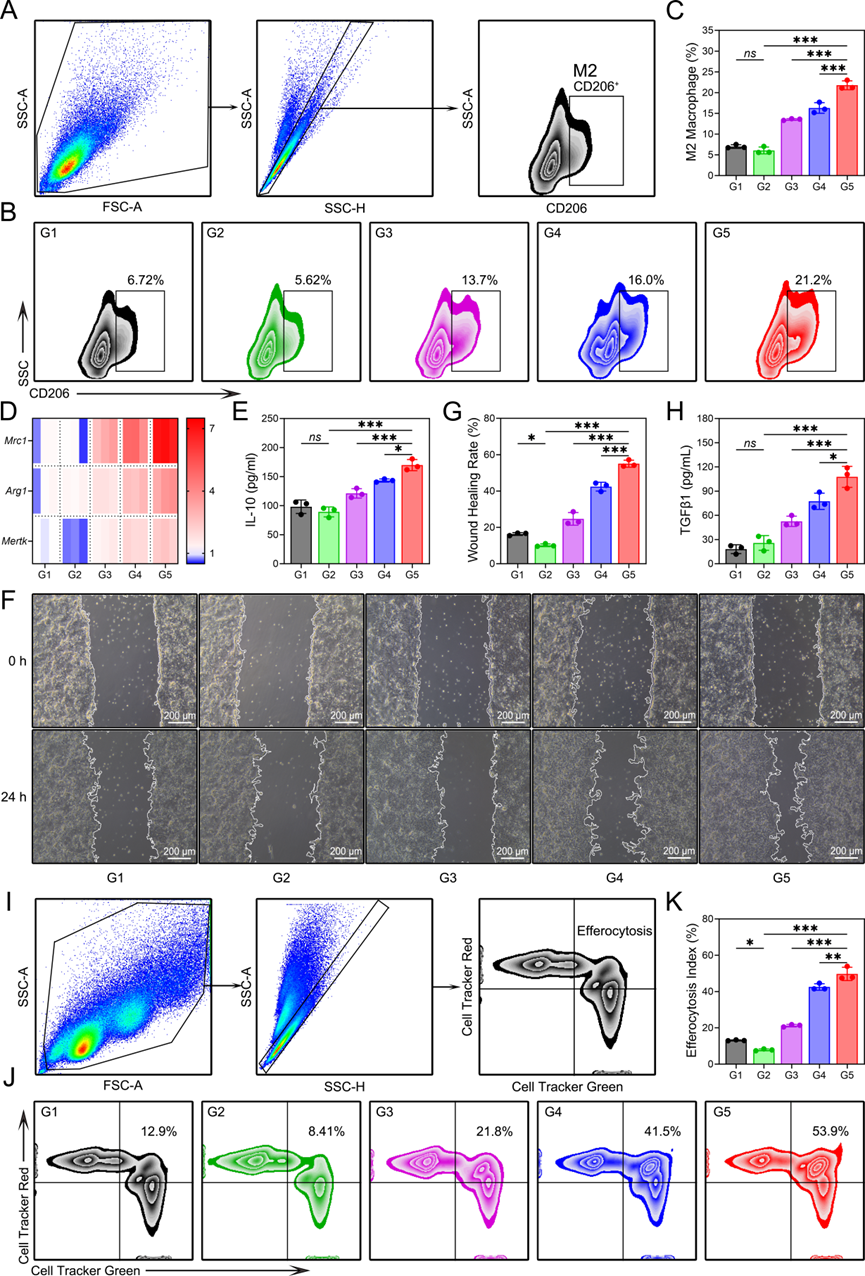


**Figure S16. The TNT nanoplatform actively promotes M2 polarization and enhances the tissue repair and clearance functions of LPS-stimulated MH-S macrophages *in vitro*.** **Treatments: G1**: MH-S cells treated with PBS for 24 h; **G2**: MH-S cells treated with LPS (1 μg·mL⁻¹) for 24 h; **G3**: MH-S cells treated with LPS (1 μg·mL⁻¹) and Nob (16 μΜ) for 24 h; **G4**: MH-S cells treated with LPS (1 μg·mL⁻¹) and TN (16 μΜ based on Nob) for 24 h; **G5**: MH-S cells treated with LPS (1 μg·mL⁻¹) and TNT (16 μΜ based on Nob) for 24 h. **(A)** Flow cytometry gating strategy for identifying the expression of the M2 marker CD206 on MH-S cells. **(B)** Representative flow cytometry scatter plots of surface CD206 expression in different treatment groups. **(C)** Quantitative analysis of the proportion of M2 macrophages (CD206⁺ cells) (*n* = 3). **(D)** Heatmap illustrating the relative mRNA expression levels of M2 polarization and function-associated genes (*Mrc1*, *Arg1*, and *Mertk*) assessed by RT-qPCR (*n* = 3). **(E)** Secretion levels of the anti-inflammatory cytokine IL-10 in the MH-S culture supernatants, measured by ELISA (*n* = 3). **(F)** Representative images of the MLE-12 alveolar epithelial cell scratch wound-healing assay (at 0 h and 24 h) following treatment with conditioned medium from different groups of MH-S cells in a Transwell co-culture system (*n* = 3). **(G)** Quantitative analysis of the wound closure rate (%) for MLE-12 cells (*n* = 3). **(H)** Secretion levels of the repair-associated factor TGF-β1 in the MH-S culture supernatants, measured by ELISA (*n* = 3). **(I)** Flow cytometry gating strategy for evaluating the *in vitro* phagocytosis of apoptotic cells (efferocytosis) by MH-S macrophages. **(J)** Representative flow cytometry scatter plots showing the efferocytosis of fluorescently labeled apoptotic cells by treated MH-S macrophages. **(K)** Quantitative analysis of the efferocytosis index across different groups (*n* = 3). Quantitative data are presented as mean ± SD. Statistical significance was calculated *via* ordinary one-way ANOVA. Significance levels: **P* < 0.05, ***P* < 0.01, ****P* < 0.001, *ns*: no significant difference.


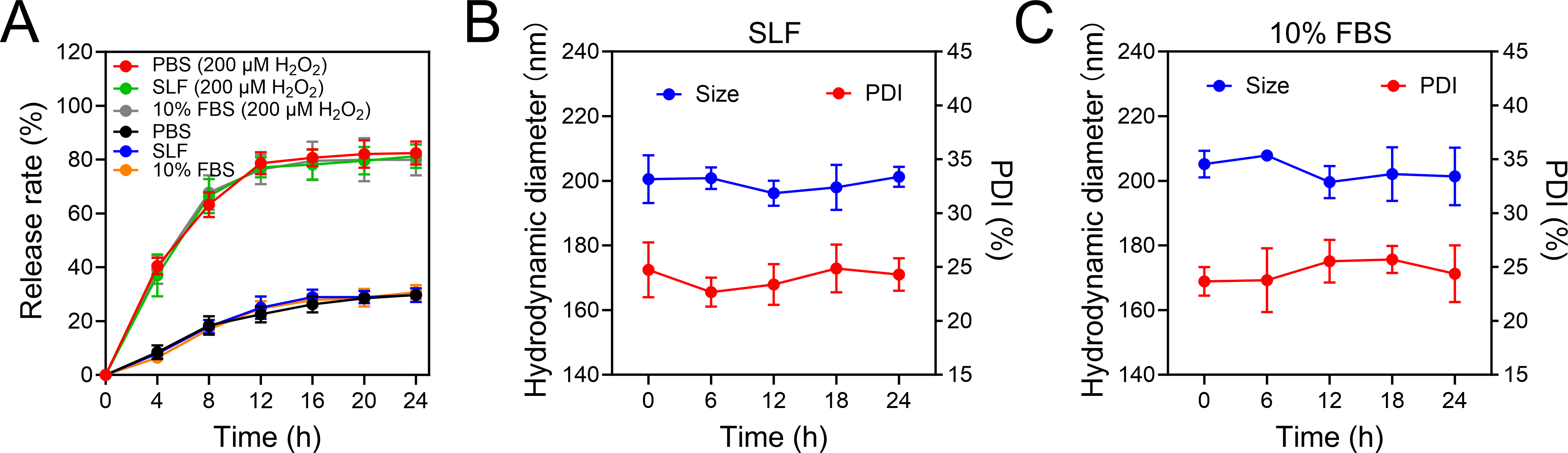


**Figure S17. *In vitro* ROS-responsive release and structural stability of RM@TNT in physiologically relevant fluid environments.** **(A)** Cumulative TNT release profiles from RM@TNT in PBS, SLF, and 10% FBS in the presence or absence of 200 μM H₂O₂. **(B, C)** Time-dependent changes in the hydrodynamic diameter and PDI of RM@TNT incubated in SLF and 10% FBS over a 24-hour period (measured at 0, 6, 12, 18, and 24 h). Data are presented as mean ± SD.


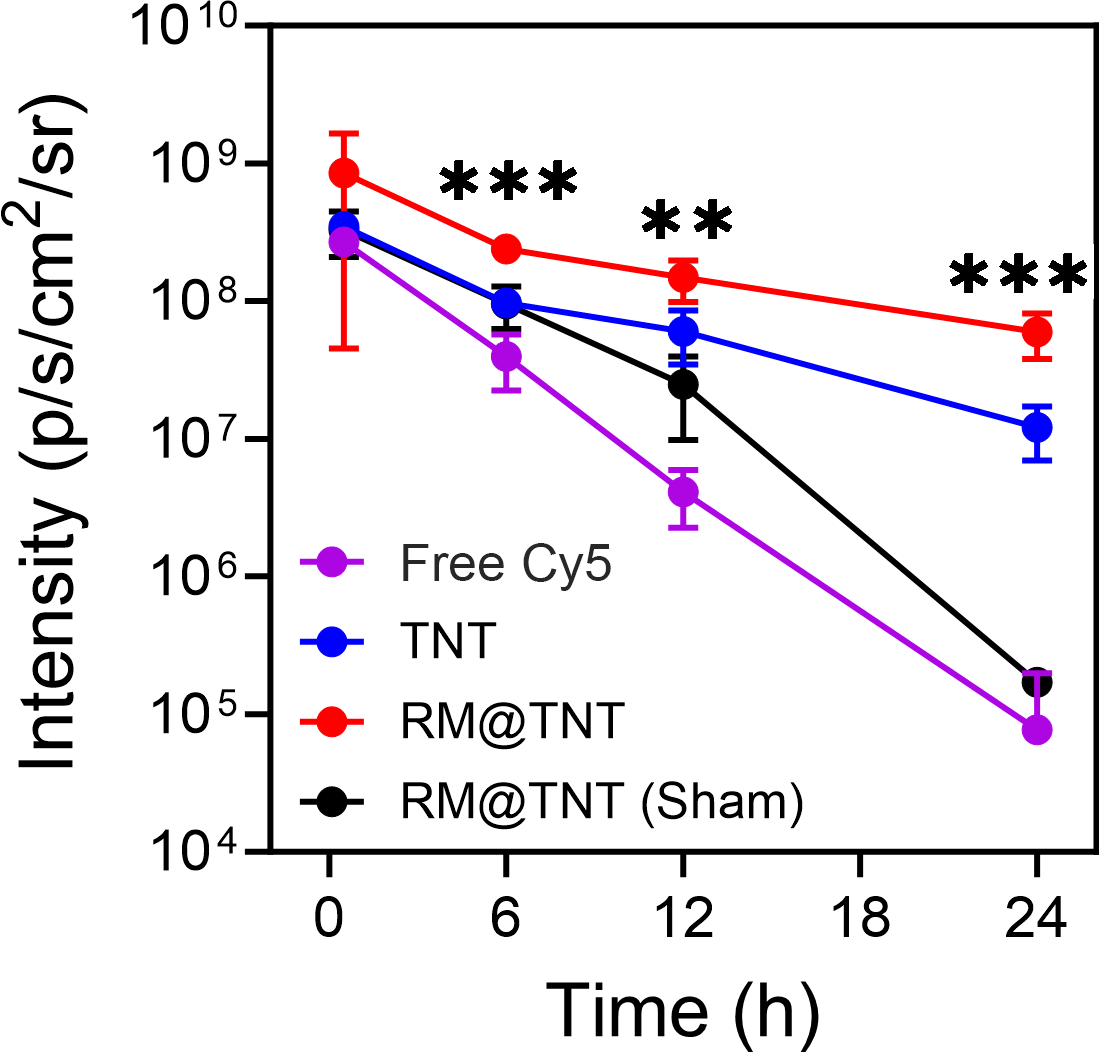


**Figure S18.** Quantitative analysis of pulmonary retention kinetics for intranasally administered FreeCy5, Cy5-labeled TNT, and Cy5-labeled RM@TNT in SA-ARDS model mice or sham-operated mice (*n* = 3). Statistical significance was calculated *via* ordinary one-way ANOVA. Significance levels: **P* < 0.05, ***P* < 0.01, ****P* < 0.001.

*
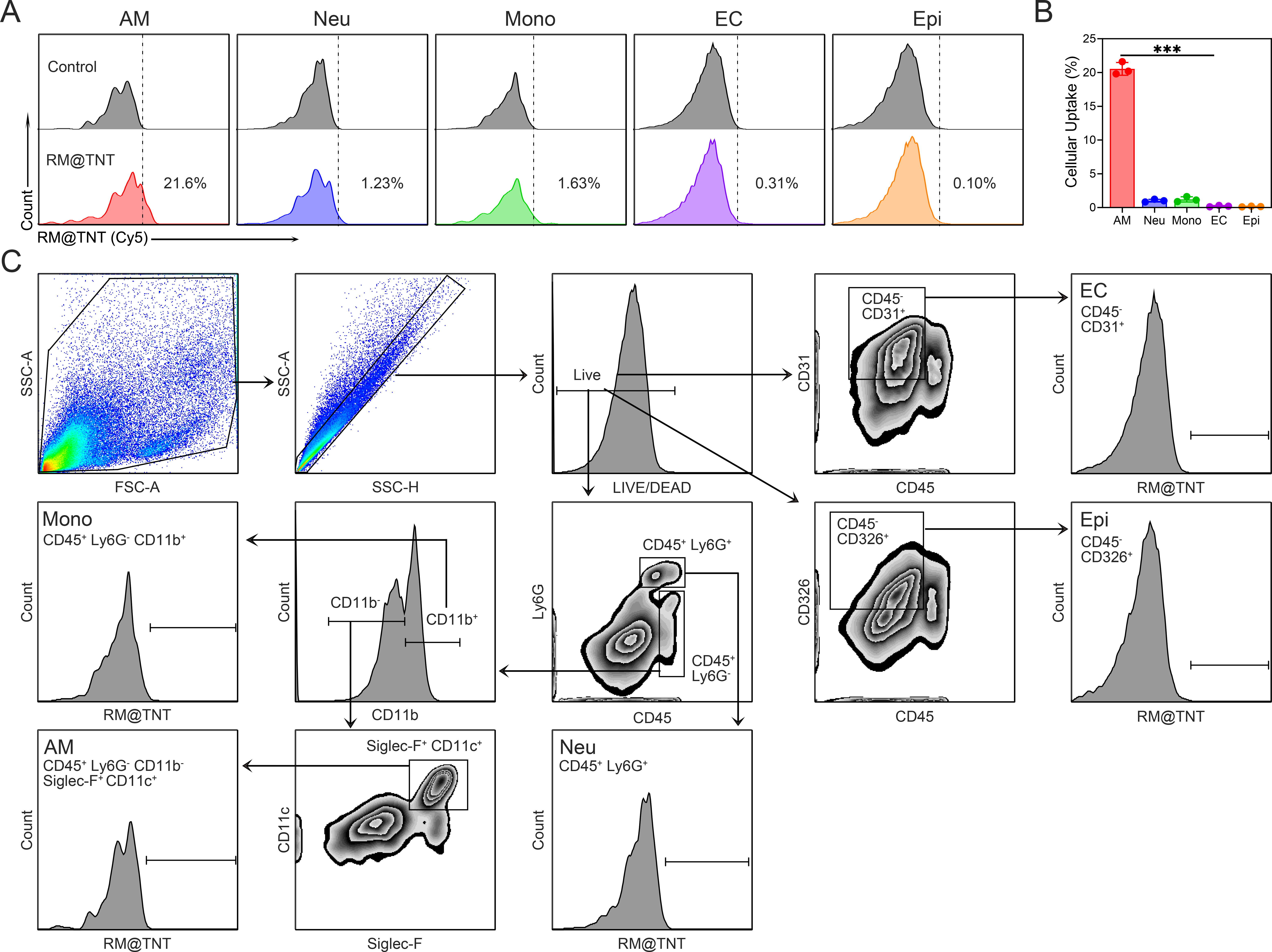
*

**Figure S19. Cellular uptake of RM@TNT (Cy5) in the lungs of SI-ALI mice following intranasal administration.** **(A)** Representative flow cytometry histograms evaluating the uptake of RM@TNT (Cy5) by distinct lung cell subsets, including AM, Neu, Mon, EC, and Epi. **(B)** The percentage of Cy5^+^ cells across the indicated cell populations (*n* = 3). **(C)** Flow cytometry gating strategy for identifying lung cell populations. Specific subsets were defined by their respective marker combinations: AM (CD45^+^/Ly6G^−^/CD11b^−^/SiglecF^+^/CD11c^+^), Mono (CD45^+^/Ly6G^−^/CD11b^+^), Neu (CD45^+^/Ly6G^+^), EC (CD45^−^/CD31^+^), and Epi (CD45^−^/CD326^+^). Quantitative data are presented as mean ± SD. Statistical significance among three groups was analyzed using one-way ANOVA with post-hoc corrections. Significance levels: ****P* < 0.001.


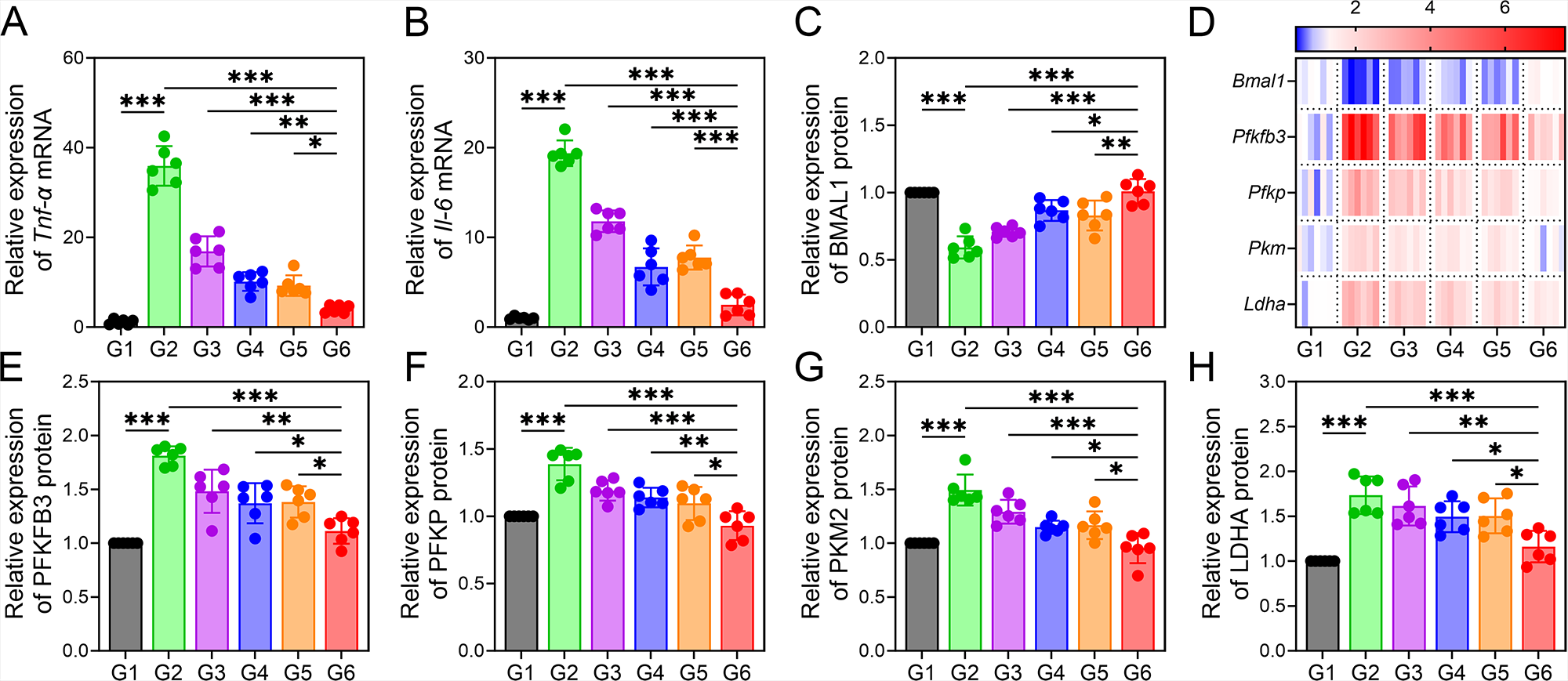


**Figure S20.** **Evaluation of the therapeutic efficacy of RM@TNT for SA-ARDS in a mouse model**. (**A, B**) Comparison of relative expression levels of *Tnf-α* and *Il-6* in lung lysates of different groups (*n* = 6). **Experimental design:** The SA-ARDS murine model was established through standard CLP, at 30 min post-CLP, intranasal administration of respective therapeutic agents (40 μL of total volume) to different experimental groups. **G1**: Sham surgery control mice; **G2**: SA-ARDS mice treated with normol saline; **G3**: SA-ARDS mice treated with Nob (10 mg·kg⁻¹); **G4**: SA-ARDS mice treated with TNT (10 mg·kg⁻¹ based on the loaded Nob); **G5**: SA-ARDS mice treated with M@TNT (10 mg·kg⁻¹ based on the loaded Nob); **G6**: SA-ARDS mice with RM@TNT (10 mg·kg⁻¹ based on the loaded Nob) treatment. (C) Western blotting analysis of BMAL1 of different groups indicated in **A-B** (*n* = 6). (S) Heatmap of relative mRNA levels (*Bmal1, Pfkfb3, Pfkp, Pkm, Ldha*) of different groups indicated in A-B (*n* = 6). (C) Western blotting analysis of PFKFB3, PFKP, PKM2, and LDHA of different groups indicated in **A-B** (*n* = 6). Quantitative data are presented as mean ± SD. Statistical significance was calculated *via* ordinary one-way ANOVA. Significance levels: **P* < 0.05, ***P* < 0.01, ****P* < 0.001.


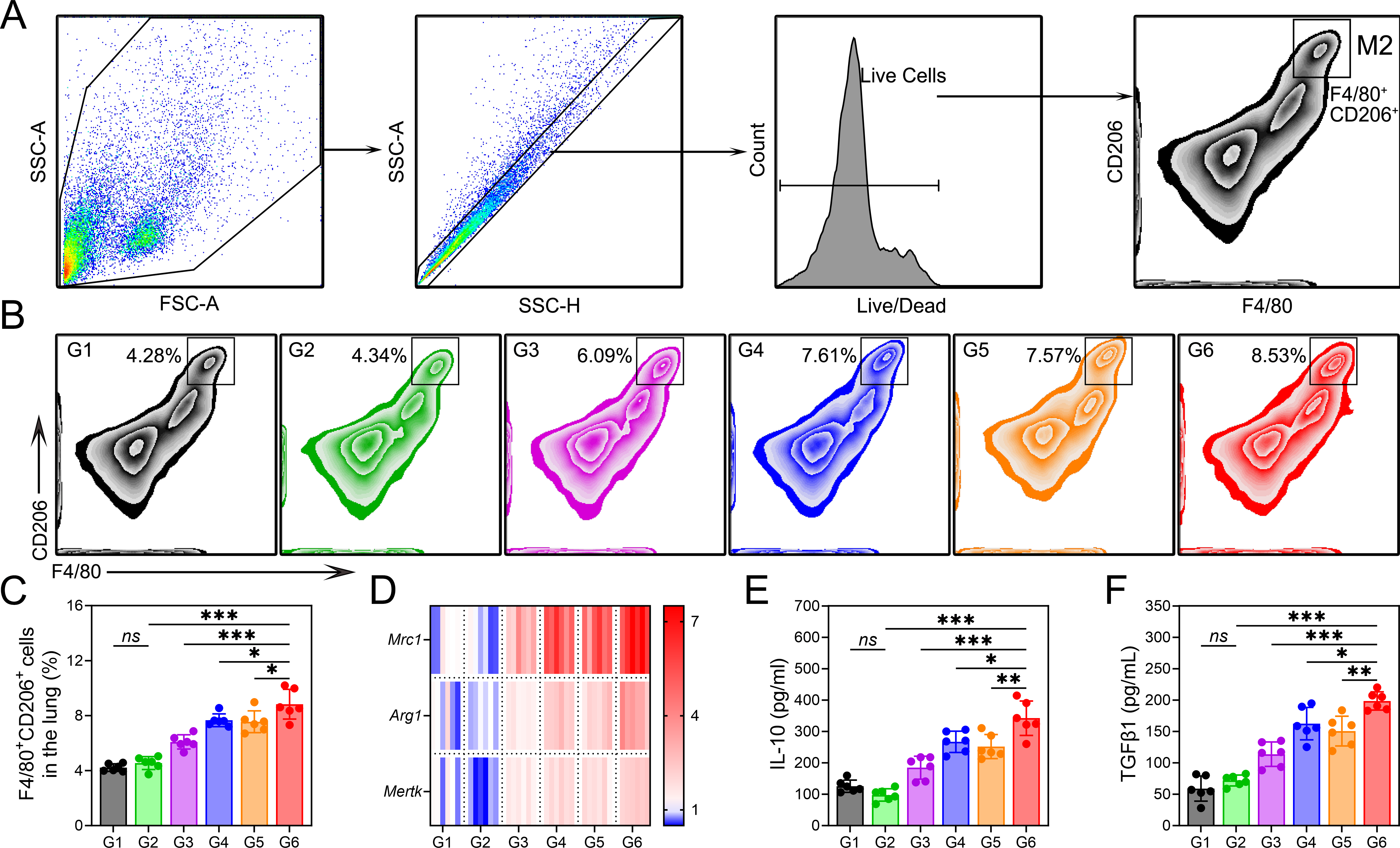


**Figure S21. The RM@TNT nanoplatform actively promotes *in vivo* M2 macrophage polarization and orchestrates a pro-resolving microenvironment in the SA-ARDS mouse model.** **Treatments:** The control group underwent laparotomy with immediate closure (the sham group, **G1**), while the remaining groups were subjected to standard CLP to induce SA-ARDS. Thirty minutes after CLP, SA-ARDS mice were intranasally administrated with 40 μL of saline (**G2**), Nob (10 mg·kg⁻¹) (**G3**), TNT (10 mg·kg⁻¹ based on Nob) (**G4**), M@TNT (10 mg·kg⁻¹ based on Nob) (**G5**), or RM@TNT (10 mg·kg⁻¹ based on Nob) (**G6**), respectively. **(A)** Flow cytometry gating strategy for identifying F4/80⁺CD206⁺ double-positive macrophages in lung tissues, including the exclusion of dead cells (live/dead discrimination). **(B)** Representative flow cytometry scatter plots showing F4/80 and CD206 expression in the lungs across different treatment groups. **(C)** Quantitative analysis of the proportion of F4/80⁺CD206⁺ M2 macrophages in the lung tissues (*n* = 6). **(D)** Heatmap illustrating the relative mRNA expression levels of M2 polarization and efferocytosis-associated genes (*Mrc1*, *Arg1*, and *Mertk*) in lung tissues assessed by RT-qPCR (*n* = 6). **(E)** Secretion levels of the anti-inflammatory cytokine IL-10 in the bronchoalveolar lavage fluid (BALF), measured by ELISA (*n* = 6). **(F)** Secretion levels of the repair-associated factor TGF-β1 in the BALF, measured by ELISA (*n* = 6). Quantitative data are presented as mean ± SD. Statistical significance was calculated *via* ordinary one-way ANOVA. Significance levels: **P* < 0.05, ***P* < 0.01, ****P* < 0.001, *ns*: no significant difference.


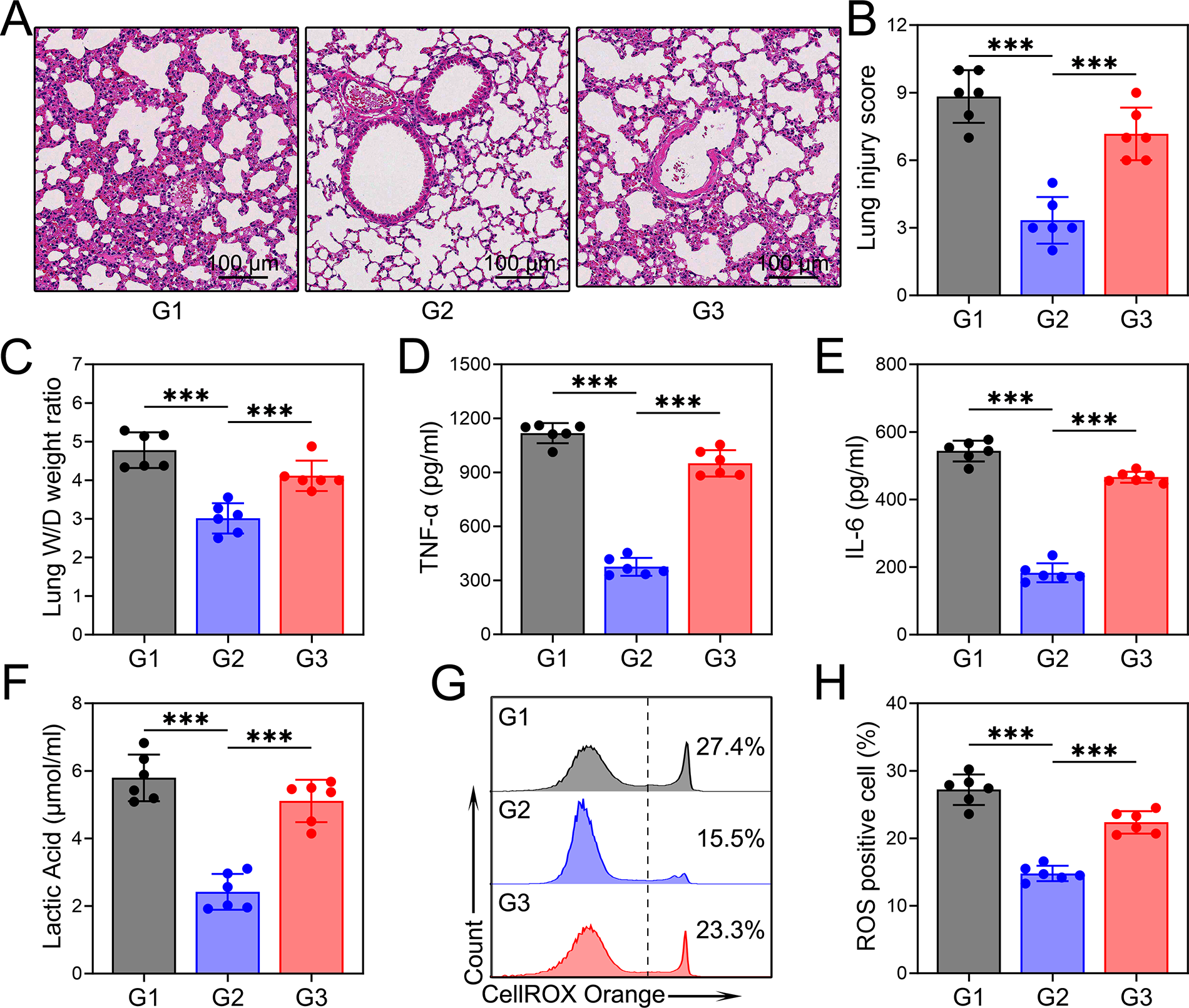


**Figure S22. The *in vivo* protective efficacy of RM@TNT is dependent on BMAL1. Treatments:** **G1**: AAV-NC-pretreated mice subjected to CLP; **G2**: AAV-NC-pretreated mice subjected to CLP and treated with RM@TNT (10 mg·kg⁻¹ based on Nob); **G3**: AAV-shBMAL1-pretreated mice subjected to CLP and treated with RM@TNT (10 mg·kg⁻¹ based on Nob). (**A**) Representative H&E staining images of lung tissues from mice after different treatments. (**B**) Quantitative analysis of the lung injury scores based on H&E pathology (*n* = 6). (**C**) Lung W/D weight ratios of mice after different treatments (*n* = 6). (**D-F**) Comparison of concentrations of TNF-α, IL-6, and lactic acid in the BALF of mice upon different treatments (*n* = 6). (**G, H**) Representative flow cytometry histograms and quantitative analysis (*n* = 6) of intracellular ROS levels in lung single-cell suspensions stained with CellROX Orange across different treatments. Quantitative data are presented as mean ± SD. Statistical significance was calculated *via* ordinary one-way ANOVA. Significance levels: ****P* < 0.001.


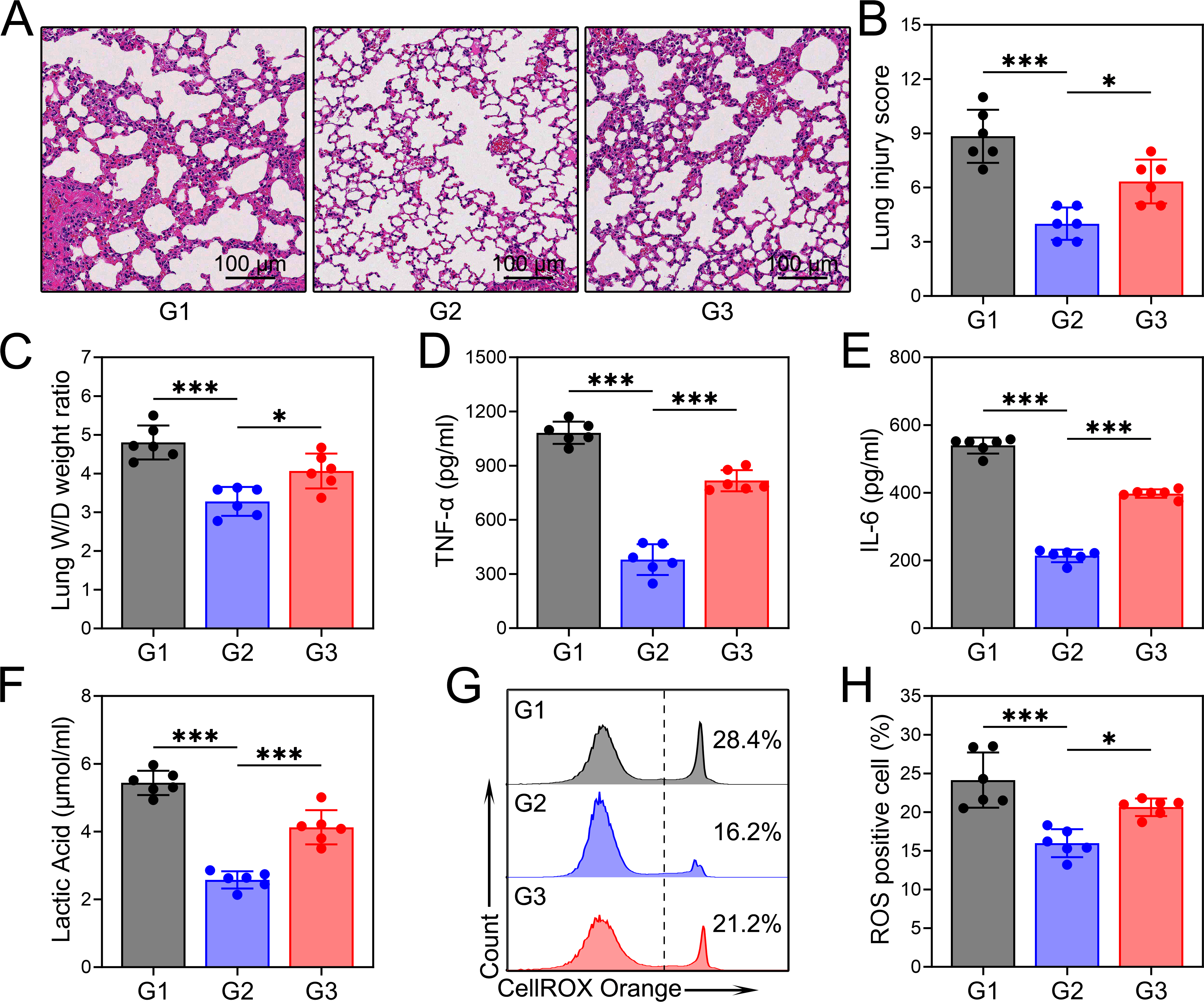


**Figure S23. *In vivo* therapeutic evaluation of RM@TNT following competitive receptor blocking.** **Treatments:** All mice were subjected to standard CLP to induce SA-ARDS. Thirty minutes after CLP, SA-ARDS mice were intranasally administrated with 40 μL of saline (**G1**), RM@TNT (10 mg·kg⁻¹ based on Nob) (**G2**), or pre-treated with excess free Tuftsin peptide (10 mg·kg⁻¹) prior to the administration of RM@TNT (10 mg·kg⁻¹ based on Nob) (**G3**), respectively. (**A**) Representative H&E staining images of lung tissues from mice after different treatments. (**B**) Quantitative analysis of the lung injury scores based on H&E pathology (*n* = 6). (**C**) Lung W/D weight ratios of mice after different treatments (*n* = 6). (**D-F**) Comparison of concentrations of TNF-α, IL-6, and lactic acid in the BALF of mice upon different treatments (*n* = 6). (**G, H**) Representative flow cytometry histograms and quantitative analysis (n = 6) of intracellular ROS levels in lung single-cell suspensions stained with CellROX Orange across different treatments. Quantitative data are presented as mean ± SD. Statistical significance was calculated *via* ordinary one-way ANOVA. Significance levels: ***P* < 0.05, ****P* < 0.001.


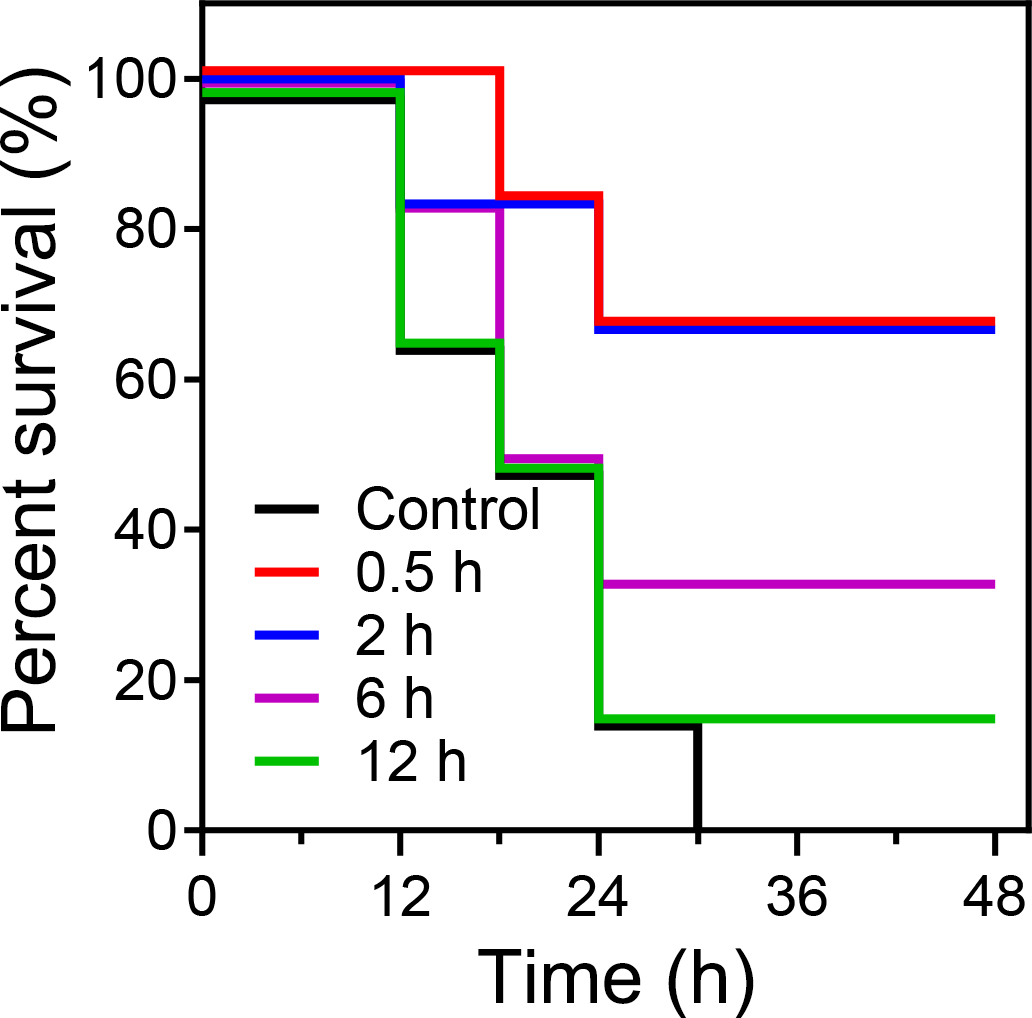


**Figure S24. Evaluation of the therapeutic time window for RM@TNT in the CLP-induced SA-ARDS mouse model.** Kaplan-Meier survival curves of CLP mice (*n* = 6 per group) receiving intranasal administration of RM@TNT (10 mg·kg⁻¹ based on Nob) at delayed time points post-CLP (0.5 h, 2 h, 6 h, and 12 h) compared to the untreated Control group.


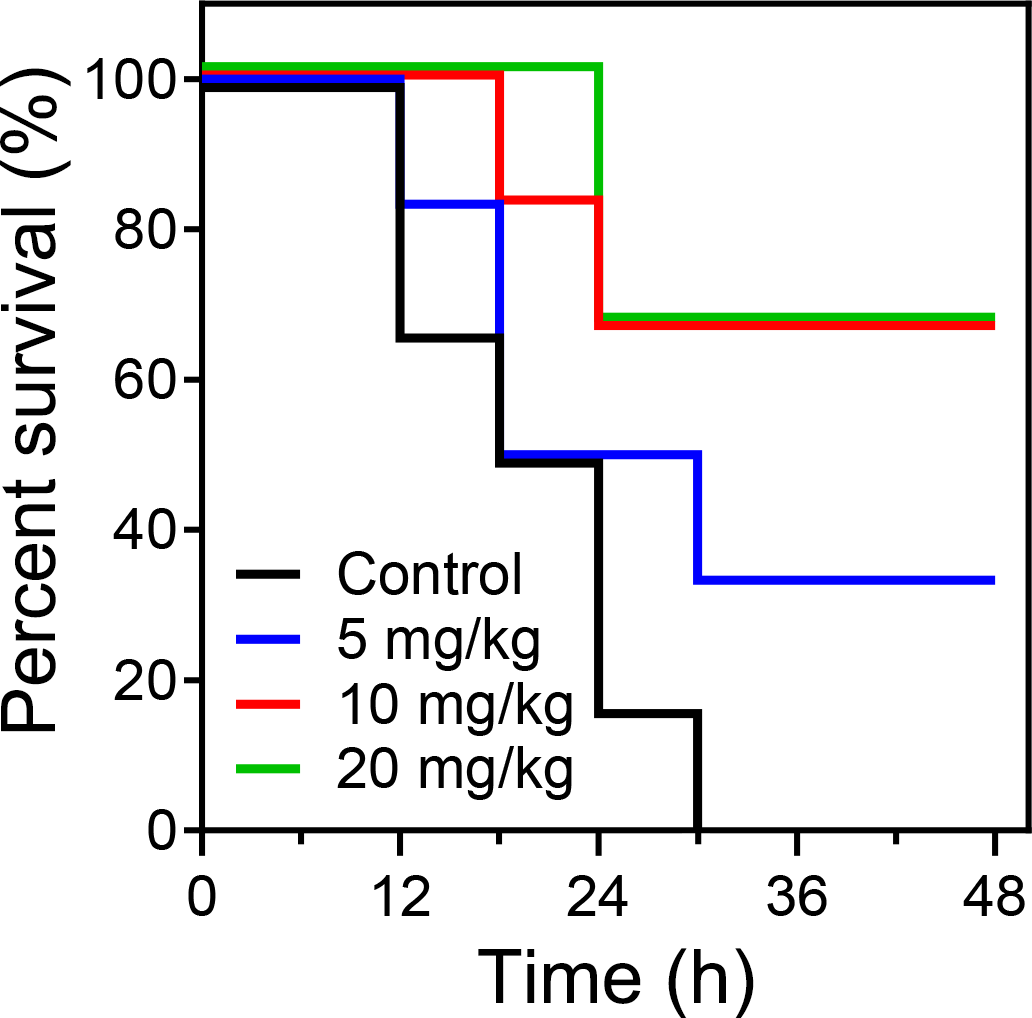


**Figure S25. Dose-response relationship of RM@TNT in the CLP-induced SA-ARDS mouse model.** Kaplan-Meier survival curves of CLP mice (*n* = 6 per group) receiving intranasal administration of RM@TNT at different therapeutic doses (5, 10, and 20 mg·kg⁻¹ based on Nob) at 0.5 h post-CLP, compared to the untreated Control group.


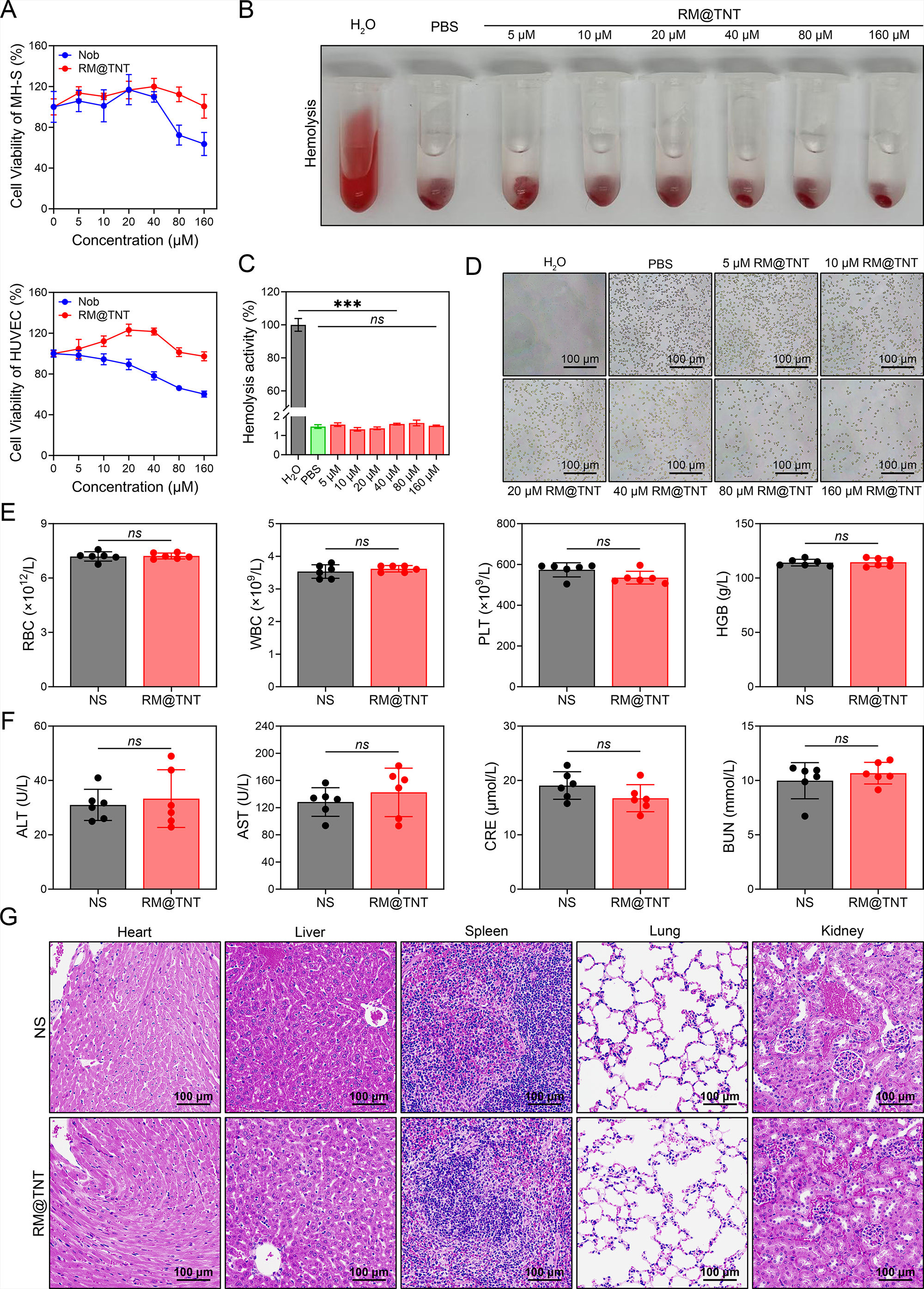


**Figure S26.** **Biosafety assessment of RM@TNT.** (**A**) Cell viability of MH-S cells and HUVECs (*n* = 4) after 24 h incubation with varying concentrations of Nob or RM@TNT (CCK‐8 assay). (**B, C**) Hemolysis analysis of fresh RBCs after 4 h incubation with different concentrations of RM@TNT (*n* = 3). (**D**) Morphological analysis of fresh red blood cells after 4-hour incubation with varying concentrations of RM@TNT by light microscopy. (**E, F**) Plasma biochemical parameters and routine blood tests in healthy mice 48 h post-intranasal administration of RM@TNT (10 mg·kg⁻¹ based on the loaded Nob) or normal saline (*n* = 6). (**G**) H&E staining of various organs from healthy mice 48 h post-intranasal administration of RM@TNT (10 mg·kg⁻¹ based on the loaded Nob) or normal saline. Quantitative data are presented as mean ± SD. Statistical significance between two groups was determined by independent samples *t*-test, while comparisons among three or more groups were analyzed using one-way ANOVA. Significance levels: **P* < 0.05, ***P* < 0.01, ****P* < 0.001; *ns*: no significant difference.


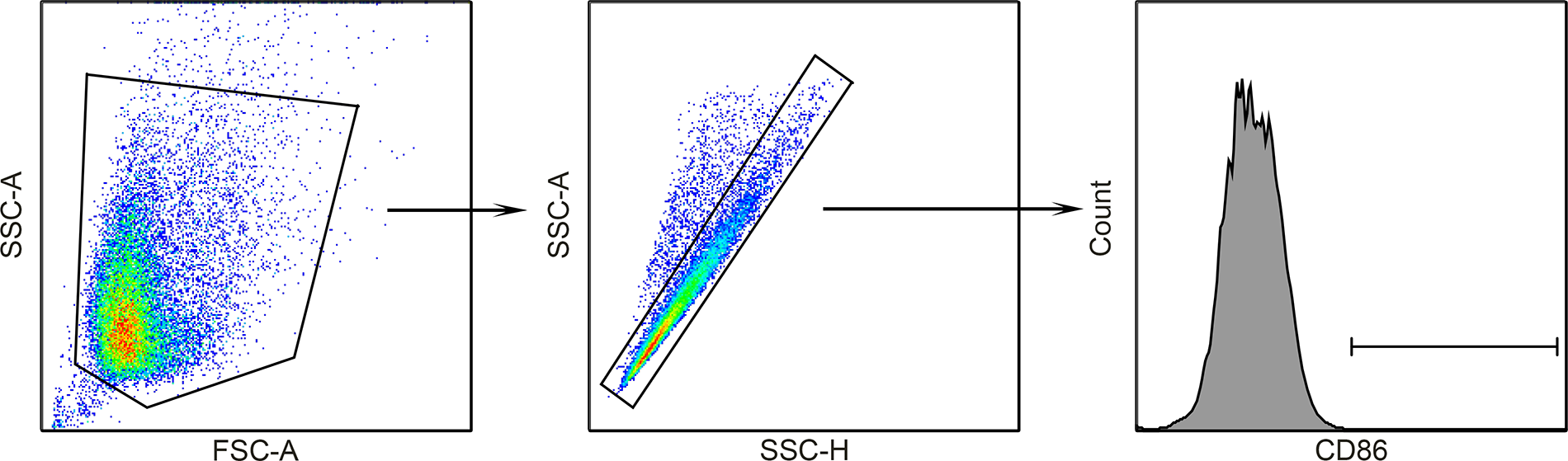


**Figure S27.** Gating strategy for the flow cytometric analysis of CD86-positive MH-S cells presented in **Figure 1N** and **Figure 3D.**


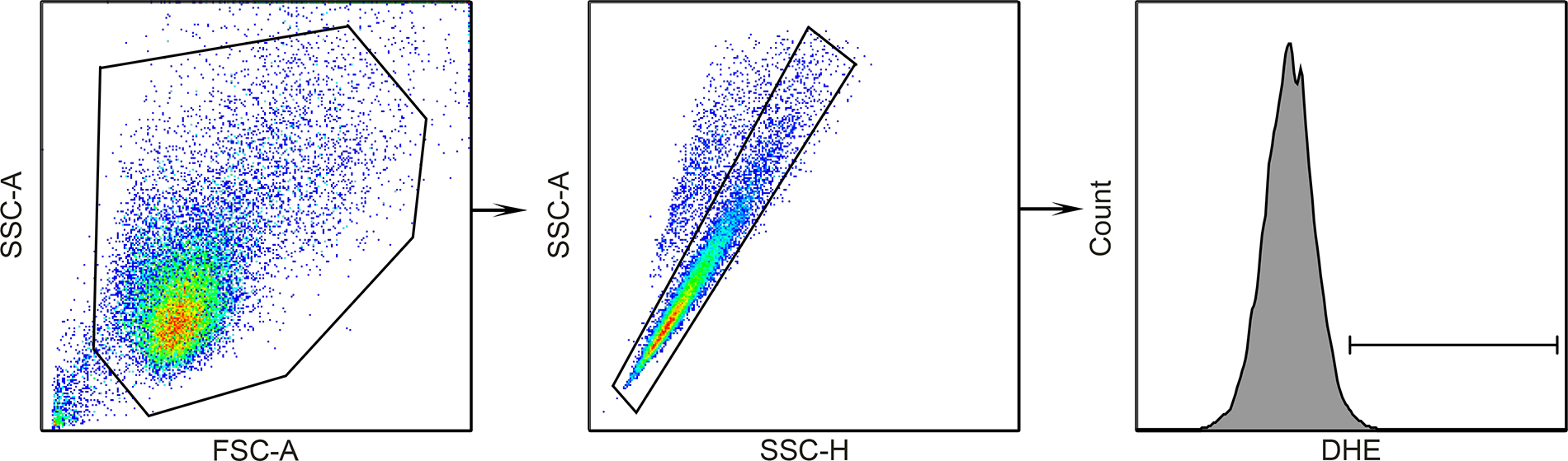


**Figure S28.** Gating strategy for the flow cytometric analysis of intracellular ROS levels *via* DHE staining in MH-S cells presented in **Figure S5C** and **Figure S10E**.


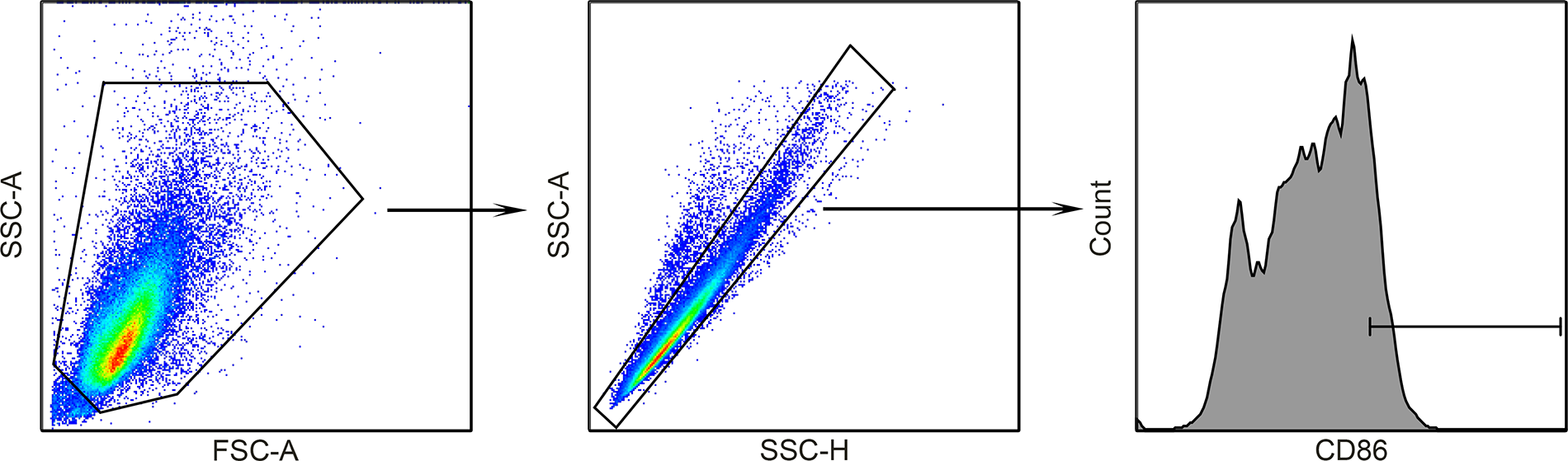


**Figure S29.** Gating strategy for the flow cytometric analysis of CD86-positive MH-S cells presented in **Figure S7E, Figure S11D** and **Figure S15D**.


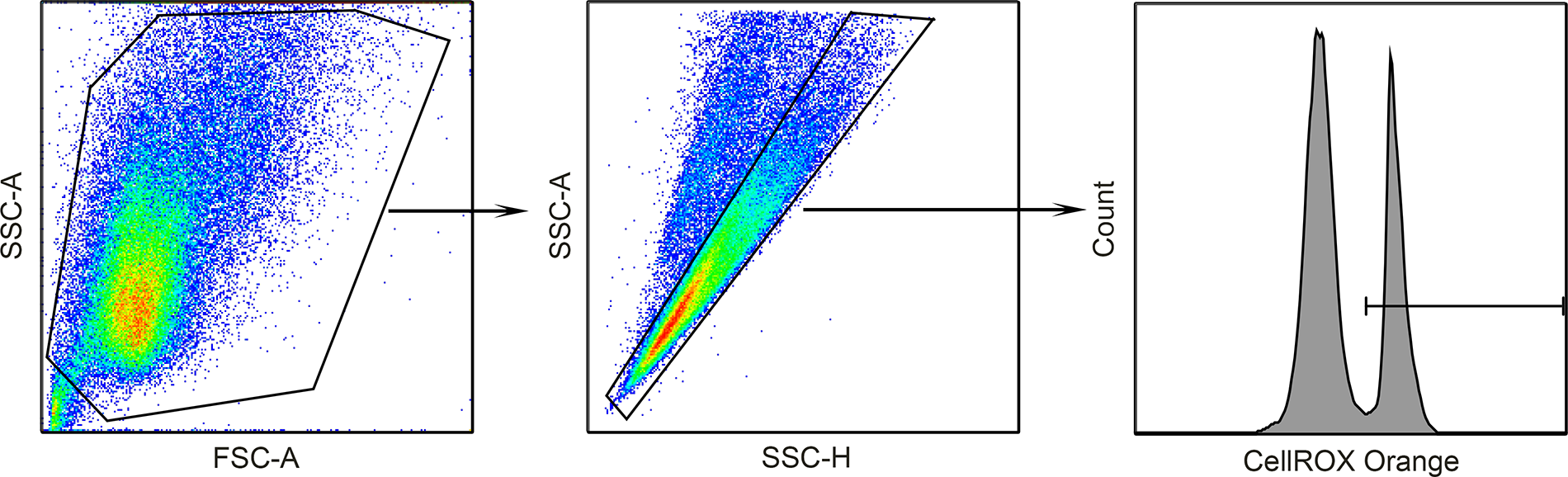


**Figure S30.** Gating strategy for the flow cytometric analysis of intracellular ROS levels *via* CellROX Orange staining in MH-S cells presented in **Figure S7I, Figure S11H** and **Figure S15H**.


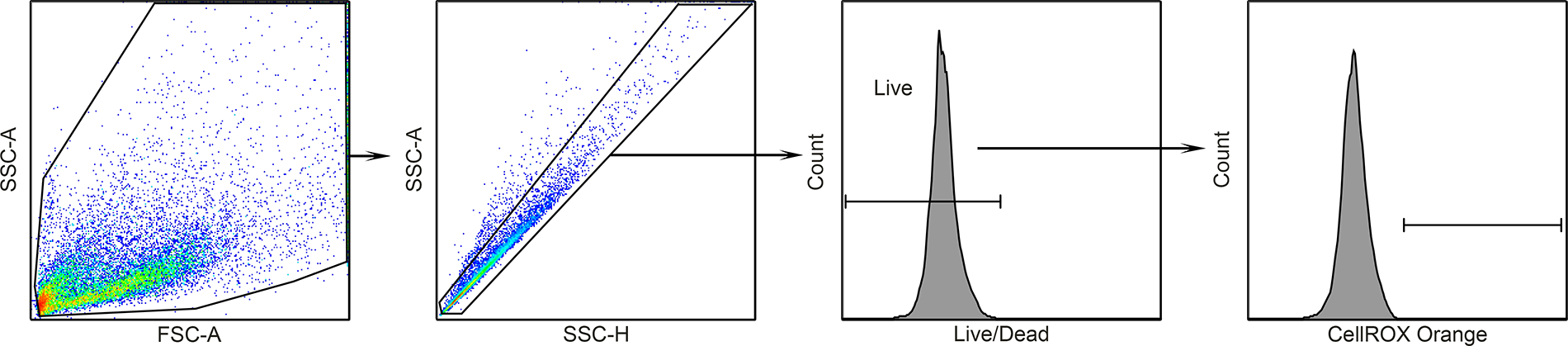


**Figure S31.** Gating strategy for the flow cytometric analysis of intracellular ROS levels *via* CellROX Orange staining in mouse lung single-cell suspensions presented in **Figure S8H, Figure S22G** and **Figure S23G**.


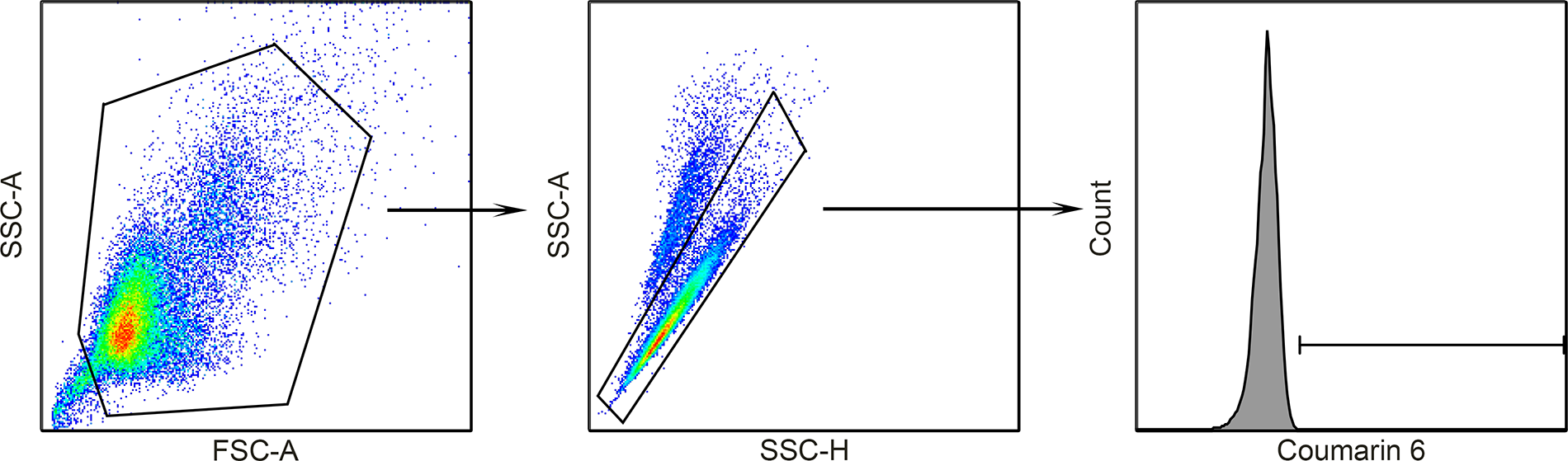


**Figure S32.** Gating strategy for the flow cytometric analysis of Coumarin-6 uptake by MH-S cells presented in **Figure 4M** and **Figure 4Q**.


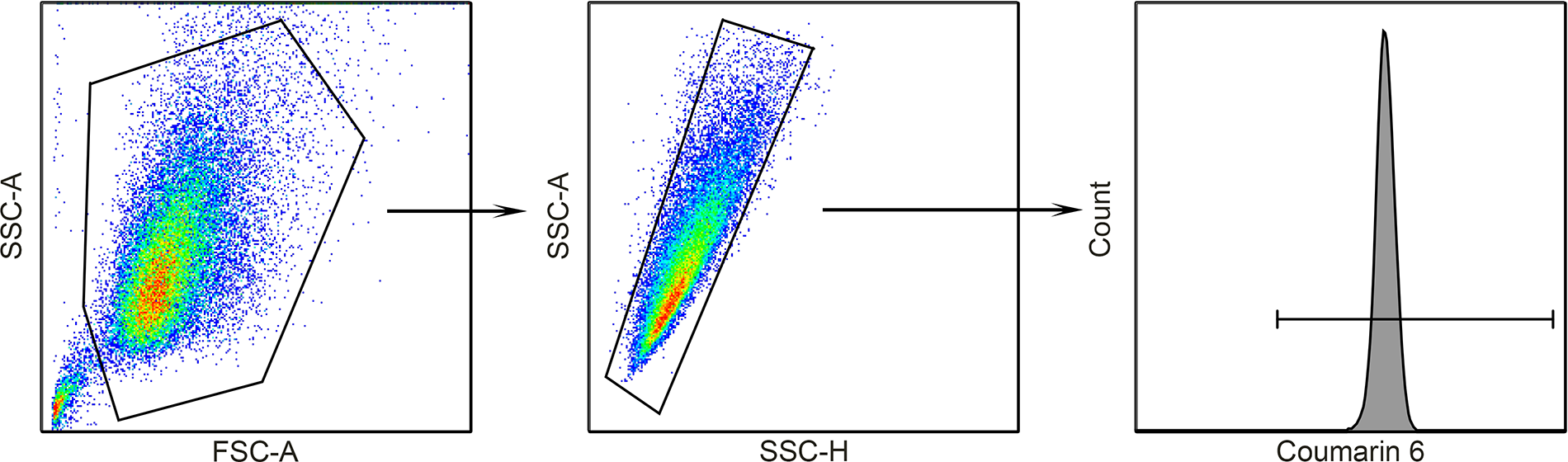


**Figure S33.** Gating strategy for the flow cytometric analysis of Coumarin-6 uptake by HUVECs presented in **Figure 4O**.


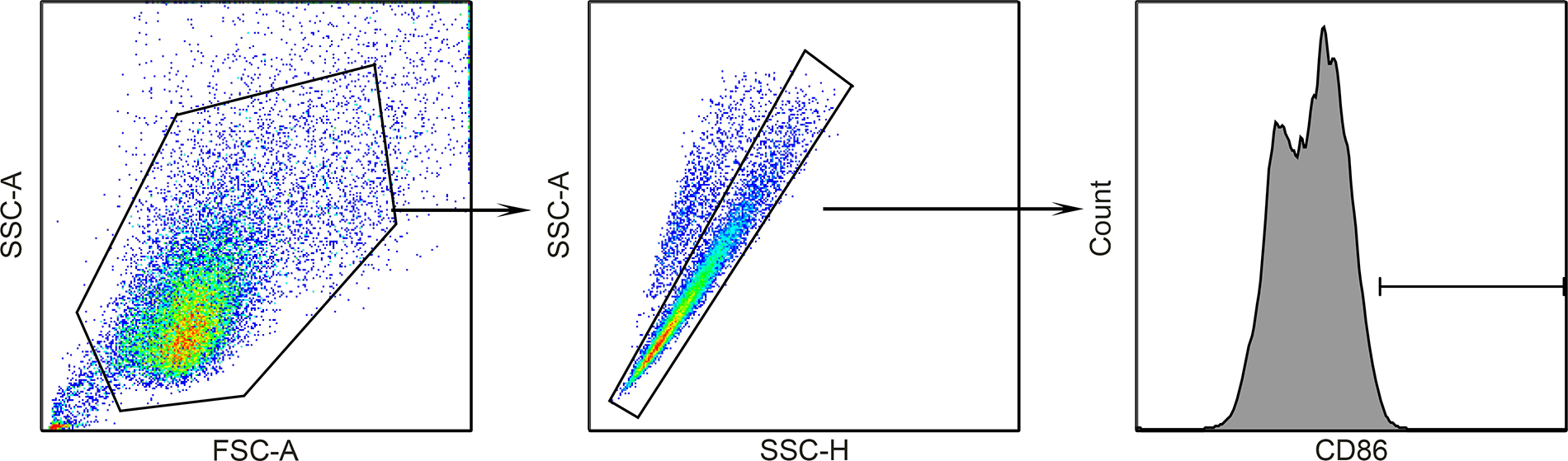


**Figure S34.** Gating strategy for the flow cytometric analysis of CD86-positive MH-S cells presented in **Figure S5I**.


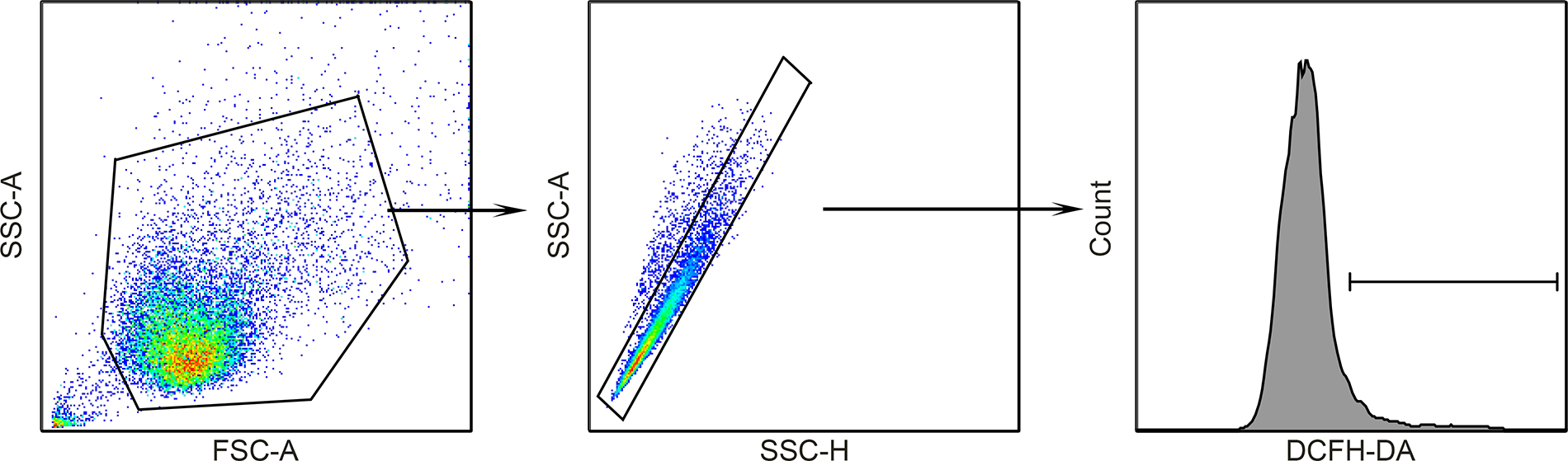


**Figure S35.** Gating strategy for the flow cytometric analysis of intracellular ROS levels *via* DCFH-DA staining in MH-S cells presented in **Figure 5M**.


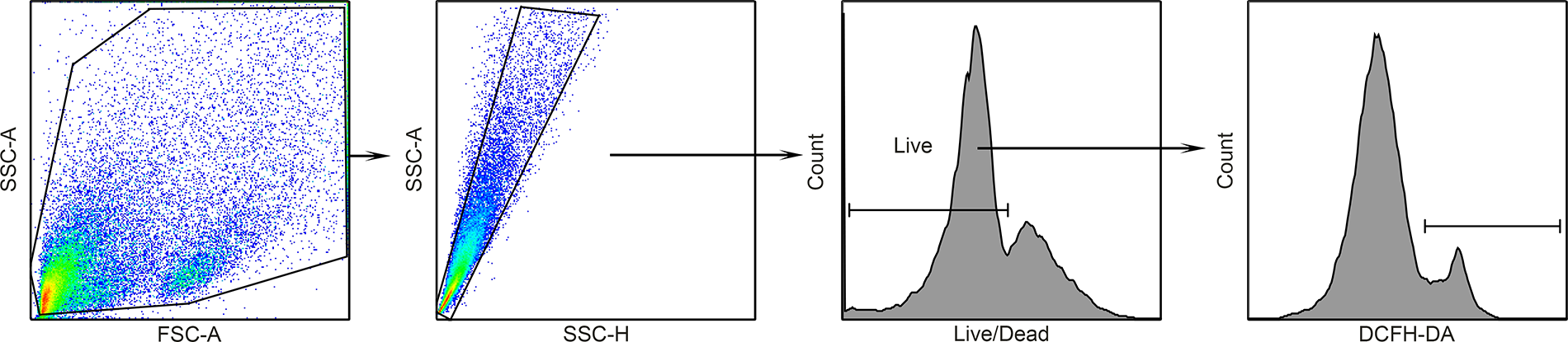


**Figure S36.** Gating strategy for the flow cytometric analysis of intracellular ROS levels *via* DCFH-DA staining in mouse lung single-cell suspensions presented in **Figure 7H**.


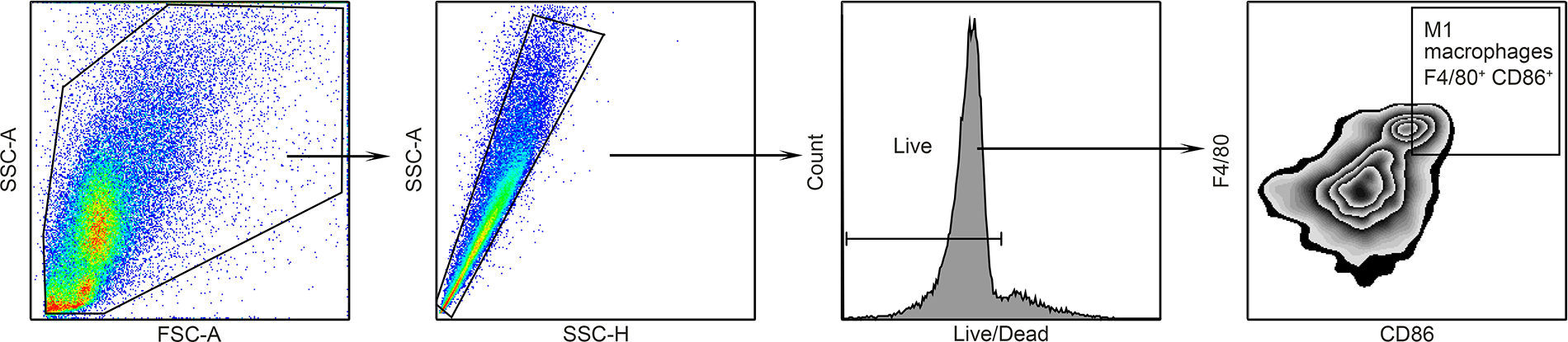


**Figure S37**. Gating strategy for the flow cytometric analysis of M1 macrophages (F4/80^+^ CD86^+^) in mouse lung single-cell suspensions presented in **Figure 7L**.

**Table S1.** List of sequences for TDN.

| Name | Sequence |
| --- | --- |
| S1 | ATTTATCACCCGCCATAGTAGACGTATCACCAGGCAGTTGAGACGAACATTCCTAAGTCTGAA |
| S2 | ACATGCGAGGGTCCAATACCGACGATTACAGCTTGCTACACGATTCAGACTTAGGAATGTTCG |
| S3 | ACTACTATGGCGGGTGATAAAACGTGTAGCAAGCTGTAATCGACGGGAAGAGCATGCCCATCC |
| S4 | ACGGTATTGGACCCTCGCATGACTCAACTGCCTGGTGATACGAGGATGGGCATGCTCTTCCCG |
| Cy5-S1 | Cy5- ATTTATCACCCGCCATAGTAGACGTATCACCAGGCAGTTGAGACGAACATTCCTAAGTCTGAA |

**Table S2.** List of primers for qPCR.

| Name | Direction | Sequence (5’→3’) |
| --- | --- | --- |
| *mPfkp* | FORWARD | 5′-CAGAGCCACCAGAGGACCTTC-3′ |
|  | REVERSE | 5'-GTCGGCACCGCAAGTCAAG-3' |
| *mPkm* | FORWARD | 5'-TGTAAGGATGCCGTGCTCAATGC-3' |
|  | REVERSE | 5′-ACAATGACCACATCTCCCTTCTTGAAG-3′ |
| *mPfkfb3* | FORWARD | 5'-TTGTCCAGCAGAGGCAAGAAGTTC-3' |
|  | REVERSE | 5'-TCCACACACGGAGGTCCTTCAG-3' |
| *mLdha* | FORWARD | 5'-CGGCTGGGTCCTGGGAGAAC-3' |
|  | REVERSE | 5′-ACCTCCTTCCACTGCTCCTTGTC-3′ |
| *mBmal1* | FORWARD | 5′-CGTGCTAAGGATGGCTGTTCAG-3′ |
|  | REVERSE | 5'-AAATGTTGGCTTGTAGTTTGCTTCTG-3' |
| m*Tnf-α* | FORWARD | 5'-CAGGCGGTGCCTATGTCTC-3' |
|  | REVERSE | 5'-CGATCACCCCGAAGTTCAGTAG-3' |
| m*Il-6* | FORWARD | 5'-CTTGGGACTGATGCTG-3' |
|  | REVERSE | 5'-TTTCTCATTTCCACGAT-3' |
| m*Actb* | FORWARD | 5′-GATTACTGCTCTGGCTCCTAGC-3′ |
|  | REVERSE | 5′-GACTCATCGTACTCCTGCTTGC-3′ |
| *mArg1* | FORWARD | 5′-CTCCAAGCCAAAGTCCTTAGAG-3′ |
|  | REVERSE | 5′-AGGAGCTGTCATTAGGGACATC-3′ |
| *mMrc1* | FORWARD | 5′-CTCTGTTCAGCTATTGGACGC-3′ |
|  | REVERSE | 5′-CGGAATTTCTGGGATTCAGCTTC-3′ |
| m*Mertk* | FORWARD | 5′-AGCTGGCATTTCATGGTGGA-3′ |
|  | REVERSE | 5′-TGCACACTGGCTATGCTGAA-3′ |
| m*Hif1a* | FORWARD | 5′-TCAAGTCAGCAACGTGGAAG-3′ |
|  | REVERSE | 5′-TATCGAGGCTGTGTCGACTG-3′ |
| m*Myc* | FORWARD | 5′-AAGAGGGCCAAGTTGGACAG-3′ |
|  | REVERSE | 5′-ATTCAGGGATCTGGTCACGC-3′ |
| m*Mtor* | FORWARD | 5′-TCCGAGAGATGAGTCAAGAGG-3′ |
|  | REVERSE | 5′-CACCTTCCACTCCTATGAGGC-3′ |
